# The water lily genome and the early evolution of flowering plants

**DOI:** 10.1038/s41586-019-1852-5

**Published:** 2019-12-18

**Authors:** Liangsheng Zhang, Fei Chen, Xingtan Zhang, Zhen Li, Yiyong Zhao, Rolf Lohaus, Xiaojun Chang, Wei Dong, Simon Y. W. Ho, Xing Liu, Aixia Song, Junhao Chen, Wenlei Guo, Zhengjia Wang, Yingyu Zhuang, Haifeng Wang, Xuequn Chen, Juan Hu, Yanhui Liu, Yuan Qin, Kai Wang, Shanshan Dong, Yang Liu, Shouzhou Zhang, Xianxian Yu, Qian Wu, Liangsheng Wang, Xueqing Yan, Yuannian Jiao, Hongzhi Kong, Xiaofan Zhou, Cuiwei Yu, Yuchu Chen, Fan Li, Jihua Wang, Wei Chen, Xinlu Chen, Qidong Jia, Chi Zhang, Yifan Jiang, Wanbo Zhang, Guanhua Liu, Jianyu Fu, Feng Chen, Hong Ma, Yves Van de Peer, Haibao Tang

**Affiliations:** 1 Fujian Provincial Key Laboratory of Haixia Applied Plant Systems Biology, Key Laboratory of Ministry of Education for Genetics, Breeding and Multiple Utilization of Crops, Key Laboratory of National Forestry and Grassland Administration for Orchid Conservation and Utilization, Fujian Agriculture and Forestry University, Fuzhou, China; 2 College of Horticulture, Nanjing Agricultural University, Nanjing, China; 3 Department of Plant Biotechnology and Bioinformatics, Ghent University, Ghent, Belgium; 4 VIB Center for Plant Systems Biology, Ghent, Belgium; 5 State Key Laboratory of Genetic Engineering, Ministry of Education Key Laboratory of Biodiversity Sciences and Ecological Engineering, School of Life Sciences, Fudan University, Shanghai, China; 6 Department of Biology, Huck Institutes of the Life Sciences, Pennsylvania State University, University Park, PA USA; 7 Fairy Lake Botanical Garden, Shenzhen and Chinese Academy of Sciences, Shenzhen, China; 8 School of Life and Environmental Sciences, University of Sydney, Sydney, New South Wales Australia; 9 State Key Laboratory of Subtropical Silviculture, School of Forestry and Biotechnology, Zhejiang A&F University, Hangzhou, China; 10 BGI-Shenzhen, Shenzhen, China; 11 School of Urban-Rural Planning and Landscape Architecture, Xuchang University, Xuchang, China; 12 Key Laboratory of Plant Resources/Beijing Botanical Garden, Institute of Botany, Chinese Academy of Sciences, Beijing, China; 13 University of the Chinese Academy of Sciences, Beijing, China; 14 State Key Laboratory of Systematic and Evolutionary Botany, Institute of Botany, Chinese Academy of Sciences, Beijing, China; 15 Guangdong Province Key Laboratory of Microbial Signals and Disease Control, Integrative Microbiology Research Centre, South China Agricultural University, Guangzhou, China; 16 Hangzhou Tianjing Aquatic Botanical Garden, Zhejiang Humanities Landscape Co. Ltd., Hangzhou, China; 17 National Engineering Research Center for Ornamental Horticulture, Key Laboratory for Flower Breeding of Yunnan Province, Floriculture Research Institute, Yunnan Academy of Agricultural Sciences, Kunming, China; 18 Innovative Institute of Chinese Medicine and Pharmacy, Chengdu University of Traditional Chinese Medicine, Chengdu, China; 19 Department of Plant Sciences, University of Tennessee, Knoxville, TN USA; 20 Graduate School of Genome Science and Technology, University of Tennessee, Knoxville, TN USA; 21 Key Laboratory of Tea Quality and Safety Control, Ministry of Agriculture and Rural Affairs, Tea Research Institute, Chinese Academy of Agricultural Sciences, Hangzhou, China; 22 Centre for Microbial Ecology and Genomics, Department of Biochemistry, Genetics and Microbiology, University of Pretoria, Pretoria, South Africa

**Keywords:** Molecular evolution, Genome evolution, Plant evolution

## Abstract

Water lilies belong to the angiosperm order Nymphaeales. Amborellales, Nymphaeales and Austrobaileyales together form the so-called ANA-grade of angiosperms, which are extant representatives of lineages that diverged the earliest from the lineage leading to the extant mesangiosperms^1–3^. Here we report the 409-megabase genome sequence of the blue-petal water lily (*Nymphaea colorata*). Our phylogenomic analyses support Amborellales and Nymphaeales as successive sister lineages to all other extant angiosperms. The *N. colorata* genome and 19 other water lily transcriptomes reveal a Nymphaealean whole-genome duplication event, which is shared by Nymphaeaceae and possibly Cabombaceae. Among the genes retained from this whole-genome duplication are homologues of genes that regulate flowering transition and flower development. The broad expression of homologues of floral ABCE genes in *N. colorata* might support a similarly broadly active ancestral ABCE model of floral organ determination in early angiosperms. Water lilies have evolved attractive floral scents and colours, which are features shared with mesangiosperms, and we identified their putative biosynthetic genes in *N. colorata*. The chemical compounds and biosynthetic genes behind floral scents suggest that they have evolved in parallel to those in mesangiosperms. Because of its unique phylogenetic position, the *N. colorata* genome sheds light on the early evolution of angiosperms.

## Abstract (web summary)

The genome of the tropical blue-petal water lily *Nymphaea colorata* and the transcriptomes from 19 other Nymphaeales species provide insights into the early evolution of angiosperms.

## Main

Many water lily species, particularly from *Nymphaea* (Nymphaeaceae), have large and showy flowers and belong to the angiosperms (also called flowering plants). Their aesthetic beauty has captivated notable artists such as the French impressionist Claude Monet. Water lily flowers have limited differentiation in perianths (outer floral organs), but they possess both male and female organs and have diverse scents and colours, similar to many mesangiosperms (core angiosperms, including eudicots, monocots, and magnoliids) (Supplementary Note 1). In addition, some water lilies have short life cycles and enormous numbers of seeds^4^, which increase their potential as a model plant to represent the ANA-grade of angiosperms and to study early evolutionary events within the angiosperms. In particular, *N. colorata* Peter has a relatively small genome size (2*n* = 28 and approximately 400 Mb) and blue petals that make it popular in breeding programs (Supplementary Note 1).

**Extended Data Fig. 1:**
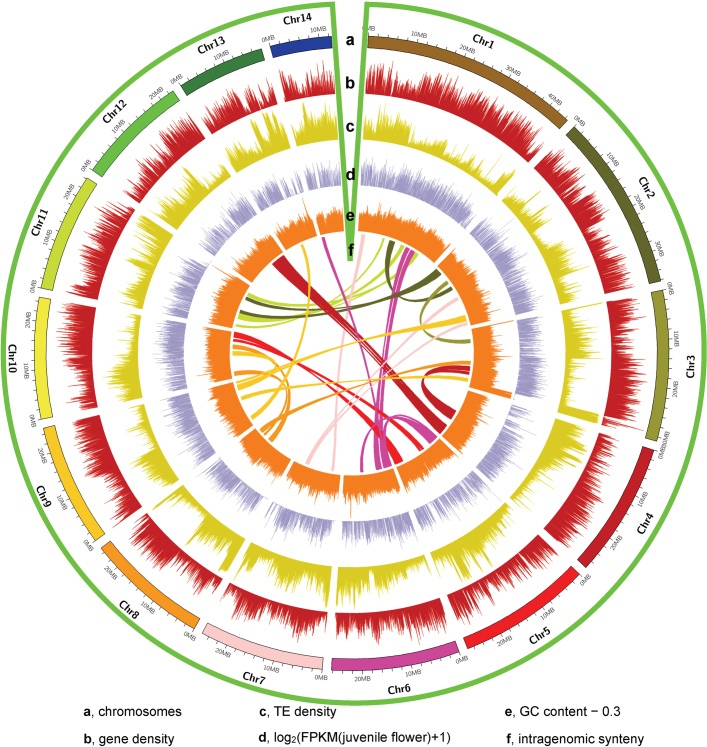
High-quality genome of *N. colorata* allows integration of genetic and expression data. **a**, The assembled 14 chromosomes. **b**, Gene density plotted in a 100-kb sliding window. **c**, Transposable element (TE) density plotted in a 100-kb sliding window. **d**, Gene expression atlas of the juvenile flower, expression values were transformed with log_2_(FPKM + 1). **e**, GC content plotted in a 100-kb sliding window. **f**, Intragenomic syntenic regions denoted by a single line represent a genomic syntenic region covering at least 20 paralogues.

We report here the genome sequence of *N. colorata*, obtained using PacBio RSII single-molecule real-time (SMRT) sequencing technology. The genome was assembled into 1,429 contigs (with a contig N50 of 2.1 Mb) and total length of 409 Mb with 804 scaffolds, 770 of which were anchored onto 14 pseudo-chromosomes (Extended Data Fig. 1 and Extended Data Table 1). Genome completeness was estimated to be 94.4% (Supplementary Note 2). We annotated 31,580 protein-coding genes and predicted repetitive elements with a collective length of 160.4 Mb, accounting for 39.2% of the genome (Supplementary Note 3).

**Fig. 1:**
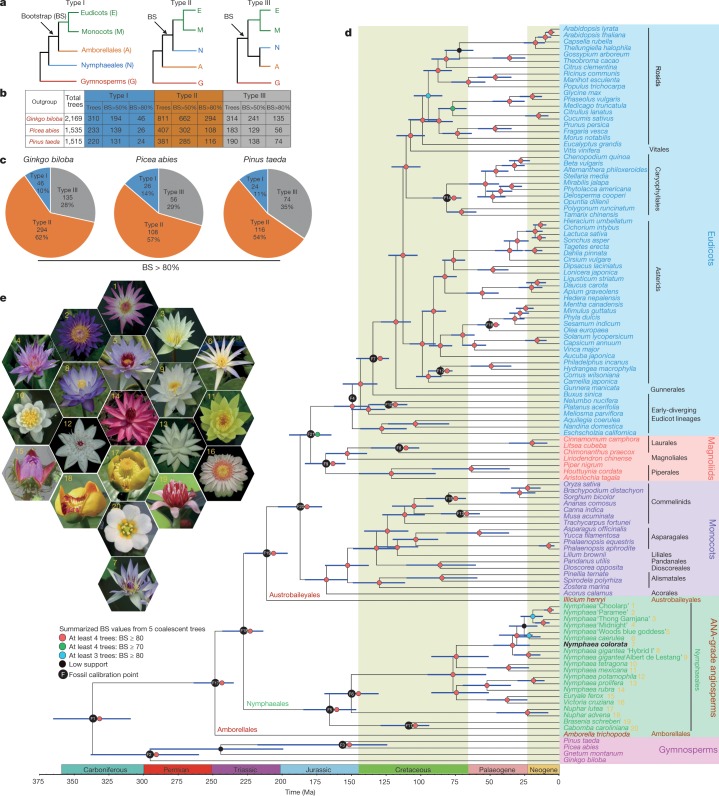
Phylogenomic relationships of angiosperms. **a**, Three different evolutionary relationships among major clades of angiosperms. **b**, Number of LCN gene trees with different bootstrap support (BS) values based on nucleotide sequences from six eudicots, six monocots, *N. colorata*, *Amborella* and three different gymnosperms. **c**, Comparison of gene trees supporting the three evolutionary relationships using each gymnosperm in turn as the outgroup. The percentage was calculated by dividing the number of type I, II or III trees (BS > 80%) by the total number of trees. **d**, Summary phylogeny and timescale of 115 plant species. Blue bars at nodes represent 95% credibility intervals of the estimated dates. **e**, The flowers of the 20 sampled water lilies in Nymphaeales used in **d**.

The *N. colorata* genome provides an opportunity to resolve the relationships between Amborellales, Nymphaeales and all other extant angiosperms (Fig. 1a). Using six eudicots, six monocots, *N. colorata* and *Amborella*^5^, and each of three gymnosperm species (*Ginkgo biloba*, *Picea abies* and *Pinus taeda*) as an outgroup in turn, we identified 2,169, 1,535 and 1,515 orthologous low-copy nuclear (LCN) genes, respectively (Fig. 1b). Among the LCN gene trees inferred from nucleotide sequences using *G. biloba* as an outgroup, 62% (294 out of 475 trees) place *Amborella* as the sister lineage to all other extant angiosperms with bootstrap support greater than 80% (type II, Fig. 1c). Using *P. abies* or *P. taeda* as the outgroup, *Amborella* is placed as the sister lineage to the remaining angiosperms in 57% and 54% of the LCN gene trees, respectively. LCN gene trees inferred using amino acid sequences show similar phylogenetic patterns (Supplementary Note 4.1).

To minimize the potential shortcomings of sparse taxon sampling^6^, we also inferred an angiosperm species tree using sequences from 44 genomes and 71 transcriptomes, including representatives of the ANA-grade, eudicots, magnoliids, monocots and a gymnosperm outgroup (*Gnetum montanum*, *G. biloba*, *P. abies* and *P. taeda*) (Methods). For further phylogenetic inference of these 115 species, we selected, based on various criteria, five different LCN gene sets including 1,167, 834, 683, 602 and 445 genes. Analyses of these five datasets all yielded similar tree topologies with *Amborella* and Nymphaeales as successive sister lineages to all other extant angiosperms (Fig. 1d, e, Supplementary Note 4.2).

Molecular dating of angiosperm lineages, using a stringent set of 101 LCN genes and with age calibrations based on 21 fossils^7^, inferred the crown age of angiosperms at 234–263 million years ago (Ma) (Fig. 1d). The split between monocots and eudicots was estimated at 171–203 Ma and that between Nymphaeaceae and Cabombaceae at 147–185 Ma.

**Fig. 2:**
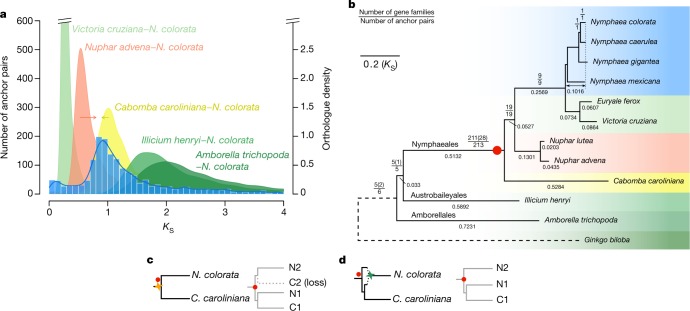
A Nymphaealean WGD shared by Nymphaeaceae and possibly Cabombaceae. **a**, *K*_S_ age distributions for paralogues found in collinear regions (anchor pairs) of *N. colorata* and for orthologues between *N. colorata* and selected Nymphaealean and angiosperm species. Red and yellow arrows indicate under- and overestimations of the *N. colorata*–*Nuphar advena* and *N. colorata*–*C. caroliniana* divergence, respectively. **b**, WGD phylogenomic analysis. Numbers in parentheses are the number of gene families with retained *C. caroliniana* duplicates supporting the duplication events. Numbers below branches show branch lengths in *K*_S_ units. The double-arrowed line denotes total *K*_S_ from the pointed node to *N. colorata*. We used *G.* *biloba* (dashed branch) as an outgroup. The red dot denotes the branch on which most of the anchor pairs in *N. colorata* coalesced. All mapped duplication events have BS ≥ 80% in the gene trees. **c**, Left, the scenario of a WGD (yellow four-pointed star) before the divergence between Nymphaeaceae and Cabombaceae. Right, a possible gene tree under this scenario, with loss of one duplicate in *C. caroliniana*. Two red dots show where the anchor pair of *N. colorata* would coalesce. **d**, Left, scenario of a WGD in the stem lineage of Nymphaeaceae involving an allotetraploid (green four-pointed star) that formed between two ancestral parents after the divergence of the lineages leading to *N. colorata* and *C. caroliniana*, with one of the parents being more closely related to *C. caroliniana*. Right, a gene tree under such a scenario. Red dots are as in **c**.

**Extended Data Fig. 2:**
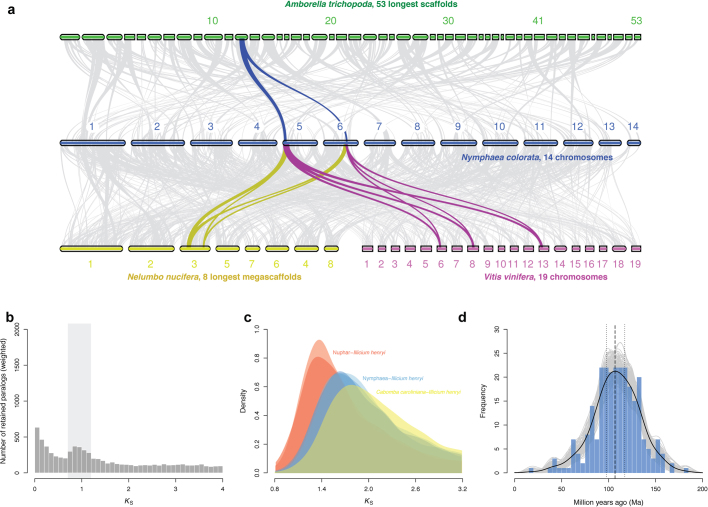
WGD in Nymphaeales. **a**, Intergenomic synteny between *N. colorata* (14 chromosomes), *Amborella* (53 longest scaffolds), and the eudicots *N. nucifera* (8 longest megascaffolds) and *V. vinifera* (19 chromosomes). Five adjacent anchor pairs were plotted as one syntenic line. Coloured lines represent one example of syntenic genes found in other species that correspond to one copy in *Amborella*, two in *N. colorata*, two in *N. nucifera*, and three in *V. vinifera*. **b**, *K*_S_ distribution for the whole paranome of *N. colorata*. The light grey rectangle in the background indicates the *K*_S_ boundaries used to extract duplicate pairs for absolute phylogenomic dating of the WGD event, and also highlights the range in which WGD peaks can be identified in other species of Nymphaeaceae (Supplementary Note 5.2). **c**, Kernel-density estimates of *K*_S_ distributions for one-to-one orthologues between the outgroup species *I. henryi* and each of *N. lutea* and *N. advena* (red), *N. colorata*, *N. mexicana* and *Nymphaea* ‘Woods blue goddess’ (blue) and *C. caroliniana* (yellow). As each peak represents the same divergence event in the angiosperm phylogeny, the differences observed among the *K*_S_ values of the peaks indicate substantial substitution rate variation among these Nymphaealean lineages (see also Fig. 2b). **d**, Absolute age distribution obtained from phylogenomic dating of *N. colorata* WGD paralogues based on orthogroups with orthologues from *Amborella* and *G. biloba*. The solid black line represents the kernel density estimate of paralogue date estimates, and the vertical dashed black line represents its peak at 107 Ma. The grey lines represent density estimates from 2,500 bootstrap replicates and the vertical black dotted lines represent the corresponding 90% confidence interval for the WGD age estimate, 117–98 Ma (see Methods). The blue histogram shows the raw distribution of divergence date estimates for paralogues.

Genomic collinearity unveiled evidence of a whole-genome duplication (WGD) event in *N. colorata* (Extended Data Figs. 1f, 2a and Supplementary Note 5.1). The number of synonymous substitutions per synonymous site (*K*_S_) distributions for *N. colorata* paralogues further showed a signature peak at *K*_S_ of approximately 0.9 (Fig. 2a) and peaks at similar *K*_S_ values were identified in other Nymphaeaceae species (Supplementary Note 5.2), which suggests an ancient single WGD event that is probably shared among Nymphaeaceae members. Comparison of the *N. colorata* paralogue *K*_S_ distribution with *K*_S_ distributions of orthologues (representing speciation events) between *N. colorata* and other Nymphaeales lineages, *Illicium henryi*, and *Amborella* suggests that the WGD occurred just after the divergence between Nymphaeaceae and Cabombaceae (Fig. 2a). By contrast, phylogenomic analyses of gene families that contained at least one paralogue pair from collinear regions of *N. colorata* suggest that the WGD is shared between Nymphaeaceae and Cabombaceae (Fig. 2b, Supplementary Note 5.4). If true, *Cabomba caroliniana* seems to have retained few duplicates (Fig. 2b, c), which would explain the absence of a clear peak in the *C. caroliniana* paralogue *K*_S_ distribution (Supplementary Note 5.2). Absolute dating of the paralogues of *N. colorata* does suggest that the WGD could have occurred before or close to the divergence between Nymphaeaceae and Cabombaceae (Extended Data Fig. 2d, Supplementary Note 5.3), considering the variable substitution rates among Nymphaealean lineages (Fig. 2a, b, Extended Data Fig. 2c). An alternative interpretation of the above results could be that the WGD signatures were from an allopolyploidy event that occurred between ancestral Nymphaeaceae and Cabombaceae lineages shortly after their divergence and that gave rise to the Nymphaeaceae (but not Cabombaceae) stem lineage (Fig. 2d, Supplementary Note 5.4).

**Extended Data Fig. 3:**
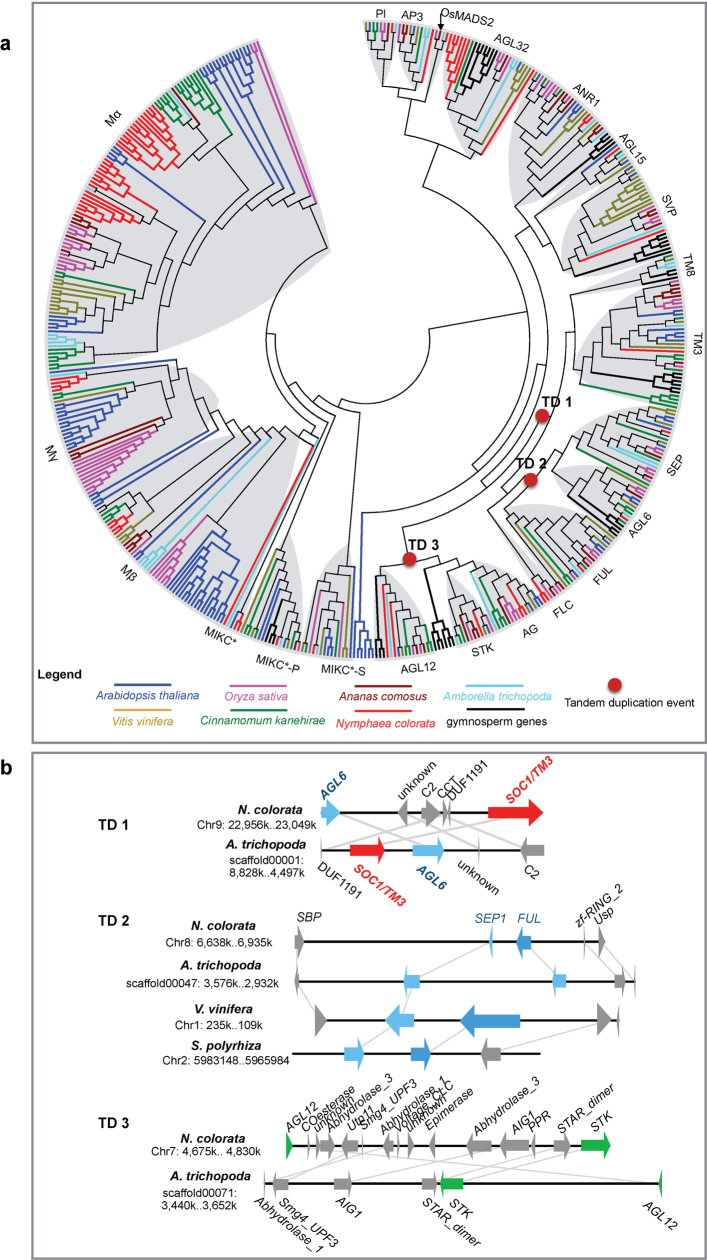
The phylogenetic tree of MADS-box genes of *N. colorata*. **a**, The MADS-box genes are divided into type I and type II, and the latter was subdivided into MIKC^c^ and MIKC*. Branches of various species are shown in different colours, with the colour code below the tree. The nodes representing three tandem duplication events (TD1, TD2, and TD3 in **b**) are marked with red circles. **b**, Genomic regions with the duplicated genes derived from the three tandem duplication events (TD1, TD2 and TD3).

**Extended Data Fig. 4:**
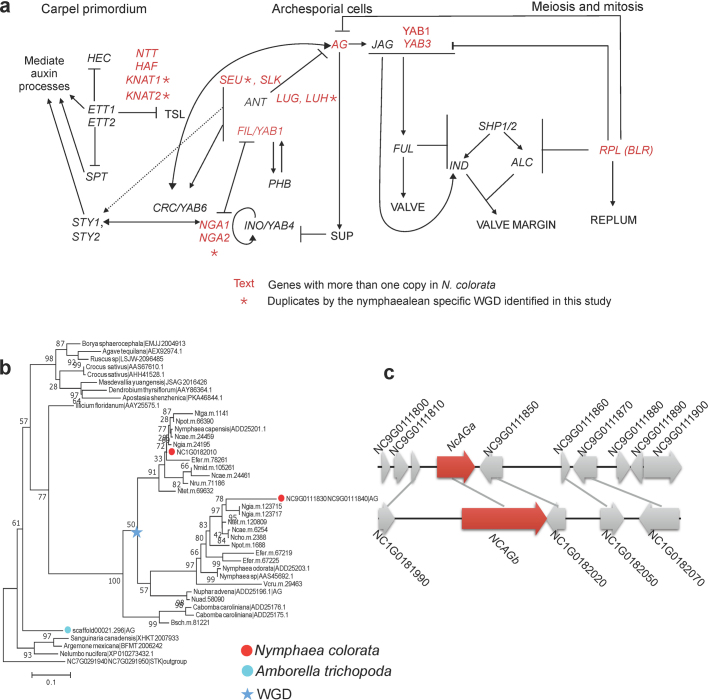
Expansion of key genes regulating the carpel development by the Nymphaealean WGD. **a**, The reported pathway and genes that regulate carpel development. The red-labelled gene has two copies in *N. colorata*. The asterisk indicates that there is collinear support and is retained by the nymphaealean-specific WGD. **b**, Phylogenetic tree of *AG* genes, which specify floral meristem to determine the carpel and stamen identity. The star indicates the WGD specific to the water lily, as detected in this study. The duplicated *AG* genes in *N. colorata* are highlighted in red. **c**, Nc*AG* gene duplicates, Nc*AGa* and Nc*AGb*, are the result of the nymphaealean-specific WGD.

**Extended Data Fig. 5:**
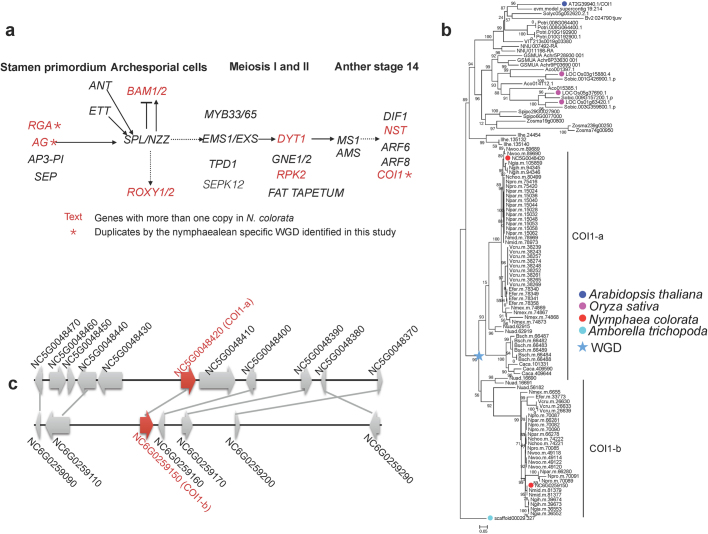
Expansion of key genes regulating the development of the stamen by Nymphaealean WGD. **a**, The reported pathway and genes that regulate the stamen development. The red-labelled gene has two copies in *N. colorata*. The asterisk indicates that there is collinear support and is retained by the nymphaealean-specific WGD. **b**, Phylogenetic tree of *CORONATINE INSENSITIVE 1* (*COI1*), which recruits regulators of pollen development for modification by ubiquitination, needed in the JA response and regulating pollen fertility. **c**, Nc*COI1* gene duplicates have evolved through the WGD in Nymphaeales. The star indicates the nymphaealean-specific WGD.

**Extended Data Fig. 6:**
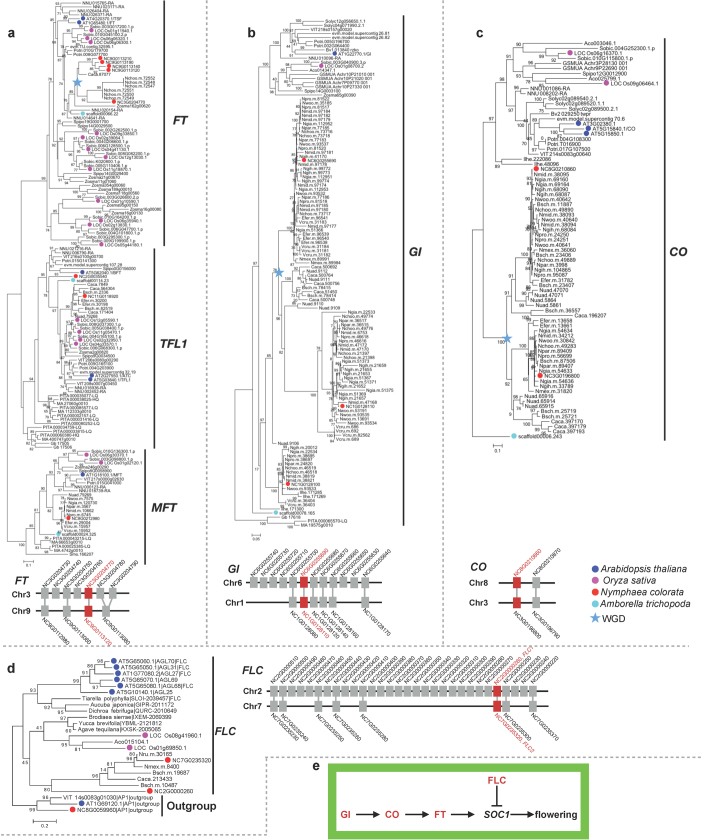
Nymphaealean-specific duplication of the genes that control the initiation of flowering. **a**, Phylogenetic tree of the PEBP-domain containing gene family, including *FT*, *TFL1* and *MFT* subfamilies across various water lily species and other representative seed plants. **b**–**d**, Phylogenetic tree of the *GI* (**b**), *CO* (**c**) and *FLC* (**d**) gene family across various water lily species and other representative seed plants. **e**, The regulatory pathway for the flowering time control. The red-labelled gene has two copies in *N. colorata* and is retained by nymphaealean-specific WGD.

The water lily lineage descended from one of the early divergences among angiosperms, before the radiation of mesangiosperms. Thus, this group offers a unique window into the early evolution of angiosperms, particularly that of the flower. We identified 70 MADS-box genes, including homologues of the genes for the ABCE model of floral organ identities: *AP1* (and also *FUL*) and *AGL6* (A function for sepals and petals), *AP3* and *PI* (B function for petals and stamen), *AG* (C function for stamen and carpel), and *SEP1* (E function for interacting with ABC function proteins). Phylogenetic and collinearity analyses of the MADS-box genes and their genomic neighbourhood indicate that an ancient tandem duplication before the divergence of seed plants gave birth to the ancestors of A function (*FUL*) and E function genes (*SEP*) (Extended Data Fig. 3, Supplementary Note 6.1). Also, owing to the Nymphaealean WGD, *N. colorata* has two paralogues, *AGa* and *AGb* of the C-function gene *AG* (Extended Data Fig. 4). Similarly, the Nymphaealean WGD-derived duplicates are homologous to other genes associated with development of carpel and stamen^8^, and to genes that regulate flowering time^9^ and auxin-controlled circadian opening and closure of the flower^10^ (Extended Data Figs. 4–6, Supplementary Note 6.2–6.4).

**Fig. 3:**
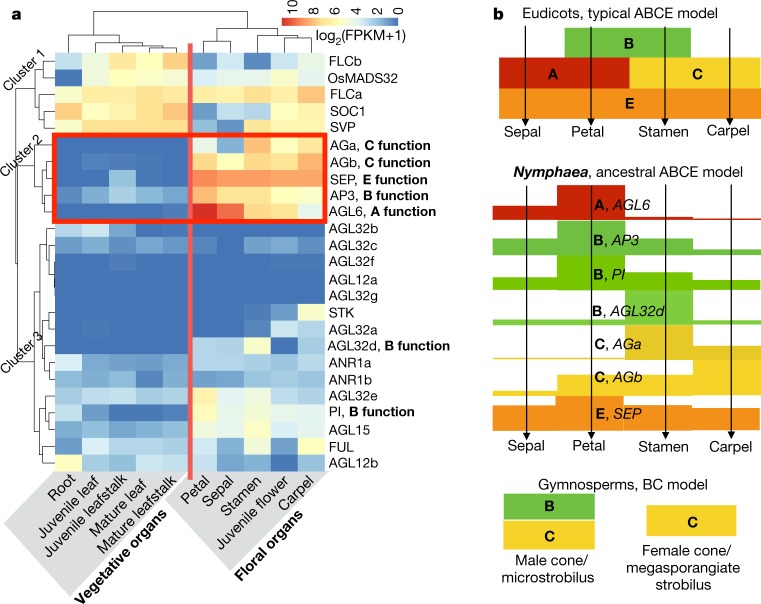
MADS-box genes in *N. colorata* and proposed floral ABCE model in early angiosperms. **a**, Gene expression patterns of MIKC^c^ from various organs of *N. colorata*. Three clusters of genes were classified according to the expression of type II MADS-box genes. The organ types (vegetative organs and floral organs) were matched to the expression patterns of type II MADS-box genes. Expression values were scaled by log_2_(FPKM + 1), in which FPKM is fragments per kilobase of exon per million mapped reads. **b**, The flowering ABCE model in *N. colorata* that specifies floral organs is proposed based on the gene expression values (bar heights) from **a**.

The expression profiles of *N. colorata* ABCE homologues largely agree with their putative ascribed roles in floral organ patterning (Fig. 3a). Notably, the *N. colorata AGL6* homologue is mainly expressed in sepals and petals, whereas the *FUL* homologue is mainly expressed in carpels, suggesting that *AGL6* acts as an A-function gene in *N. colorata*. The two C-function homologues *AGa* and *AGb* are highly expressed in stamens and carpels, respectively, whereas *AGb* is also expressed in sepals and petals, suggesting that they might have undergone subfunctionalization and possibly neofunctionalization for flower development after the Nymphaealean WGD. Furthermore, the ABCE homologues in *N. colorata* generally exhibit wider ranges of expression in floral organs than their counterparts in eudicot model systems (Fig. 3b). This wider expression pattern, in combination with broader expression of at least some ABCE genes in some eudicots representing an early-diverging lineage^11^, some monocots^12^ and magnoliids^13^, suggest an ancient ABCE model for flower development, with subsequent canalization of gene expression and function regulated by the more specialized ABCE genes during the evolution of mesangiosperms, especially core eudicots^8^. This could also account for the limited differentiation between sepals and petals in Nymphaeales species, and is consistent with a single type of perianth organ proposed in an ancestral angiosperm flower^14^.

**Fig. 4:**
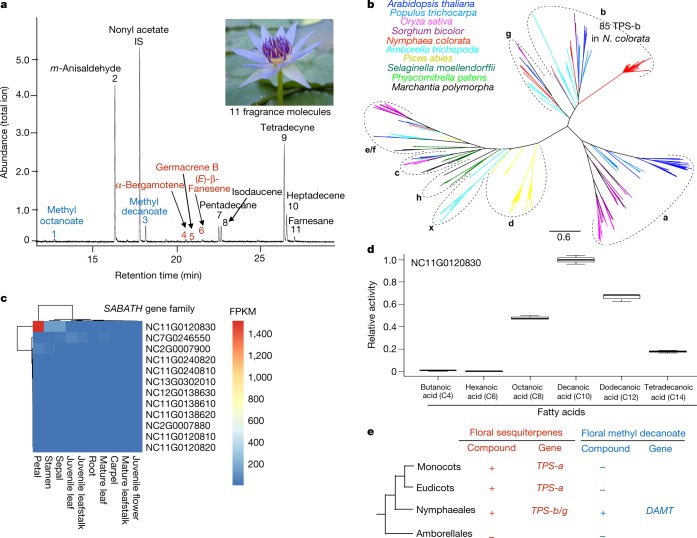
Floral scent and biosynthesis in *N. colorata*. **a**, Gas chromatogram of floral volatiles from the flower of *N. colorata*. The internal standard (IS) is nonyl acetate. Methyl esters are in blue; terpenes are in red. Floral scent was measured three times independently with similar results. **b**, Phylogenetic tree of terpene synthases from *N. colorata* and representative plants showing the subfamilies from a–h and x. **c**, Expression analysis of *SABATH* genes of *N. colorata* showed that NC11G0120830 had the highest expression level in petal.**d**, Relative activity of *Escherichia coli*-expressed NC11G0120830 with six fatty acids as substrates, with the activity on decanoic acid set at 1.0. Data are mean ± s.d. of three independent measurements. **e**, The presence (+) and absence (−) of sesquiterpenes and methyl decanoate as floral scent compounds and their respective biosynthetic genes in four major lineages of angiosperms when known. *DAMT*, decanoic acid methyltranferase.

**Extended Data Fig. 7:**
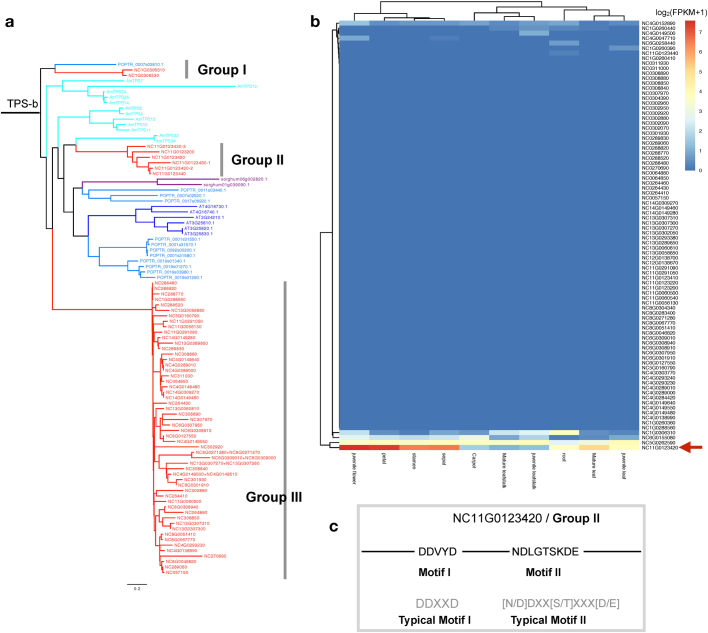
Explosive expansion of the TPS-b subfamily and its implications. **a**, The phylogenetic classification of TPS-b subfamily into three groups. **b**, The group II member NC11G0123420 is the sole gene with high expression in the petal. **c**, Whereas most TPS-b members lack the two typical catalytic motifs, the NC11G0123420 retained both motifs, suggesting its potential role in producing sesquiterpene in *N. colorata*.

Floral scent serves as olfactory cues for insect pollinators^15^. Whereas *Amborella* flowers are scentless^16^, *N. colorata* flowers release 11 different volatile compounds, including terpenoids (sesquiterpenes), fatty-acid derivatives (methyl decanoate) and benzenoids (Fig. 4a). The *N. colorata* genome contains 92 putative terpene synthase (*TPS*) genes, which are ascribed to four previously recognized TPS subfamilies in angiosperms: TPS-b, TPS-c, TPS-e/f and TPS-g (Fig. 4b), but none was found for TPS-a, which is responsible for sesquiterpene biosynthesis in mesangiosperms^17^. Notably, TPS-b contains more than 80 genes in *N. colorata*; NC11G0123420 is highly expressed in flowers (Extended Data Fig. 7); this result suggests that it may be a candidate gene for sesquiterpene biosynthase in *N. colorata*. Also, methyl decanoate has not been detected as a volatile compound in monocots and eudicots^18^ and is thought to be synthesized in *N. colorata* by the SABATH family of methyltransferases^19^. The *N. colorata* genome contains 13 *SABATH* homologues and 12 of them form a Nymphaeales-specific group (Supplementary Fig. 41). Among these 12 members, NC11G0120830 showed the highest expression in petals (Fig. 4c) and its corresponding recombinant protein was demonstrated to be a fatty acid methyltransferase that had the highest activity with decanoic acid as the substrate (Fig. 4d, Supplementary Note 7.1). These results suggest that the floral scent biosynthesis in *N. colorata* has been accomplished through enzymatic functions that have evolved independently from those in mesangiosperms (Fig. 4e).

**Extended Data Fig. 8:**
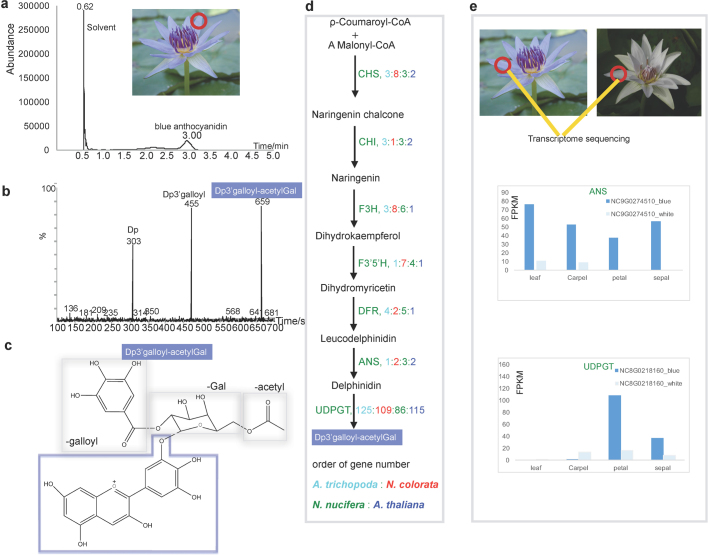
The blue anthocyanidin and its potential biosynthesis pathway in *N. colorata*. **a**, The peak of the blue anthocyanidin appears at 3 min of the high-performance liquid chromatography (HPLC) detection. **b**, The three fragments of the blue anthocyanidin and their molecule mass. **c**, The molecule of the anthocyanidin was identified as delphinidin 3′-*O*-(2″-*O*-galloyl-6″-*O*-acetyl-β-galactopyranoside), abbreviated as Dp3′galloyl-acetylGal. **d**, The postulated pathway for the biosynthesis of Dp3′galloyl-acetylGal. Gene copy numbers are listed next to the enzymes. 3GGT, anthocyanidin 3-*O*-glucoside-2″-*O*-glucosyltransferase; 3′GT, 3′-*O*-beta-glucosyltranferase; 5AT, anthocyanin-5-aromatic acyltransferase; ANS, anthocyanidin synthase; CHI, chalcone isomerase; CHS, chalcone synthase; DFR, dihydroflavonol-4-reductase; F3H, flavanone-3-hydroxylase; F3′5′H, flavonoid-3′,5′-hydroxylase. **e**, Comparative transcriptomic analyses between the blue- and white-petal cultivars of *N. colorata* identified two genes, *ANS* and *UDPGT*, that are highly differentially expressed and might be potential regulators for blue coloration of the petals.

*Nymphaea colorata* is valued for the aesthetically attractive blue colour of petals, which is a rare trait in ornamentals. To understand the molecular basis of the blue colour, we identified delphinidin 3′-*O*-(2″-*O*-galloyl-6″-*O*-acetyl-β-galactopyranoside) as the main blue anthocyanidin pigment (Extended Data Fig. 8a–c). By comparing the expression profiles between two *N. colorata* cultivars with white and blue petals for genes in a reconstructed anthocyanidin biosynthesis pathway, we found genes for an anthocyanidin synthase and a delphinidin-modification enzyme, the expression of which was significantly higher in blue petals than in white petals (Extended Data Fig. 8d, e). These two enzymes catalyse the last two steps of anthocyanidin biosynthesis and are therefore key enzymes specialized in blue pigment biosynthesis^20,21^ (Supplementary Note 7.2).

**Extended Data Fig. 9:**
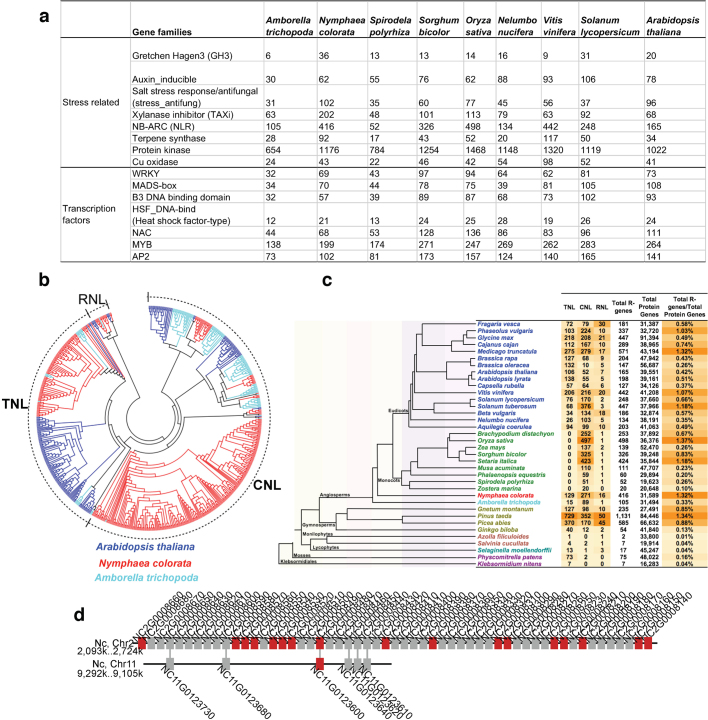
Expanded stress-related and transcription factor gene families in the genome of *N. colorata*. **a**, Markedly expanded gene families for stress response and transcriptional regulation. NLR genes contain NB-ARC domains. Notably, *N. colorata* encodes the highest proportion of kinase genes compared with gymnosperms or other land plants. **b**, NLR genes expanded in all of its three subfamilies (*RNL*, *TNL* and *CNL*). **c**, Distribution of NLR genes across the representative algae and land plants. The background colours indicate the number variation in each species. **d**, An example showing how tandem duplication and WGD contributed to the expansion of R genes in *N. colorata*.

Water lilies have a global distribution that includes cold regions (northern China and northern Canada), unlike the other ANA-grade angiosperms *Amborella* (Pacific Islands) and Austrobaileyales (temperate and tropical regions). We detected marked expansions of genes related to immunity and stress responses in *N. colorata*, including genes encoding nucleotide-binding leucine-rich repeat (NLR) proteins, protein kinases and WRKY transcription factors, compared with those in *Amborella* and some mesangiosperms (Extended Data Fig. 9, Supplementary Note 8). It is possible that increased numbers of these genes enabled water lilies to adapt to various ecological habitats globally.

In conclusion, the *N. colorata* genome offers a reference for comparative genomics and for resolving the deep phylogenetic relationships among the ANA-grade and mesangiosperms. It has also revealed a WGD specific to Nymphaeales, and provides insights into the early evolution of angiosperms on key innovations such as flower development and floral scent and colour.

## Methods

### Genome and transcriptome sequencing

Total DNA for genome sequencing was extracted from young leaves. Leaf RNA was extracted from 18 water lily species: *N. colorata*, *Euryale ferox*, *Brasenia schreberi*, *Victoria cruziana*, *Nymphaea mexicana*, *Nymphaea prolifera*, *Nymphaea tetragona*, *Nymphaea potamophila*, *Nymphaea caerulea*, *Nymphaea rubra*, *Nymphaea* ‘midnight’, *Nymphaea* ‘Choolarp’, *Nymphaea* ‘Paramee’, *Nymphaea* ‘Woods blue goddess’, *Nymphaea gigantea* ‘Albert de Lestang’, *N. gigantea* ‘Hybrid I’, *Nymphaea* ‘Thong Garnjana’ and *Nuphar lutea*. In addition, for transcriptome sequencing we sampled several organs and tissues from *N. colorata* including mature leaf, mature leafstalk, juvenile flower, juvenile leaf, juvenile leafstalk, carpel, stamen, sepal, petal and root.

For PacBio sequencing, we prepared approximately 20-kb SMRTbell libraries. A total of 34 SMRT cells and 49.8 Gb data composed of 5.5 million reads were sequenced on PacBio RSII system with P6-C4 chemistry. All transcriptome libraries were sequenced using the Illumina platform, generating paired-end reads. For the Hi-C sequencing and scaffolding, a Hi-C library was created from tender leaves of *N. colorata*. In brief, the leaves were fixed with formaldehyde and lysed, and the cross-linked DNA was then digested with MboI overnight. Sticky ends were biotinylated and proximity-ligated to form chimeric junctions, which were physically sheared to and enriched for sizes of 500–700 bp. Chimeric fragments representing the original cross-linked long-distance physical interactions were then processed into paired-end sequencing libraries and 346 million 150-bp paired-end reads, which were sequenced on the Illumina platform.

### Sequence assembly and gene annotation

To assemble the 49.8 Gb data composed of 5.5 million reads, we filtered the reads to remove organellar DNA, reads of poor quality or short length, and chimaeras. The contig-level assembly was performed on full PacBio long reads using the Canu package^22^. Canu v.1.3 was used for self-correction and assembly. We then polished the draft assembly using Arrow (https://github.com/PacificBiosciences/GenomicConsensus). To increase the accuracy of the assembly, Illumina short reads were recruited for further polishing with the Pilon program (https://github.com/broadinstitute/pilon). The genome assembly quality was measured using BUSCO (Benchmarking Universal Single-Copy Orthologues)^23^ v.3.0. The paired-end reads from Hi-C were uniquely mapped onto the draft assembly contigs, which were grouped into chromosomes and scaffolded using the software Lachesis (https://github.com/shendurelab/LACHESIS).

Genscan (http://genes.mit.edu/GENSCAN.html) and Augustus^24^ were used to carry out de novo predictions with gene model parameters trained from *Arabidopsis thaliana*. Furthermore, gene models were de novo predicted using MAKER^25^. We then evaluated the genes by comparing MAKER results with the corresponding transcript evidence to select gene models that were the most consistent on the basis of an AED metric.

### The evolutionary position of water lily and divergence-time estimation

LCN genes were identified based on OrthoFinder^26^ results. The orthologues were obtained from six monocots (*Spirodela polyrhiza*, *Zostera marina*, *Musa acuminata*, *Ananas comosus*, *Sorghum bicolor* and *Oryza sativa*) and six eudicots (*Nelumbo nucifera*, *Vitis vinifera*, *Populus trichocarpa*, *A. thaliana*, *Solanum lycopersicum* and *Beta vulgaris*), *N. colorata*, *Amborella*, and the gymnosperms *G. biloba*, *P. abies* and *P. taeda*. LCN genes needed to meet the following requirements: strictly single-copy in *N. colorata*, *Amborella*, *G. biloba*, *P. abies* or *P. taeda*, and single-copy in at least five of the 12 eudicots or monocots. With *G. biloba*, *P. abies* or *P. taeda* as the outgroup, we identified 2,169, 1,535 and 1,515 orthologous LCN genes, respectively. Furthermore, we trimmed the sites with less than 90% coverage. LCN gene trees were estimated from the remaining sites using RAxML v.7.7.8 using the GTR+G+I model for nucleotide sequences (Fig. 1c) and the JTT+G+I model for amino acid sequences (Supplementary Note 4.1). To account for incomplete lineage sorting and different substitution rates, we applied the multispecies coalescent model and a supermatrix method, respectively, to the LCN genes and found further support for the sister relationship between *Amborella* and all other extant flowering plants (Supplementary Note 4.2).

We further carefully selected five LCN gene sets (1,167, 834, 683, 602 and 445) from 115 species and applied both a supermatrix method^27–29^ and the multi-species coalescent model to infer the phylogeny of angiosperms (Supplementary Note 4.2). The phylogeny inferred from 1,167 LCN genes is shown in Fig. 1d, with different support values from the multi-species coalescent analyses of the other four LCN gene sets.

To estimate the evolutionary timescale of angiosperms, we calibrated a relaxed molecular clock using 21 fossil-based age constraints^7^ throughout the tree, including the earliest fossil tricoplate pollen (approximately 125 Ma) associated with eudicots^30^. We concatenated 101 selected genes (205,185 sites) and fixed the tree topology to that inferred from our coalescent-based analysis of 1,167 genes from 115 taxa. We performed a Bayesian phylogenomic dating analysis of the 101 selected genes in MCMCtree, part of the PAML package^31,32^, and used approximate likelihood calculation for the branch lengths^33^. Molecular dating was performed using an auto-correlated model of among-lineage rate variation, the GTR substitution model, and a uniform prior on the relative node times. Posterior distributions of node ages were estimated using Markov chain Monte Carlo sampling, with samples drawn every 250 steps over 10 million steps following a burn-in of 500,000 steps. We checked for convergence by running the analysis in duplicate and checked for sufficient sampling.

We also implemented the penalized likelihood method under a variable substitution rate using TreePL^34^ and r8s^35^, as a constant substitution rate across the phylogenetic tree was rejected (*P* < 0.01) for all cases by likelihood-ratio tests in PAUP^36^. Three fossil calibrations, corresponding to the crown groups of Lamiales, Cornales and Laurales, were implemented as minimum age constraints in our penalized likelihood dating analysis, except that the earliest appearance of tricolpate pollen grains (about 125 Ma)^30^ was used to fix the age of crown eudicots. We determined the best smoothing parameter value of the concatenated 101 LCN genes as 0.32 by performing cross-validations of a range of smooth parameters from 0.01 to 10,000 (algorithm = TN; crossv = yes; cvstart = −2; cvinc = 0.5; cvnum = 15). We used 100 bootstrap trees with branch lengths generated by RAxML^37^ to infer the 95% confidence intervals of age estimates (Supplementary Note 4.2).

### Identification of WGD

The *N. colorata* genome was compared with each of the other genomes by pairwise alignment using Large-Scale Genome Alignment Tool (LAST; http://last.cbrc.jp/). We defined syntenic blocks using LAST hits with a distance cut-off of 20 genes apart from the two retained homologous pairs, in which at least four consecutive retained homologous pairs were required. We then obtained the one-to-one blocks to exclude ancient duplication blocks with QUOTA-ALIGN^38^.

*K*_S_-based paralogue age distributions were constructed as previously described^39^. In brief, the paranome was constructed by performing an all-against-all protein sequence similarity search using BLASTP with an *E*-value cut-off of 10^−10^, after which gene families were built with the mclblastline pipeline (v.10-201) (micans.org/mcl). Each gene family was aligned using MUSCLE (v.3.8.31)^40^, and *K*_S_ estimates for all pairwise comparisons within a gene family were obtained using maximum likelihood in the CODEML program^41^ of the PAML package (v.4.4c)^31^. We then subdivided gene families into subfamilies for which *K*_S_ estimates between members did not exceed a value of 5.

To correct for the redundancy of *K*_S_ values (a gene family of *n* members produces *n*(*n* − 1)/2 pairwise *K*_S_ estimates for *n −* 1 retained duplication events), we inferred a phylogenetic tree for each subfamily using PhyML^42^ with the default settings. For each duplication node in the resulting phylogenetic tree, all *m K*_S_ estimates between the two child clades were added to the *K*_S_ distribution with a weight of 1/*m* (in which *m* is the number of *K*_S_ estimates for a duplication event), so that the weights of all *K*_S_ estimates for a single duplication event summed to one. Paralogous gene pairs found in duplicated collinear segments (anchor pairs) from *N. colorata* were detected using i-ADHoRe (v.3.0) with ‘level_2_only = TRUE’^43,44^. The identified anchor pairs are assumed to correspond to the most recent WGD event.

The *K*_S_-based orthologue age distributions were constructed by identifying one-to-one orthologues between species using InParanoid^45^ with default settings, followed by *K*_S_ estimation using the CODEML program as above. *K*_S_ distributions for one-to-one orthologues between *N. colorata* and each of *V. cruziana*, *N. advena*, *C. caroliniana*, *I. henryi* and *Amborella* were used to compare the relative timing of the WGD in *N. colorata* with speciation events within Nymphaeales. *K*_S_ distributions for one-to-one orthologues between the outgroup species *I. henryi* and each of *N. lutea*, *N. advena*, *N. mexicana*, *Nymphaea* ‘Woods blue goddess’, *N. colorata*, and *C. caroliniana* were used to estimate and compare relative substitution rates among these Nymphaealean species. Additional comparisons using *V. vinifera* and *Amborella* as outgroup species instead of *I. henryi* gave similar results (data not shown).

Absolute dating of the identified WGD event in *N. colorata* was performed as previously described^46^. Briefly, paralogous gene pairs located in duplicated segments (anchor pairs) and duplicated pairs lying under the WGD peak (peak-based duplicates) were collected for phylogenetic dating. We selected anchor pairs and peak-based duplicates present under the *N. colorata* WGD peak and with *K*_S_ values between 0.7 and 1.2 (grey-shaded area in Extended Data Fig. 2b) for absolute dating. For each WGD paralogous pair, an orthogroup was created that included the two paralogues plus several orthologues from other plant species as identified by InParanoid^45^ using a broad taxonomic sampling: one representative orthologue from the order Cucurbitales, two from Rosales, two from Fabales, two from Malpighiales, two from Brassicales, one from Malvales, one from Solanales, two from Poaceae (Poales), one from *A. comosus*^47^ (Bromeliaceae, Poales), one from either *M. acuminata*^48^ (Zingiberales) or *Phoenix dactylifera*^49^ (Arecales), one from the Asparagales (from *Asparagus officinalis*^50^, *Apostasia shenzhenica*^46^, or *Phalaenopsis equestris*^51^), one from the Alismatales (either from *S. polyrhiza*^52^ or *Z. marina*^53^), one from *Amborella*, and one from *G. biloba*^54^. In total, 217 orthogroups based on anchor pairs and 142 orthogroups based on peak-based duplicates were collected.

The node joining the two WGD paralogues of *N. colorata* was then dated using the BEAST v1.7 package^55^ under an uncorrelated relaxed-clock model and an LG+G model with four site-rate categories. A starting tree with branch lengths satisfying all fossil prior constraints was created according to the consensus APG IV phylogeny^1^. Fossil calibrations were implemented using log-normal calibration priors on the following nodes: the node uniting the Malvidae based on the fossil *Dressiantha bicarpellata*^56^ with prior offset = 82.8, mean = 3.8528, and s.d. = 0.5^57^; the node uniting the Fabidae based on the fossil *Paleoclusia chevalieri*^58^ with prior offset = 82.8, mean = 3.9314, and s.d. = 0.5^59^; the node uniting the non-Alismatalean monocots based on fossil *Liliacidites*^60^ with prior offset = 93.0, mean = 3.5458, and s.d. = 0.5^61^; the node uniting the *N. colorata* WGD paralogues with the eudicots and monocots based on the sudden abundant appearance of eudicot tricolpate pollen in the fossil record with prior offset = 124, mean = 4.8143 and s.d. = 0.5^62^; and the root uniting the above clades with *Amborella* and then *G. biloba* with prior offset = 307, mean = 3.8876, and s.d. = 0.5^63^. The offsets of these calibrations represent hard minimum boundaries, and their means represent locations for their respective peak mass probabilities in accordance with previous dating studies of these specific clades^63^ (see Supplementary Note 5.3 for an alternative setting of orthogroups).

A run without data was performed to ensure proper placements of the marginal calibration priors, which do not necessarily correspond to the calibration priors specified above, because they interact with each other and the tree prior^64^. Indeed, a run without data indicated that the distribution of the marginal calibration prior for the root did not correspond to the specified calibration density, so we reduced the mean in the calibration prior of the node combining the *N. colorata* WGD paralogues with the eudicots and monocots with offset = 124, mean = 4.4397, s.d. = 0.5 to locate the marginal calibration prior at 220 Ma^62^.

Markov chain Monte Carlo sampling for each orthogroup was run for 10 million steps, with sampling every 1,000 steps to produce a sample size of 10,000. The resulting trace files were inspected using Tracer v.1.5^55^, with a burn-in of 1,000 samples, to check for convergence and sufficient sampling (minimum effective sample size of 200 for all parameters). In total, 263 orthogroups were accepted, and absolute age estimates of the node uniting the WGD paralogous pairs based on both anchor pairs and peak-based duplicates were grouped into one absolute age distribution, for which kernel density estimation and a bootstrapping procedure were used to find the peak consensus WGD age estimate and its 90% confidence interval boundaries, respectively. More detailed methods have been previously described^39^.

To identify the duplication events that resulted in the 2,648 anchor pairs detected in the genome of *N. colorata*, we performed phylogenomic analyses to determine the timing of the duplication events relative to the lineage divergences in Nymphaeales as described previously^46^. Protein-coding genes from 12 species were used, including eight species from Nymphaeaceae and one species from Cabombaceae in Nymphaeales, one species (*I. henryi*) from Austrobaileyales, plus *Amborella* and *G. biloba*. The phylogeny of the 12 species was obtained from Fig. 1d, and the branch lengths in *K*_S_ units were estimated from 23 LCN genes (selected from the 101 LCN genes used in Fig. 1d, because only 23 are shared across all of the species studied) using PAML^31^ under the free-ratio model. OrthoMCL (v.2.0.9)^65^ was used with default parameters to identify gene families. Then, we removed 907 of the 2,648 anchor pairs with *K*_S_ values greater than five. If the remaining anchor pairs fell into different gene families, thus indicating incorrect assignment of gene families by OrthoMCL, we merged the corresponding gene families and finally obtained 53,243 multi-gene gene families. Next, phylogenetic trees were constructed for a subset of 881 gene families with no more than 200 genes that had at least one pair of anchors and one gene from *G. biloba*. Multiple sequence alignments were produced by MUSCLE (v3.8.31)^40^ and were trimmed by trimAl (v.1.4)^66^ to remove low-quality regions based on a heuristic approach (-automated1).

We then used RAxML (v.8.2.0)^67^ with the GTR+G model to estimate a maximum-likelihood tree, starting with 200 rapid bootstraps followed by maximum-likelihood optimizations on every fifth bootstrap tree. Gene trees were rooted based on genes from *G. biloba* if these formed a monophyletic group in the tree; otherwise, mid-point rooting was applied. The timing of the duplication event for each anchor pair relative to the lineage divergence events was then inferred. In brief, internodes from a gene tree were first mapped to the species phylogeny according to the common ancestor of the genes in the gene tree. Each internode was then classified as a duplication node, a speciation node, or a node that has no paralogues and is inconsistent with divergence in the species phylogeny. The parental node(s) of a duplication node supported by an anchor pair were traced towards the root until reaching a speciation node in the gene tree. The duplication event that resulted in the anchor pair was hence circumscribed between the duplication node as the lower bound and the speciation node as the upper bound on the species tree. If the two nodes were directly connected by a single branch on the species tree, the duplication was thus considered to have occurred on the branch. To reduce biased estimations, we used the bootstrap value on the branch leading to a duplication node as support for a duplication event. In total, 497 anchor pairs in 473 gene families coalesced as duplication events on the species phylogeny, and duplication events from 254 anchor pairs in 246 gene families (or from 380 anchor pairs in 364 gene families) had bootstrap values greater than or equal to 80% (or 50%).

### Floral scent measurement, gene identification, and functional characterization

We collected floral volatiles of *N. colorata* using a dynamic headspace sampling system and analysed them using gas chromatography–mass spectrometry (GC–MS) as previously described^68^. After 2 h of collection from the headspace of detached open flowers of *N. colorata* in a glass chamber (10 cm diameter, 30 cm height), volatiles were eluted from the SuperQ volatile collection trap using 100 µl of methylene chloride containing nonyl acetate as an internal standard. We then analysed samples using an Agilent Intuvo 9000 GC system coupled with an Agilent 7000D Triple Quadrupole mass detector. Separation was performed on an Agilent HP 5 MS capillary column (30 m × 0.25 mm) with helium as carrier gas (flow rate of 1 ml min^−1^). We applied splitless injections of 1 µl samples, injection temperature of 250 °C, an initial oven temperature of 40 °C (3-min hold) and a temperature gradient of 5 °C per min increase from 40 °C to 250 °C. Products were identified using the National Institute of Standards and Technology mass spectral database (https://chemdata.nist.gov).

A full-length cDNA of NC11G0120830 was amplified from the open flowers of *N. colorata* using reverse transcription PCR (RT–PCR), and cloned into pET-32a (MilliporeSigma). After confirmation by sequencing, NC11G0120830 was expressed in *E. coli* strain BL21 (DE3) (Stratagene) and the recombinant protein produced was purified using a modified nickel-nitrilotriacetic acid agarose (Invitrogen) protocol as previously reported^69^. For methyltransferase enzyme assays, we used both radiochemical and non-radiochemical reaction systems. The radiochemical reaction system (50 µl) was composed of 50 mM Tris-HCl, pH 7.8, 1 mM substrate, 1 µl ^14^C-*S*-adenosyl-l-methionine, and 1 µl of purified NC11G0120830. After 30 min of incubation at room temperature, 150 µl of ethyl acetate was added to extract the ^14^C-labelled reaction products. The extracts were counted using a scintillation counter (Beckman Coulter) to measure the activity of NC11G0120830. To determine the chemical identity of the reaction product, we performed non-radiochemical assays in which nonradioactive *S*-adenosyl-l-methionine was used as the methyl donor. The reaction product was collected by headspace solid-phase microextraction and analysed by GC–MS as previously described^70^.

### Reporting summary

Further information on research design is available in the Nature Research Reporting Summary linked to this paper.

## Online content

Any methods, additional references, Nature Research reporting summaries, source data, extended data, supplementary information, acknowledgements, peer review information; details of author contributions and competing interests; and statements of data and code availability are available at 10.1038/s41586-019-1852-5.

### Supplementary information

Supplementary Information Supplementary Notes including Supplementary Materials, Methods and Supplementary Figures 1-48, as well as a series of findings in addition to those shown in the main manuscript.

Reporting Summary

Supplementary Tables This file contains Supplementary Tables 1-21.

## Figures and Tables

**Extended Data Table 1:**
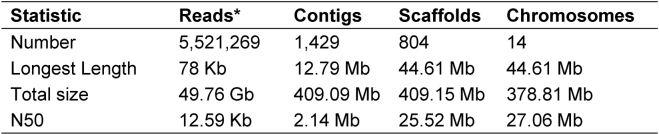
Statistics of the sequenced and assembled genome of *N. colorata*

*The reads only include sequencing by PacBio RS II SMRT sequencing technology.

## Data Availability

PacBio whole-genome sequencing data, Illumina data and genome assembly sequences have been deposited to the NCBI Sequence Read Archive (SRA) as Bioproject PRJNA565347, and were also deposited in the BIG Data Center (http://bigd.big.ac.cn) under project number PRJCA001283. The genome assembly sequences and gene annotations have been deposited in the Genome Warehouse in BIG Data Center under accession number GWHAAYW00000000. The genome assembly sequences, gene annotations, and the LCN genes used in this study, have been also deposited in the Waterlily Pond (http://waterlily.eplant.org). All other data are available from the corresponding author upon reasonable request.
