# Supplementary material for: The water lily genome and the early evolution of flowering plants
Source: Nature. 2019 Dec 18;577(7788):79–84. doi: 10.1038/s41586-019-1852-5 (PMC7015852; doi:10.1038/s41586-019-1852-5)
Supplement: Supplementary file 1 — Supplementary Notes including Supplementary Materials, Methods and Supplementary Figures 1-48, as well as a series of findings in addition to those shown in the main manuscript. [file 41586_2019_1852_MOESM1_ESM.pdf]

nature

https://doi.org/10.1038/s41586-019-1852-5

Supplementary information

The water lily genome and the early evolution of flowering plants

In the format provided by the authors and unedited

Liangsheng Zhang, Fei Chen, Xingtan Zhang, Zhen Li, Yiyong Zhao, Rolf Lohaus, Xiaojun Chang, Wei Dong, Simon Y. W. Ho, Xing Liu, Aixia Song, Junhao Chen, Wenlei Guo, Zhengjia Wang, Yingyu Zhuang, Haifeng Wang, Xuequn Chen, Juan Hu, Yanhui Liu, Yuan Qin, Kai Wang, Shanshan Dong, Yang Liu, Shouzhou Zhang, Xianxian Yu, Qian Wu, Liangsheng Wang, Xueqing Yan, Yuannian Jiao, Hongzhi Kong, Xiaofan Zhou, Cuiwei Yu, Yuchu Chen, Fan Li, Jihua Wang, Wei Chen, Xinlu Chen, Qidong Jia, Chi Zhang, Yifan Jiang, Wanbo Zhang, Guanhua Liu, Jianyu Fu, Feng Chen, Hong Ma, Yves Van de Peer & Haibao Tang

[Supplementary Notes]

The water lily genome and the early evolution of flowering plants

Liangsheng Zhang[1,23]*§, Fei Chen[1,2,23]*, Xingtan Zhang[1,23], Zhen Li[3,4,23], Yiyong Zhao[5,6,23], Rolf Lohaus[3,4,23], Xiaojun Chang[1,7,23], Wei Dong[1], Simon Y. W. Ho[8], Xing Liu[1], Aixia Song[1], Junhao Chen[9], Wenlei Guo[9], Zhengjia Wang[9], Yingyu Zhuang[1], Haifeng Wang[1], Xuequn Chen[1], Juan Hu[1], Yanhui Liu[1], Yuan Qin[1], Kai Wang[1], Shanshan Dong[7], Yang Liu[7, 10], Shouzhou Zhang[7], Xianxian Yu[11], Qian Wu[12,13], Liangsheng Wang[12,13], Xueqing Yan[13,14], Yuannian Jiao[13,14], Hongzhi Kong[13,14], Xiaofan Zhou[15], Cuiwei Yu[16], Yuchu Chen[16] , Fan Li[17], Jihua Wang[17], Wei Chen[18], Xinlu Chen[19], Qidong Jia[20],Chi Zhang[19], Yifan Jiang[2], Wanbo Zhang[2], Guanhua Liu[21], Jianyu Fu[21], Feng Chen[2,19,20]*, Hong Ma[6]*, Yves Van de Peer[3,4,22]*, Haibao Tang[1]*

[1]Fujian Provincial Key Laboratory of Haixia Applied Plant Systems Biology, Key Laboratory of Ministry of Education for Genetics & Breeding and Multiple Utilization of Crops, Key Laboratory of National Forestry and Grassland Administration for Orchid Conservation and Utilization, Fujian Agriculture and Forestry University, Fuzhou 350002, China

[2]College of Horticulture, Nanjing Agricultural University, Nanjing 210095, China

[3]Department of Plant Biotechnology and Bioinformatics, Ghent University, 9052 Ghent, Belgium

[4]VIB Center for Plant Systems Biology, 9052 Ghent, Belgium

[5]State Key Laboratory of Genetic Engineering and Ministry of Education Key Laboratory of Biodiversity Sciences and Ecological Engineering, School of Life Sciences, Fudan University, Shanghai 200433, China

[6]Department of Biology and the Huck Institutes of the Life Sciences, The Pennsylvania State University, University Park, PA 16802, USA

[7]Fairy Lake Botanical Garden, Shenzhen & Chinese Academy of Science, Shenzhen 518004, China

[8]School of Life and Environmental Sciences, University of Sydney, NSW 2006, Australia

[9]State Key Laboratory of Subtropical Silviculture, School of Forestry and Biotechnology, Zhejiang A&F University, Hangzhou 311300, China

[10]BGI-Shenzhen, Shenzhen 518120, China

[11]School of Urban-rural Planning and Landscape Architecture, Xuchang University, Xuchang 461000, China

[12]Key Laboratory of Plant Resources and Beijing Botanical Garden, Institute of Botany, Chinese Academy of Sciences, Beijing 100093, China

[13]University of the Chinese Academy of Sciences, Beijing 100049, China

[14]State Key Laboratory of Systematic and Evolutionary Botany, Institute of Botany, Chinese Academy of Sciences, Beijing 100093, China

[15]Guangdong Province Key Laboratory of Microbial Signals and Disease Control, Integrative Microbiology Research Centre, South China Agricultural University, Guangzhou 510642, China

[16]Hangzhou Tianjing Aquatic Botanical Garden, Zhejiang Humanities Landscape Co., Ltd., Hangzhou 310000, China
[17]National Engineering Research Center for Ornamental Horticulture, Key Laboratory for Flower Breeding of Yunnan Province, Floriculture Research Institute, Yunnan Academy of Agricultural Sciences, Kunming 650200, China
[18]Innovative Institute of Chinese Medicine and Pharmacy, Chengdu University of Traditional Chinese Medicine, Chengdu 611730, China
[19]Department of Plant Sciences, University of Tennessee, Knoxville 37996, USA
[20]Graduate School of Genome Science and Technology, University of Tennessee, Knoxville, TN, 37996, USA
[21]Key Laboratory of Tea Quality and Safety Control, Ministry of Agriculture and Rural Affairs, Tea Research Institute, Chinese Academy of Agricultural Sciences, Hangzhou 310008, China
[22]Department of Biochemistry, Genetics and Microbiology, University of Pretoria, Private bag X20, Pretoria 0028, South Africa
[23]These authors contributed equally to this work.
*These authors jointly supervised this work.
§e-mail:fafuzhang@163.com

Table of Contents

1. Introduction to water lilies 5

1.1 Nymphaeales 5

1.2 *Nymphaea colorata* Peters 7

1.3 The karyotype of *N. colorata* 8

2. Genome sequencing and assembly 10

2.1 Genome size estimation 10

2.2 Genome and transcriptome sequencing 10

2.3 Genome assembly 11

3. Genome annotation 13

3.1 Repetitive elements 13

3.2 microRNAs 16

3.3 Protein-coding genes 16

3.4 Comparison of gene families from water lily, *Amborella*, eudicots, and monocots 17

3.5 Organellar genomes 22

3.6 Transcriptome assembly and expression quantification 24

4. Resolving deep phylogenetic relationships among Amborellales, Nymphaeales, and core angiosperms 26

4.1 Phylogenomic discordance among low-copy nuclear gene trees 26

4.2 Using low-copy nuclear genes to infer the tree and molecular dating of angiosperms 29

4.3 Chloroplast gene-based tree of angiosperms 37

4.4 Mitochondrial gene-based tree of angiosperms 37

5. Whole-genome duplication in the N. colorata genome 39

5.1 Intra- and inter-genomic collinearity analyses 39

5.2 Analyses of K_S distributions 39

5.3 Timing of the WGD in *N. colorata* 41

5.4 Phylogenomic analyses of the WGD 42

5.5 Pre-angiosperm WGD event ... 44

6. Genes related to floral development in N. colorata ... 46

6.1 MADS-box transcription factors ... 46

6.2 Expansion of genes regulating the morphogenesis of male organs and female organs in Nymphaeales ... 52

6.3 Expansion of genes regulating the floral induction network ... 55

6.4 Expansion of auxin-related genes and the regulation of floral opening and closure in *N. colorata* ... 56

7. Floral scent and colour in N. colorata ... 59

7.1 Explosive expansion of the terpene synthase gene family in *N. colorata* ... 59

7.2 Molecular basis of the blue floral pigment in *N. colorata* petals ... 65

8. Genomic basis of stress and immune signalling in N. colorata ... 70

8.1 Expansion of *N. colorata* kinome ... 70

8.2 R genes in the *N. colorata* genome ... 72

8.3 Anti-fungal domain-containing genes in *N. colorata* ... 72

8.4 Expansion of the WRKY gene family in *N. colorata* ... 73

References ... 75

Supplementary Table information ... 81

1. Introduction to water lilies

1.1 Nymphaeales

The water lily order Nymphaeales is divided into three families Nymphaeaceae, Cabombaceae, and Hydatellaceae[1,2]. Currently, Nymphaeales consists of more than 70 species in eight genera (*Trithuria*, *Cabomba*, *Brasenia*, *Barclaya*, *Euryale*, *Nuphar*, *Victoria*, and *Nymphaea*), grouped into three families: ~56 species in Nymphaeaceae, six species in Cabombaceae, and 12 species in Hydatellaceae are listed on The Plant List (www.theplantlist.org, accessed 30 June 2018). All Nymphaeales species are aquatic herbs and most of the species, except Hydatellaceae and *Cabomba* spp., have rhizomes and broad leaves, and many have showy flowers.

Although the exact number of species in the Nymphaeaceae family is unclear, Christenhusz and Bying (2016) estimated approximately 70 species[3]. Among the three Nymphaealean families, the Nymphaeaceae family contains the greatest number of species as well as most of the economically important species. Nymphaeaceae water lilies are distributed in tropical, temperate, and cold regions. Besides the debated relationship with Amborellales, most recent studies have focused on the phylogeny of the genera within the order of Nymphaeales. Despite this attention, the phylogenetic relationships among the five subgenera within *Nymphaea* (*Lotos*, *Hydrocallis*, *Nymphaea*, *Anecphya*, and *Brachyceras*), as well as the two genera *Victoria* and *Euryale*, remain largely unclear[4,5].

The characteristics that distinguish angiosperms from gymnosperms include (i) the presence of flowers, (ii) an endosperm produced by double fertilization, and (iii) ovules enclosed in the carpel. Investigating the flower-related genes in water lilies should therefore shed light on the origin and early evolution of angiosperms. A morphological comparison of floral organs within different angiosperm clades is included in Supplementary Fig. 1. In gymnosperms, strobils have neither special scent nor colour, and the gymnosperm cone is unisexual. The *Amborella* flower is simple and can be either male or female, with sepals or sepal-like perianth organs that lack floral scent; it also lacks modifications or additional floral features as seen in crown angiosperms. Water lily and *Illicium* flowers have diverse floral scents and colours and contain both male and female organs. Mesangiosperms evolved more features of the flowers: they can be either fragrant or unscented, colourful or white, and unisexual or bisexual. Mesangiosperms also possess various floral modifications such as spots, trichomes, and nectaries.

a

Species	Floral / strobile scent	Petal / strobile specific color	Cone / Flower sex	Modifications on the flower
Ginkgo biloba	NO	NO	Female / Male	-
Amborella trichopoda	NO	NO	Female / Male	NO
Nymphaea colorata	fragrant	Blue	Female & Male	NO
Illicium henryi	fragrant	Red	Female & Male	NO
**Mesangiosperms**	fragrant or not	colorful or colorless	Female, Male, both	spots, trichome, nectaries, etc.

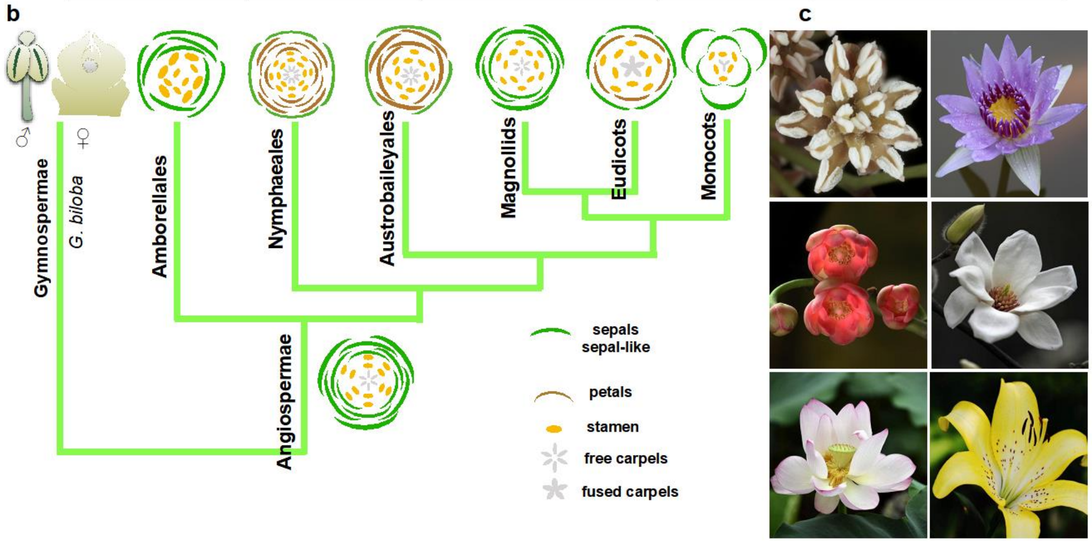

Supplementary Fig. 1 | Morphological comparison of floral organs from different angiosperm clades. a, Comparison of floral organs in *Ginkgo biloba*, *Amborella trichopoda*, *Nymphaea colorata*, *Illicium henryi*, and mesangiosperms. **b**, Comparison of floral diagrams for gymnosperms, Amborella, Nymphaeales, Austrobaileyales, magnoliids, eudicots, and monocots. **c**, Flowers of *Amborella*, *N. colorata*, *I. henryi*, *Magnolia denudata*, *Nelumbo nucifera*, and *Lilium brouwnii*. The last common ancestor of all angiosperms may have possessed bisexual flowers[6].

The Amborellales, Nymphaeales and Austrobaileyales have different ecological niches and species numbers (Supplementary Fig. 2). The order Amborellales has only one extant species, *Amborella trichopoda,* which occupies a small ecological niche only found on the tropical island of New Caledonia. All species belonging to the order Austrobaileyales (~100 species divided into five genera and three families) are found in tropical or subtropical regions. Among the ~90 species in Nymphaeales, Hydatellaceae species are found only in Australia, New Zealand, and India, while the species in the other two families, particularly Nymphaeaceae, have a global distribution that extends from tropical regions to the cool northern parts of Canada[7], with some species (*Nuphar luteum*, *Nymphaea mexicana*, *Nymphaea odorata*, and *Nymphaea* spp.) being invasive and difficult to control (http://iwgs.org/invasive-species/, accessed 30 June 2018).

Order	Environmental adaptations		Number of genera and species		
	**Geological distributions**	**Climate**	**Families**	**Genera**	**Species**
**Austrobaileyales**	**Austrobaileyaceae**: Australia<br>**Illiciaceae**: South East Asia to W. Malesia, S.E. U.S.A., E. Mexico, Greater Antilles, Sri Lanka, East Asia to W. Malesia, S.E. U.S.A., Mexico<br>**Trimeniaceae**: New Guinea and S.E. Australia to Fiji	Tropical, subtropical, cool	3	5	~100
**Nymphaeales**	**Hydatellaceae**: India, New Zealand and Australia<br>**Cabombaceae**: World-wide, rather scattered, from tropical to cold regions<br>**Nymphaeaceae**: World-wide, from tropical to cold regions	Tropical, subtropical, cool, cold	3	8	~90
**Amborellales**	New Caledonia island	Tropical	1	1	1

Supplementary Fig. 2 | Comparison of Amborellales, Nymphaeales, and Austrobaileyales with different environmental adaptations (including global distributions and climate adaptations) and the corresponding numbers of genera and species. The global distribution data of each order were adapted from https://www.mobot.org/MOBOT/research/APweb/ by merging the distribution data of each family. The Austrobaileyales species number was retrieved from MOBOT (https://www.mobot.org/MOBOT/research/APweb, accessed 30 June 2018).

1.1 *Nymphaea colorata* Peters

The common names of the water lily *Nymphaea colorata* are 'blue pigmy' and 'colorata'. It is native to tropical East Africa and was introduced into Asia, Europe, and America for breeding purposes due to its high ornamental value. A single *N. colorata* flower consists of four sepals, ~13 petals, ~72 stamens, ~24 carpels, and thousands of seeds (Supplementary Fig. 3). *N. colorata* is nonviviparous, suitable for small- or medium-sized water gardens, and continuously flowering when the temperature drops to 18 °C. The plant is relatively small, with a ~10 cm leaf diameter that adapts to small spaces, and green on top with a bluish violet color on the underside of the leaf. The flowers are medium in size (8-12 cm), cup-like, violet-blue, paler at the base of the petals and stamens, and mildly fragrant. These features have contributed to the growing popularity of *N. colorata* as an ornamental flower, and it is widely cultivated in aquatic gardens. *N. colorata* has also been incorporated into breeding programs around the world.

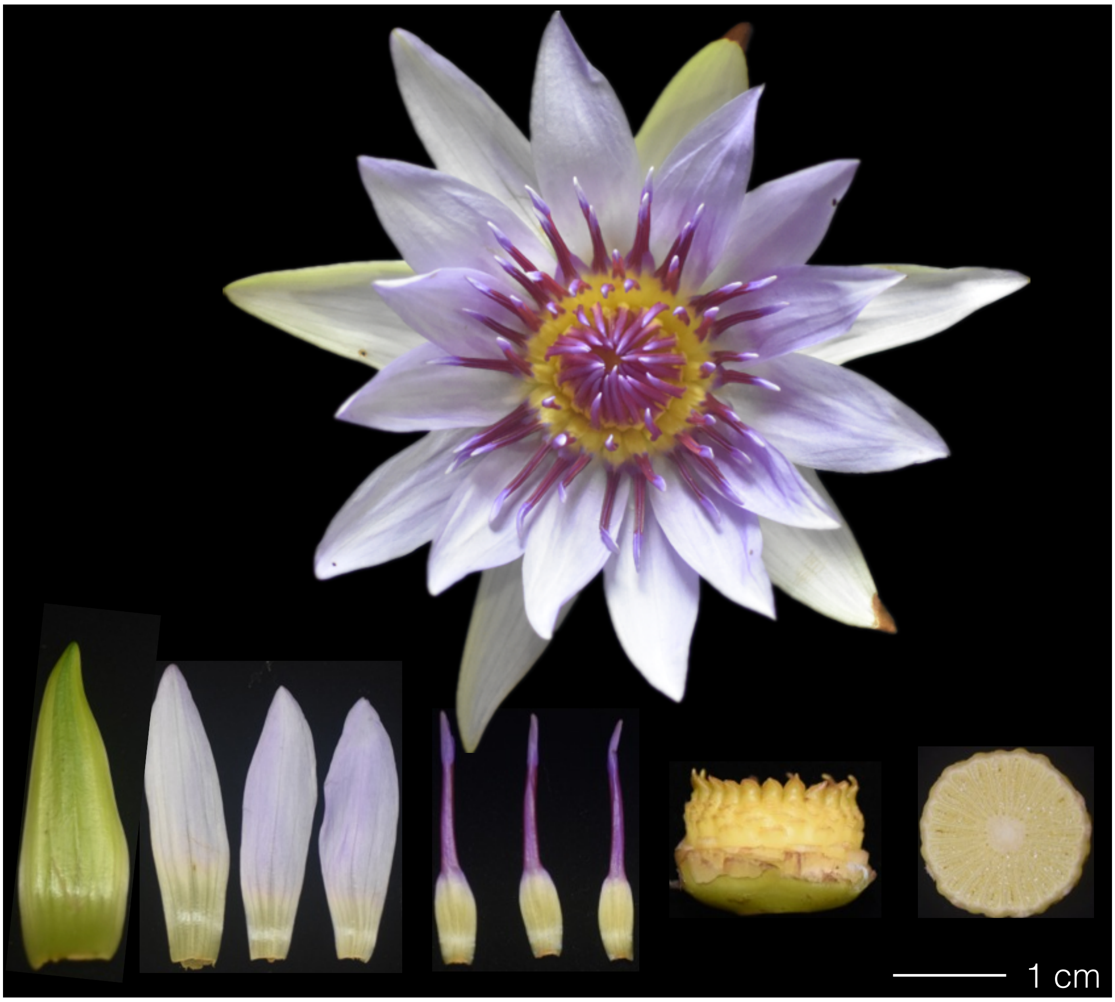

Supplementary Fig. 3 | Floral organs of the water lily *Nymphaea colorata.* A single flower produces hundreds of seeds. The flower is blue but appears slightly purple in the image.

This species has considerable potential as a model plant for studying the Amborellales, Nymphaeales, and Austrobaileyales (ANA)-grade of angiosperms, in part because of its rapid growth rate (three months from seed to seed), and thousands of seeds per fruit[7]. It is also popular in breeding programs for producing water lilies with blue petals. In particular, its beautiful blue petals represent an economically important trait such that its gene(s) have been introduced into other cultivars. For example, *N. colorata* is one of the parents for the following cultivars: *N.* 'Kew's Kabuki', *N.* 'Suwannata', *N.* 'Woods Blue Goddess', *N.* 'Patricia', *N.* 'Midnight', *N.* 'American Beauty', *N.* 'Aquarius', *N.* 'Director George T. Moore', *N.* 'King of Siam' (www.internationalwaterlilycollection.com), and *N.* 'William Phillips[8].

1.3 The karyotype of *N. colorata*

The young roots of *N. colorata* were sampled for DNA karyotyping. Chromosome spreads at meiotic stages from root tissue were prepared as previously described[9]. The chromosome number of *N. colorata* was photographed under an Olympus BX63 fluorescence microscope at Fujian Agriculture and Forestry University in China. Across more than 30 different cells, 28 chromosomes were consistently identified, and four representative cells are shown in

Supplementary Fig. 4. This result was consistent with a previous report on chromosome counting of *Nymphaea*[9] (Supplementary Table 1).

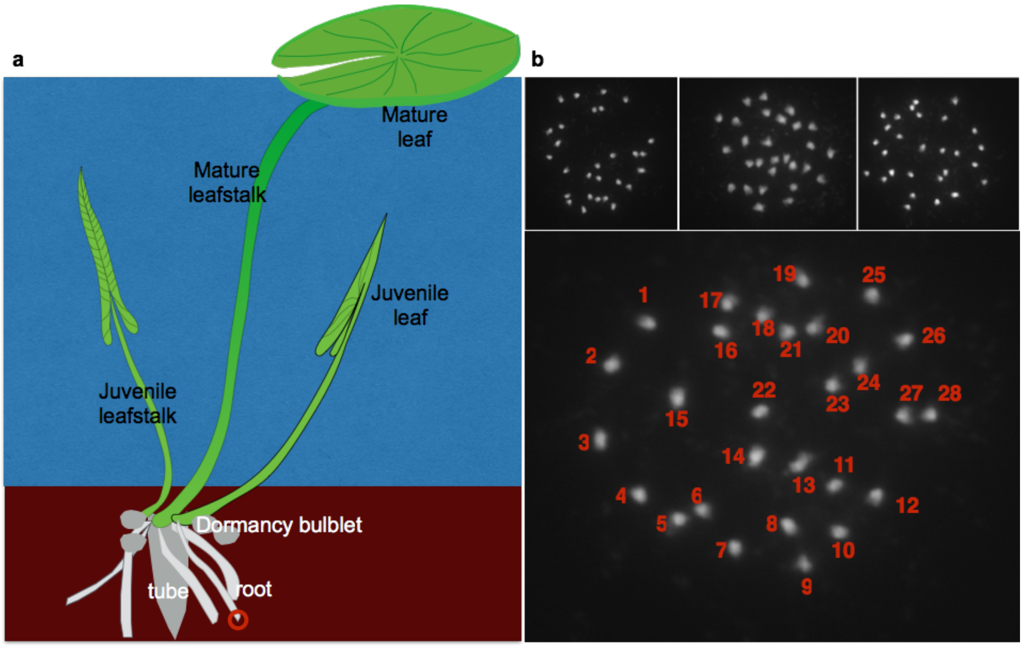

Supplementary Fig. 4 | Four different cells from *Nymphaea colorata* root tips each had 28 chromosomes. a, Schematic drawing showing the tube and root. The red circle indicates the sampled root tissue for chromosome counting. Note that the root system of *N. colorata* includes the roots, dormancy bulblet, and the tube. **b**, Four representative root cells, out of 30 observed samples, at the meiosis stage. Each shows 28 chromosomes.

2. Genome sequencing and assembly

2.1 Genome size estimation

The *N. colorata* genome size was estimated through two methods: (i) flow cytometry to estimate the draft genome size and (ii) *k*-mer based estimation. In the flow cytometry estimation, we compared the genome size of *N. colorata* with that of *Pyrus bretschneideri* ($2n = 34$, 527 Mb[10]) and Indian red water lily *N. rubra* ($2n = 112$)[9]. The genome size of *N. colorata* was estimated as ~400 Mb (Supplementary Fig. 5), a suitable size for genome sequencing. The second analysis indicated a genome size of 461 Mb based on the *k*-mer spectrum derived from sequencing data (Supplementary Table 2). In this analysis, genome size = k_{num}/k_{depth}, where k_{num} is the total number of *k*-mers, and k_{depth} is the expected depth of *k*-mers.

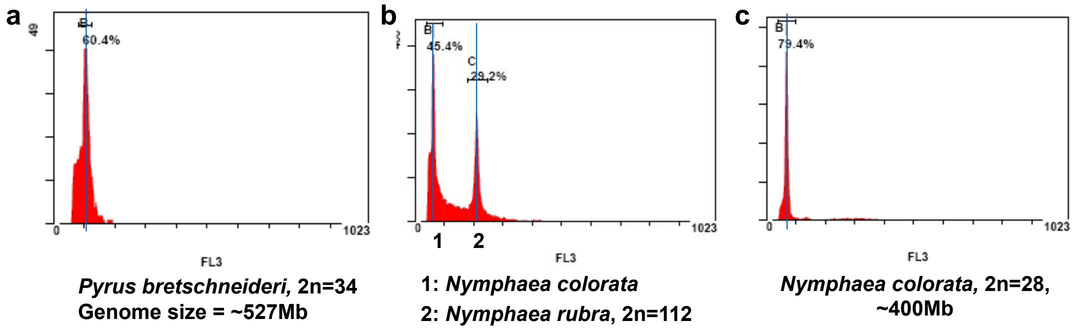

Supplementary Fig. 5 | Genome size estimation of *Nymphaea colorata* based on three flow cytometry studies. a, Pear (*Pyrus bretschneideri*) has a diploid genome with 34 chromosomes and a total size of 527 Mb[10]. **b**, Comparison between *N. colorata* and *N. rubra*, which has a diploid genome with 112 chromosomes[9]. **c**, Based on the flow cytometry estimation and comparison with *N. rubra* and *P. bretschneideri*, the genome size of *N. colorata* was estimated to be ~400 Mb. Three repeats were performed and similar results were obtained.

2.2 Genome and transcriptome sequencing

Materials and Methods

Total DNA for genome sequencing was extracted from young leaves. Total RNA was extracted from leaves for the following 18 Nymphaeales species: *N. colorata*, *N. mexicana, N. prolifera, N. tetragona*, *N. potamophila*, *N. rubra*, *N. caerulea*, *N.* 'midnight', *N.* 'Choolarp', *N.* 'Paramee', *N.* 'Woods blue goddess', *N. gigantea* 'Albert de Lestang', *N. gigantea* 'Hybrid I', *N.* 'Thong Garnjana', *Victoria cruziana*, *Euryale ferox*, *Nuphar lutea*, and *Brasenia schreberi.* In addition, various *N. colorata* organs were sampled for transcriptome sequencing, including mature leaf, mature leafstalk, juvenile flower, juvenile leaf, juvenile leafstalk, carpel, stamen, sepal, petal, and root (Supplementary Table 1). For PacBio RS II genome sequencing, 20 kb single-molecule real-time SMRTbell™ libraries were prepared.

Hi-C refers to high-throughput chromosome conformation capture, which investigates the relationship between interacting chromatin DNA regions resulting from their spatial structure inside the nucleus. For Hi-C sequencing and scaffolding, a Hi-C library was generated from the

tender leaves of water lily. Briefly, the leaves were fixed with formaldehyde and lysed, then the cross-linked DNA was digested with MboI overnight. Sticky ends were biotinylated and proximity-ligated to form chimaeric junctions, which were enriched then physically sheared to a size of 500-700 bp. Chimaeric fragments representing the original cross-linked long-distance physical interactions were processed into paired-end sequencing libraries for sequencing using the illumina HiSeq X Ten platform. We used the HiC-Pro pipeline to map reads, detect ligation products, perform quality controls, and generate intra- and inter-chromosomal contact maps[11].

Results and Discussion

Thirty-four SMRT cells with 49.8 Gb data composed of 5.5 million reads were sequenced using the PacBio RS II system with P6-C4 chemistry. The sequencing reads had an average length of 9,088 bp, with the longest read length of 78,559 bp. Considering the ~400 Mb size of the *N. colorata* genome, these raw sequencing data yielded 124· coverage of the total genome. We obtained 346 million 150 bp paired-end illumina reads from Hi-C sequencing.

2.3 Genome assembly

To assemble the 49.8 Gb data corresponding to 5.5 million reads, we filtered and removed: organellar DNAs, reads of poor quality or short length, and chimaeras. The contig-level assembly was performed on full set of PacBio long reads using the CANU package[12], which is a successor of the Celera Assembler with increased assembly continuity and decreased running time. CANU version 1.3 was used for self-correction and assembly with parameters corOutCoverage=100, ovbMemory=8g, maxMemory=500g, maxThreads=48, ovsMemory=8-500g, ovsThreads=4, and oveMemory=32g on a SGE grid. The draft assembly was polished using Arrow (https://github.com/PacificBiosciences/GenomicConsensus). To increase the consensus accuracy of assembly, illumina short reads were recruited for further polishing with the Pilon program (https://github.com/nanoporetech/ont-assembly-polish). The genome heterozygosity was estimated by mapping the genome sequencing reads to the 409 Mb genome by bwa (https://github.com/lh3/bwa), followed by SNP calling using samtools (https://github.com/samtools). The heterozygosity rate estimated by bcftools (https://github.com/samtools/bcftools) was 0.39%. The heterozygosity estimate using the GCE tool (ftp://ftp.genomics.org.cn/pub/gce/) was 0.23%.

The paired-end Hi-C reads were mapped onto the draft assembly contigs, retaining only uniquely mapped reads. The contigs were grouped into chromosomes based on the Hi-C links, and scaffolded using Lachesis (https://github.com/shendurelab/LACHESIS) with tuned parameters. The interaction matrix file was exported to plot the heatmap. To display each chromosome with detailed contig interactions, we plotted the matrix heatmap using HiCPlotter[13].

Results and Discussion

We obtained a total of 49.8 Gb genomic sequences, yielding a reference genome sequence of 409 Mb. After error correction using illumina reads, the consensus error rate was estimated at 0.08%, with the genomic heterozygosity estimated at 0.39% (Supplementary Table 3). We were able to map 98.85% of 22 Gb illumina sequencing data and 98.52% of transcripts assembled from a leaf transcriptome onto the assembled genome of *N. colorata* (Supplementary Table 4). The genome assembly quality was measured using BUSCO (Benchmarking Universal Single-Copy Orthologs)[14] version 3.0 (https://busco.ezlab.org/frame_plants.html). The latest all-plant gene set, Embryophyta odb10, was used as the reference, and the model species *Arabidopsis* was used in the –sp option. The final genome completeness determined by BUSCO was 94.4% (Supplementary Table 4).

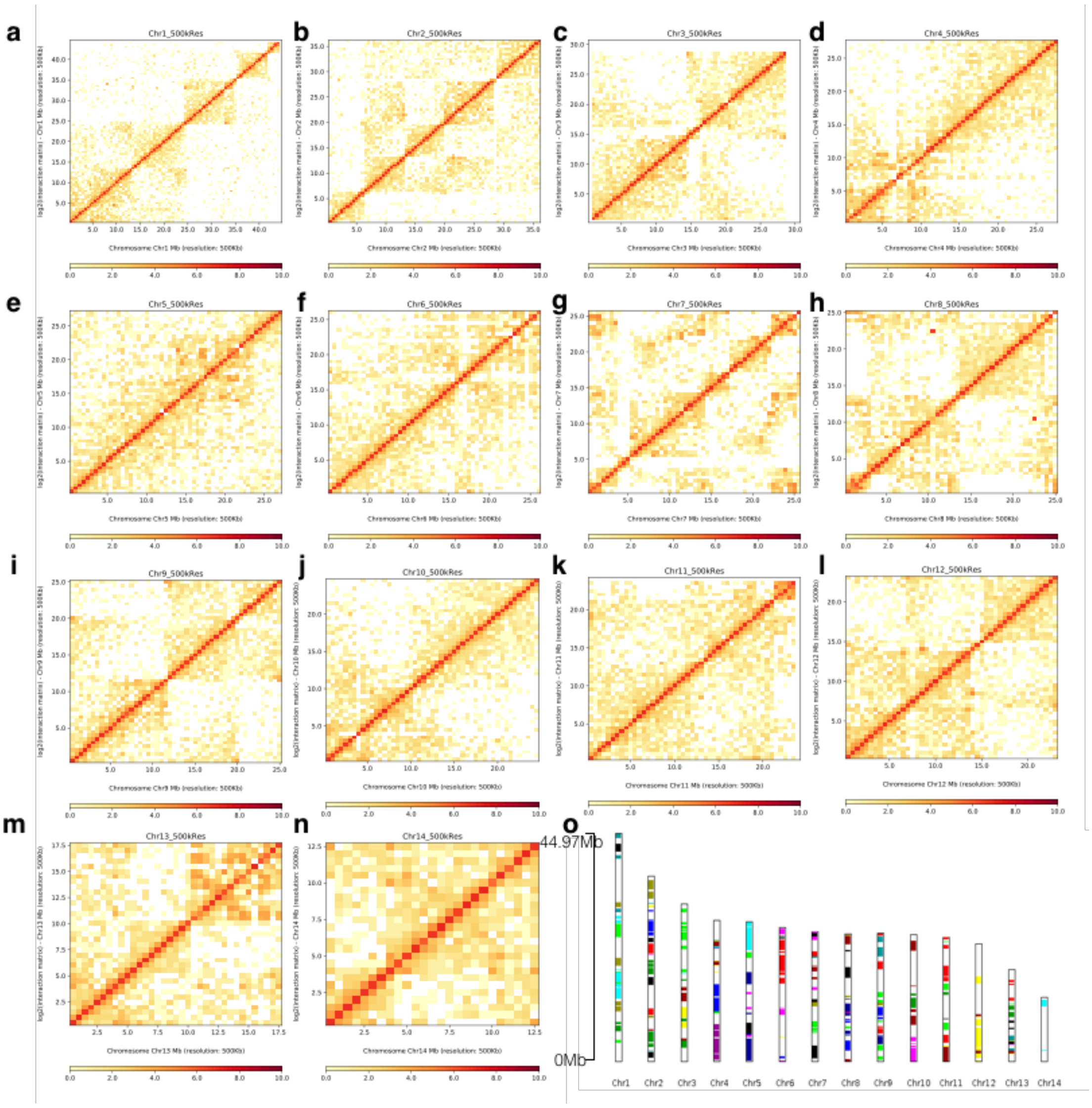

Supplementary Fig. 6 | Contact maps of the 14 chromosomes using 500 kb fragments. a-n, Normalized Hi-C contact matrix and the assembled chromosomes from no. 1 to no. 14. **o**, chromosome level synteny among 14 chromosomes. The same colour refers to within-genome collinearity or synteny.

The contig N50 of *N. colorata* was 2.1 Mb, which is a significant improvement over *Amborella*[15], with a contig N50 of 29 kb (Supplementary Table 6). Based on the 1,429 contigs assembled on PacBio reads, our Hi-C scaffolding procedure anchored a total of 804 contigs onto the 14 pseudo-chromosomes (herein referred to as chromosomes) (Supplementary Fig. 6) The length and number of contigs for each chromosome are shown in Supplementary Table 5. These 14 chromosomes had a total length of 378,814,058 bp, accounting for 85.3% of the 409 Mb genome (Supplementary Table 5). The longest chromosome was 44,612,865 bp. The final scaffold N50 reached 27,058,147 bp.

Assembly statistics for the genomes of *N. colorata* and other representative flowering plants are shown in Supplementary Table 6. The high-quality assembly of the *N. colorata* genome is suitable for annotating genes for in-depth study as well as gene synteny analysis.

3. Genome annotation

3.1 Repetitive elements

Materials and Methods

We constructed *de novo* repeat libraries using RepeatModeler (http://www.repeatmasker.org/RepeatModeler/), which is implemented by two *de novo* repeat-finding programs, RECON[16] and RepeatScout[17], to identify repeat elements and their family relationships. To predict species-specific transposable element (TE) sequences in *N. colorata*, the custom repeat libraries were initially imported into RepeatMasker[18] (http://www.repeatmasker.org) for the identification of TE families. The unknown TE sequences were classified using TEclass[18]. In addition, tandem repeats were identified by TRF[19], and LTRs were detected using LTR_finder[20]. A custom PERL script was used to build a comprehensive TE library in *N. colorata*. Telomeres are critical for chromosome maintenance and for controlling the life span of a cell. Telomeric repeats are tandem and short GC-rich sequences with hundreds of repetitive units. Subtelomeric sequences are immediately proximal to telomeric repeats and are a complex patchwork of low-copy repeat sequences, segmental duplications, and degenerate telomeric repeats[21]. The telomere repeats were identified using an approach outlined in the analysis of the grass species *Oropetium thomaeum*[22]. The centromeric sequences were predicted as described previously[23].

Results and Discussion

Repetitive elements were predicted from representative angiosperms (*N. colorata*, *Amborella trichopoda*, *Vitis vinifera*, *Carica papaya*, *Arabidopsis thaliana*, *Oryza sativa* japonica, *Nelumbo nucifera*, *Solanum lycopersicum*, *Sorghum bicolor*, *Zostera marina*, and *Spirodela polyrhiza*) and classified as class I retroelements, class II DNA transposons, and tandem repeats (Supplementary Table 7).

The cumulative size of predicted repetitive elements in *N. colorata* amounts to 160.4 Mb, accounting for 39.2% of the sequenced genome. Comparison of the repetitive elements of *N. colorata* with those from *Arabidopsis thaliana, Vitis vinifera,* sacred lotus (*Nelumbo nucifera*), beet (*Beta vulgaris*), tomato (*Solanum lycopersicum*), papaya (*Carica papaya*), *Zostera marina*,

Spirodela polyrhiza, *Sorghum bicolor*, *Oryza sativa*, and *Amborella* shows that *N. colorata* contains more copies of the retrotransposon *Copia* (20.27%) than *Gypsy* (12.52%), while the other angiosperm species we investigated have fewer *Copia* than *Gypsy* elements (Supplementary Table 7). This could be partly explained by smaller numbers of the *Gypsy* transposase in *N. colorata* (Supplementary Fig. 7). *Nymphaea colorata*, as well as *Nelumbo nucifera* and *Spirodela polyrhiza*, show an absence of the Tc1/Mariner [DTT] type DNA transposons, which could be found in *Amborella* and in other monocots and eudicots.

For the class I retroelement (Supplementary Table 7), *N. colorata* encoded a larger number and longer total length of *Copia* versus *Gypsy* elements, which contrasts with the pattern in other flowering plants such as *Amborella trichopoda*, *Vitis vinifera*, *Arabidopsis thaliana*, and *Oryza sativa* japonica, suggesting either dramatic expansion of *Copia* or dramatic reduction of *Gypsy*. Besides LTR and non-LTR retrotransposons, *N. colorata* and other flowering plants (*V. vinifera*, *A. thaliana*, and *O. sativa* japonica) contained unclassified retroelements that were not found in the *Amborella trichopoda* genome.

We showed that there were fewer *Gypsy* TEs than *Copia* TEs in *N. colorata*. To investigate this unusual scenario, we constructed phylogenetic trees for the *Copia* and *Gypsy* transposase families in flowering plants, to reveal their distinct evolutionary patterns. Based on the split events between the groups at least occurred in the last common ancestor of angiosperms, the *Gypsy* transposases were divided into eight groups, whereas *Copia* transposases were divided into 14 groups (Supplementary Fig. 7). In *N. colorata*, the number of *Gypsy* transposases was much lower than the number of *Copia* transposases. Considering that transposase can bind to a *Gypsy* sequence and catalyse its movement to another location in the genome through a cut-and-paste mechanism, we propose that the lower number of *Gypsy* transposases in *N. colorata* may account for the lower number of *Gypsy* TEs in the genome.

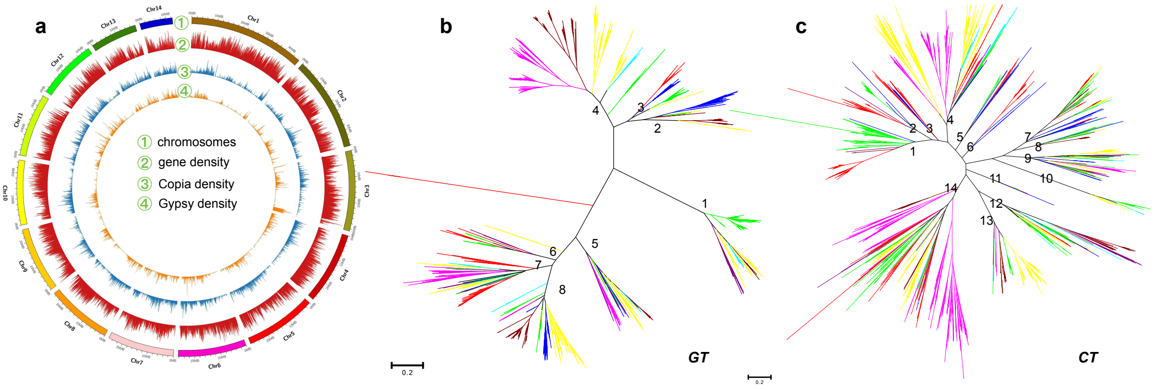

d

Species	*Gypsy* transposase (GT)	*Copia* transposase (CT)	*GT/CT*	*Gypsy*	*Copia*	*Gypsy/Copia*
Arabidopsis thaliana	67	88	0.761363636	5,852	2,141	2.733302195
Vitis vinifera	573	1,762	0.325198638	74,092	66,217	1.118927164
Beta vulgaris	829	1,603	0.517155334	61,706	44,258	1.394233811
Oryza sativa	573	316	1.813291139	50,003	14,032	3.563497719
Spirodela ppolyrhiza	207	207	1	8,323	7,514	1.107665691
Zostera marina	546	1,014	0.538461538	29,267	22,620	1.293854996
Nymphaea colorata	277	1,481	0.187035787	20,874	41,205	0.506589006
Amborella	616	1,512	0.407407407	82,909	56,477	1.468013528

Supplementary Fig. 7 | Evolution of long terminal repeat (LTR) transposase genes in *Nymphaea colorata*. **a**, Chromosomal distribution of *Gypsy* and *Copia* showing denser distributions of *Copia* TEs than *Gypsy* TEs. **b**, Phylogenetic relationships of *Gypsy* transposase genes in representative flowering

plants. **c**, Phylogenetic relationships of *Copia* transposase genes in representative flowering plants. **d**, Statistics for *Gypsy* and *Copia* TEs and their transposase genes in representative flowering plants.

For class II DNA transposons (Supplementary Table 7), Tc1/Mariner [DTT] transposons were not found in the *N. colorata* genome. Since they are present in the genome of moss *Physcomitrella patens*[23], it is possible that Tc1/Mariner transposons were completely lost in the *N. colorata* genome. The rice genome contains 66,350 Tc1/Mariner transposons with a total length of 10.8 Mb, suggesting their unique roles in rice evolution. In contrast, they do not seem to be involved in the genomes of *N. colorata* and *Amborella*.

In addition, a total of 19 telomeres and subtelomeres were identified in the *N. colorata* genome (Supplementary Table 8). Among flowering plants, "TTTAGGG" telomere repeats have been reported in monocots (wheat, barley, and rice) and eudicots (tomato and *Arabidopsis*); they also occur in gymnosperms (*Ginkgo biloba* and *Pinus taeda*)[24]. Here, 19 telomeres were identified in *N. colorata* from nine chromosomes and unanchored scaffolds (Supplementary Table 8). Chr1, Chr2, Chr5, and Chr11 had two or three telomeres, suggesting that some are sub-telomeres located within but not at the end of the chromosome. The copy numbers of telomere repeats ranged from 89 to 1,779, with an average length of 889 bp and median length of 8,060 bp. These putative telomeres were much longer than those (median length of 1,860 bp) found in the genome of *Oropetium thomaeum*[22] (Poaceae), the first published plant genome sequenced by PacBio RS II SMRT.

Fifteen centromere repeats with length >25 kb were also detected on the assembled chromosomes of *N. colorata*, distributed on 12 chromosomes (Chr1, Chr2, Chr3, Ch4, Chr5, Ch6, Ch7, Chr8, Chr11, Chr12, Chr13, and Chr14) and unanchored scaffolds. The length of a single centromere varied from several kb to several hundred kb (Supplementary Table 9). These centromeres generally consisted of repeats with a 115 bp repeat unit (Supplementary Fig. 8), shorter than the average lengths of centromere repeats in most of the other mesangiosperms (150-180 bp)[25].

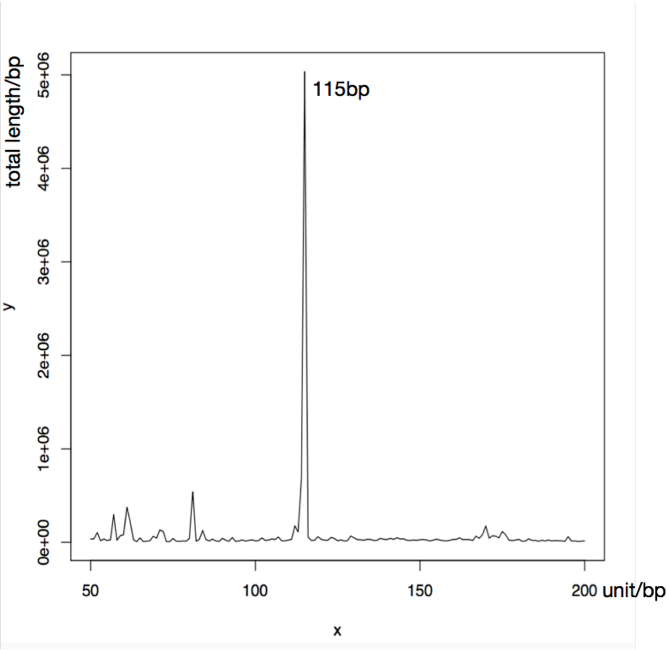

Supplementary Fig. 8 | Distribution of centromere unit length in the genome of *Nymphaea colorata*. The x-axis indicates centromere unit length. The y-axis shows the total length (bp) for each unit. The graph shows that the average length of the centromere unit in *N. colorata* is 115 bp.

3.2 microRNAs

Materials and Methods

A set of experimentally validated miRNA sequences were downloaded from mirBase v21 (www.mirbase.org) and mapped to the *N. colorata* genome using Bowtie2[26] allowing two mismatches. The mapping SAM files were converted into BLAST format using a Perl script available in miRDeep-P v3.1[27]. We filtered sequences with more than 15 mapping hits according to the miRDeep-P manual. Next, miRNA precursor sequences were extracted, with their folding potential predicted using RNAfold v2 (http://hackage.haskell.org/package/RNAFold). Finally, miRDeep-P v3.1 was applied to extract the sequences and structures, calculate the minimum free energy of the potential precursors, and identify mature miRNAs with high confidence.

Results and Discussion

The analysis predicted 125 miRNAs from the *N. colorata* genome (Supplementary Table 10), which were clustered into 77 miRNA families. For comparison, the *Amborella* genome encodes 124 miRNAs representing 90 miRNA families[15].

3.3 Protein-coding genes

Materials and Methods

Genscan[28] (http://genes.mit.edu/GENSCAN.html) and Augustus[29] were used for *de novo* predictions with gene model parameters trained from *Arabidopsis thaliana*. Furthermore, gene models were *de novo* predicted using MAKER[13]. We then evaluated the genes by comparing the MAKER results with the corresponding transcript evidence to select gene models that were the most consistent according to the AED metric[13].

Results and Discussion

We identified 31,580 protein-coding genes in the 409 Mb *N. colorata* genome, which is higher than the 26,846 genes in the much larger *Amborella* genome (706 Mb)[15], suggesting a relatively compact gene space in the *N. colorata* genome. The *N. colorata* had an average gene length of 4,948 bp, which is close to that of *Amborella* (5,665bp) and is considerably longer than the average gene lengths of eudicots (2,196 bp for *Arabidopsis thaliana* and 3,071 bp for *Populus trichocarpa*) and monocots (2,821 bp for *Oryza sativa* and 3,341 bp for *Zostera marina*). The longer gene length is largely due to the longer average total intron length per gene (average number of introns times average intron length per gene) in *N. colorata* (3,797 bp) and *Amborella* (4,720 bp) than in *Arabidopsis thaliana* (907 bp), *P. trichocarpa* (1,894 bp), *O. sativa* (1,861 bp), or *Z. marina* (1,939 bp) (Supplementary Table 11).

3.4 Comparison of gene families from water lily, *Amborella*, eudicots, and monocots

Materials and Methods

The latest version of Pfam-A (version 31) seed alignment sequences were downloaded from the Pfam database (ftp://ftp.ebi.ac.uk/pub/databases/Pfam/releases/). HMMscan from the HMMER software suite[14] was used for gene family identification, with search parameters'--cut_ga –tblout'. Orthogroups were identified using OrthoFinder-0.7.1 with default parameters.

To compare genes from *N. colorata, Amborella*, and mesangiosperms, we sampled the following species: eudicots (*Arabidopsis thaliana*, *Populus trichocarpa*, *Vitis vinifera*, *Solanum lycopersicum*, *Coffea arabica*, and *Nelumbo nucifera*), monocots (*Spirodela polyrhiza*, *Zostera marina*, *Musa acuminata*, *Ananas comosus*, and *Oryza sativa*), gymnosperms (*Gnetum montanum* and *Ginkgo biloba*), and land plants (*Selaginella moellendorffii* and *Physcomitrella patens*). The proteome data for eudicots, monocots, gymnosperms, and land plants were combined and formatted as the BLASTp database. The proteomes of *N. colorata* and *Amborella* were used as queries to search against the database.

To annotate the *N. colorata* genes, we performed gene ontology (GO) analysis, which characterizes gene functions according to biological process, cellular component, and molecular function terms (http://geneontology.org). We first performed BLAST to compare water lily genes with *A. thaliana* genes, and the best BLAST hits were selected to assign function. The *Arabidopsis* IDs of the hits were uploaded to the agriGO[30] online server for GO annotation.

Results and Discussion

The predicted genes in the *N. colorata* genome belong to 4,329 Pfam gene families. To investigate whether water lily had specific gene expansions, we compared the orthogroups from selected gymnosperms (*Ginkgo biloba* and *Gnetum montanum*), ANA-grade angiosperms (*Amborella trichopoda*, *N. colorata*), monocots (*Zostera marina*, *Spirodela polyrhiza*, *Musa acuminata*, *Ananas comosus*, *Oryza sativa*, and *Sorghum bicolor*), and eudicots (*Nelumbo nucifera*, *Vitis vinifera*, *Solanum lycopersicum*, *Beta vulgaris*, *Populus trichocarpa*, and *Arabidopsis thaliana*). A total of 25,120 genes of *N. colorata* had homologues in other plants that we selected and were classified into 9,861 orthogroups (Supplementary Table 12). Mesangiosperms (including six monocots and six eudicots that we sampled) specifically shared 463 orthogroups (Supplementary Fig. 9), but they are not found in *Amborella* and Nymphaeales (genome of *N. colorata* and transcriptomes of 10 representative species). Gene ontology shows high enrichment in oxidation and reduction (redox) reactions and metal-ion binding.

Nymphaeales and mesangiosperms shared 1,331 orthogroups that are absent in *Amborella*. The genes of these 1,331 orthogroups might have been present in the last common ancestor of extant angiosperms and subsequently lost in the lineage leading to *Amborella*, although it is also possible that these genes were present in the last common ancestor of Nymphaeales, eudicots, and monocots after they diverged from the lineage leading to *Amborella*. GO

enrichment analysis of these genes shows a number of genes to be involved in the recognition of pollen and pistil interactions, suggesting that a delicate pollen-pistil interaction mechanism might not be available in *Amborella.*

The 100 largest gene orthogroups are shown in Supplementary Fig. 10. These orthogroups were divided into two clusters, the mesangiosperm-enriched cluster and the water lily-enriched cluster. GO annotation of the mesangiosperm-enriched cluster contained genes for DNA binding, kinase activity, and phosphotransferase activity, which may play overall roles in plant stress-signalling pathways. GO annotation of the water lily-enriched cluster highlighted ADP binding, defence response, terpene synthesis, and hydrolase activity, suggesting that water lily has a unique genetic toolbox.

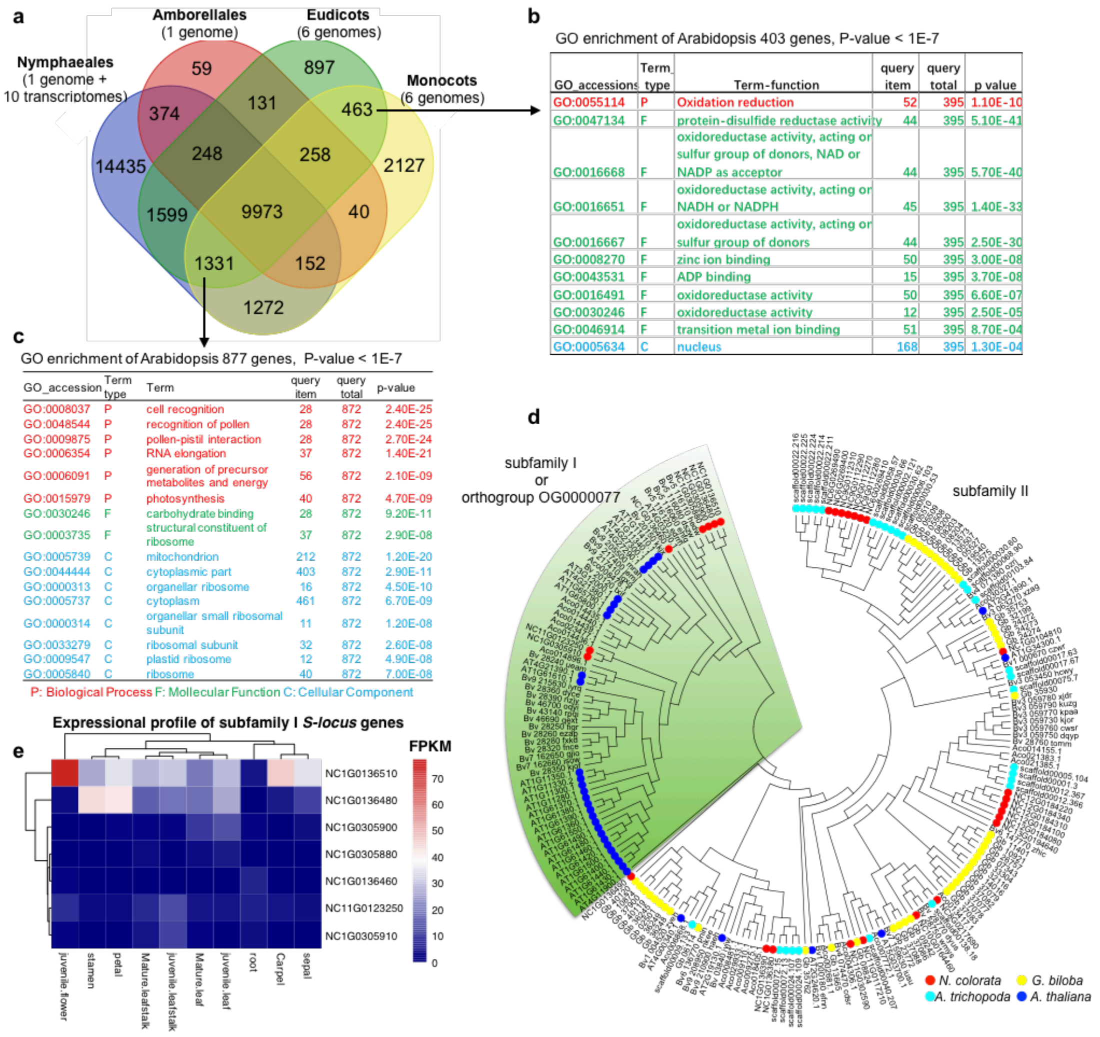

Supplementary Fig. 9 | Analyses of orthogroups shared by Nymphaeales, *Amborella*, eudicots, and monocots. a, Venn diagram showing the genes shared among Nymphaeales, Amborellales, eudicots, and monocots. The ten Nymphaeales species with transcriptome sequences were *Cabomba caroliniana*, *Nuphar advena*, *Nymphaea tetragona*, *Nymphaea caerulea*, *Nymphaea rubra*, *Nymphaea lutea*, *Nymphaea mexicana*, *Victoria cruziana*, *Brasenia schreberi*, and *Euryale ferox*. The six eudicots are *Arabidopsis thaliana*, *Populus trichocarpa*, *Beta vulgaris*, *Solanum lycopersicum*, *Vitis vinifera*, and *Nelumbo nucifera*. The six monocot species are *Oryza sativa*, *Sorghum bicolor*, *Ananas comosus*,

Musa acuminata, *Spirodela polyrhiza*, and *Zostera marina*. **b,** GO enrichment of 463 genes shared by the eudicots and monocots shows they are involved in oxidation and reduction reactions. **c,** GO enrichment shows that compared with *Amborella*, Nymphaeales shared more genes with monocots and eudicots, with 1331 genes (corresponding to 877 *Arabidopsis* genes) mainly involved in pollen-pistil interaction and energy metabolism, **d**, Phylogenetic tree of the S-locus gene family from representative seed plant species. This tree could be divided into two subfamilies I and II. **e**, Expression profile of the 7 subfamily I *S*-locus genes in different organs of *N. colorata*. GO annotation used *t*-test and two-sided test. Multiple test adjustment was performed.

The orthogroup OG000077 for self-incompatibility (*S*)-locus genes is present in water lily. Together with the expression in specific floral organs of the two S-locus genes (NC1G0136510 and NC1G0136480), this suggests that *S*-RNase mediated pollen recognition was probably already present in the common ancestor of water lily and core angiosperms, but not in *Amborella* or in gymnosperms (Supplementary Fig. 9). Also, the orthogroup OG000042 is found in *N. colorata* and in members of the mesangiosperms, but not found in *Amborella*, and encodes *h*AT-like transposase (Supplementary Fig. 11), which is essential for *Arabidopsis* development[31]. The orthogroup OG000234 contains auxin response genes, some of which have been shown to regulate the flower opening and closure[32]. We identified 7 genes in *N. colorata* from orthogroup OG000234, but none was found in *Amborella*. The presence of these genes in Nymphaeales, eudicots, and monocots suggests that these are derived features shared by Nymphaeales and mesangiosperms.

We then used BLASTp to compare *N. colorata* and *Amborella* proteins to those in the representative seed plants. When *N. colorata* proteins were used as the query, *Amborella* had the most similar orthologues, followed by lotus, grape, poplar, pineapple, ginkgo, and banana (Supplementary Fig. 12). These results revealed a greater degree of similarity between water lily and eudicots. When *Amborella* proteins were used as the query, lotus had the most similar orthologues, followed by grape, water lily, poplar, ginkgo, banana, and pineapple. These results showed that lotus and grape shared more conserved features with *N. colorata* and *Amborella*. In particular, the analysis revealed that ginkgo had more similarities with *Amborella* than with *N. colorata*, suggesting that *Amborella* proteins aremore conserved and had a slower rate of evolution.

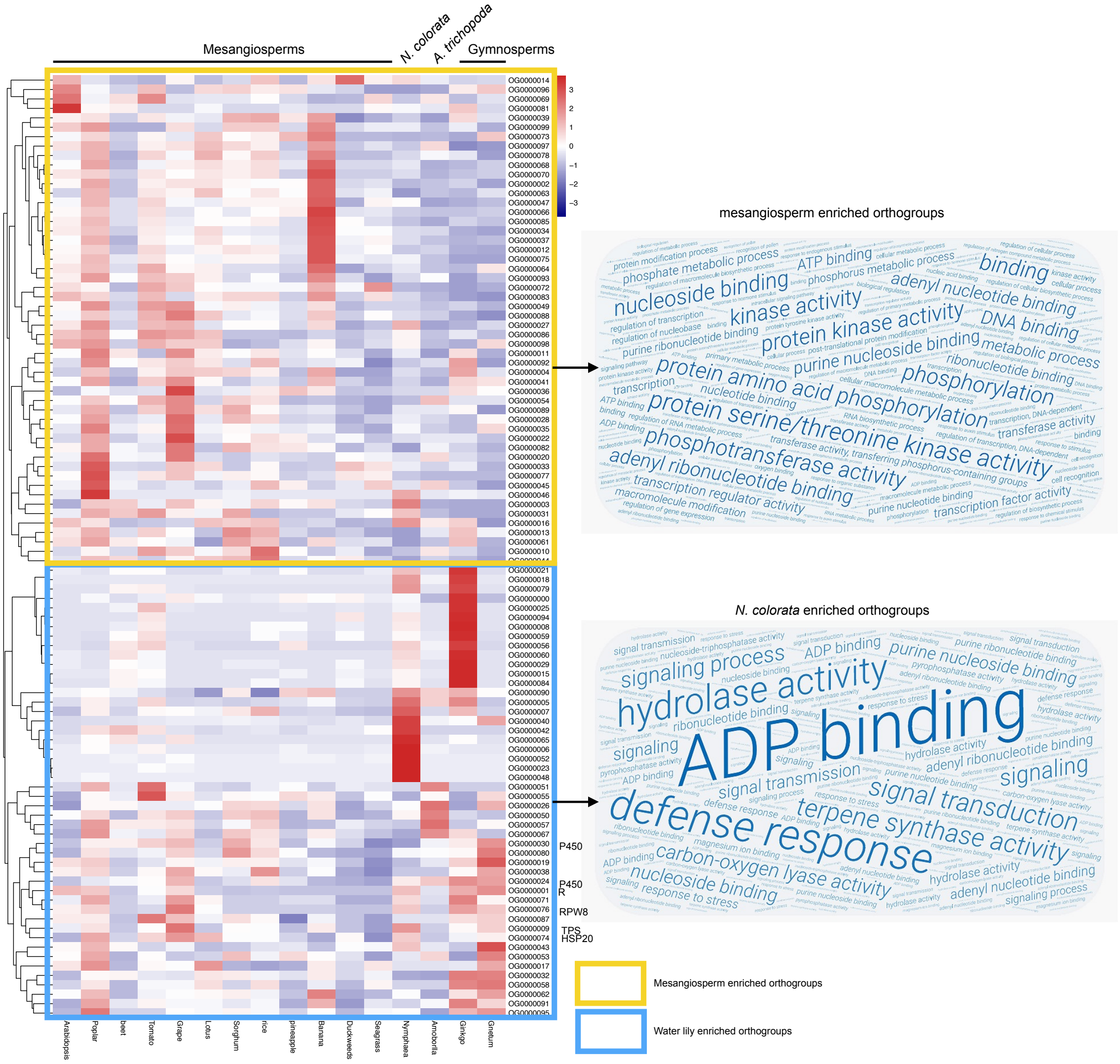

Supplementary Fig. 10 | Orthogroup-based comparison shows that water lily has intermediate angiosperm features. Comparison of the number of orthologous genes in each orthogroup (as shown in the heatmap) in representative clades of seed plants reveals a subset of genes specific to eudicots and monocots (yellow box) and another subset of genes enriched in water lily but not found in *Amborella* (blue box). The word cloud diagrams on the right show the enriched terms associated with the orthogroups in each subset.

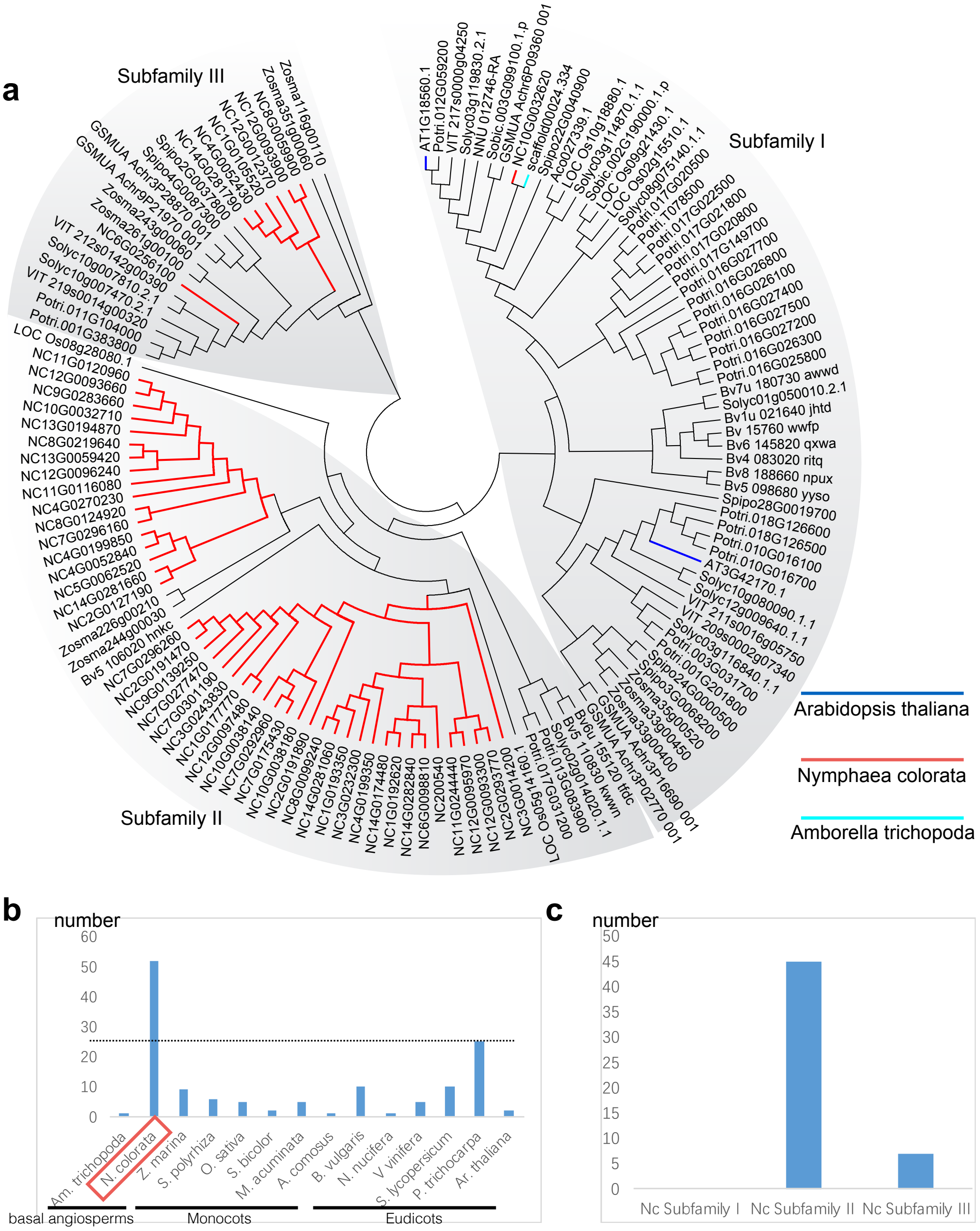

Supplementary Fig. 11 | The expansion of *hAT* genes in the *Nymphaea colorata* genome. a, The phylogenetic tree divided the *hAT* gene family into three subfamilies in angiosperms. **b**, Comparison of *hAT* gene number in *N. colorata*, monocots, and eudicots showed that *N. colorata* had the most *hAT* genes. **c**, The number of *N. colorata hAT* genes in the three subfamilies shows that most of the expansion was due to the expansion of subfamily II.

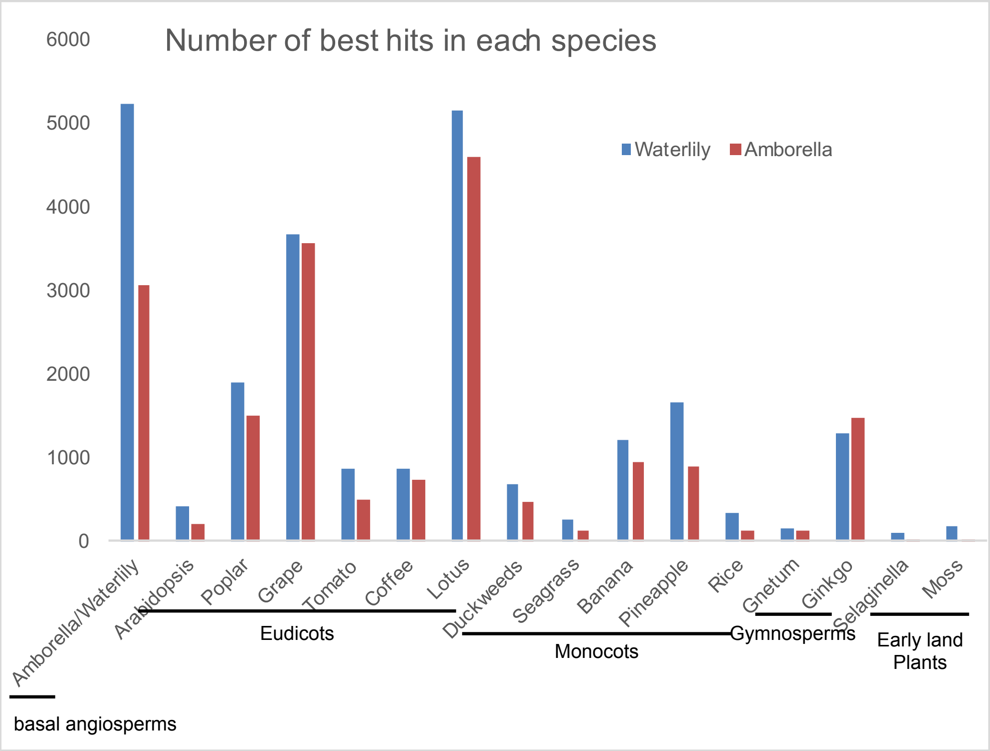

Supplementary Fig. 12 | The number of first BLASTp hits in representative plant taxa using *Nymphaea colorata* (blue) or *Amborella trichopoda* (red) sequences as the query.

3.5 Organellar genomes

Materials and Methods

The organellar sequences were identified using BLASTn against the *N. colorata* genome contigs with plant organellar sequences as references

(https://www.ncbi.nlm.nih.gov/genome/browse/?report=5#!/overview/). Two mitochondrial contigs, which were 527,532 bp (tig00000378) and 136,812 bp (tig00000456) in length, overlapped with each other at both ends by 34,745 bp and 16,132 bp. The analysis indicated a circular 617,195 bp sequence (ChrM), with an average read depth of 601×. One chloroplast contig 178,451 bp in length (tig00000521/ChrC) overlapped at its two ends by 18,738 bp, indicating a circular sequence with a length of 159,713 bp. The organellar genome sequences were annotated as previously described[33]. Protein-coding genes and rRNA genes were annotated by BLASTn searches against the NCBI non-redundant database. The exact gene and exon/intron boundaries were further confirmed using Geneious software (v.10.0.2, Biomatters, www.geneious.com) by aligning each gene to orthologues from the available annotated plant organellar genomes.

Results and Discussion

We reconstructed a complete circular mitochondrial genome of 617,195 bp and a circular chloroplast genome of 159,824 bp. The *N. colorata* mitochondrial genome contains 41

protein-coding genes (**Supplementary Fig. 13**) along with three rRNA and 20 tRNA genes. Since repeat sequences occupy 48.89% of the mitochondrial genome, *N. colorata* has one of the most repeat-rich mitogenomes among angiosperms. Although many foreign insertions, including the entire moss mitogenome, were identified in the *Amborella* mitogenome[34], none was observed in the *N. colorata* mitogenome.

The complete *N. colorata* chloroplast genome comprised four parts: long single copy section (LSC), inverted repeats (IRB), small single copy section (SSC), and inverted repeat A (IRA). The *N. colorata* chloroplast genome encodes 136 genes (**Supplementary Fig. 14**), which is slightly more than the 132 genes in the *Amborella* chloroplast genome[35]. The complete *N. colorata* chloroplast genome will be a valuable resource for studying the evolution of angiosperms. Taken together, the water lily genome from all three cellular compartments (chloroplast, mitochondria, and nucleus) is of high contiguity and low error rate, making it an excellent reference for comparative and evolutionary studies.

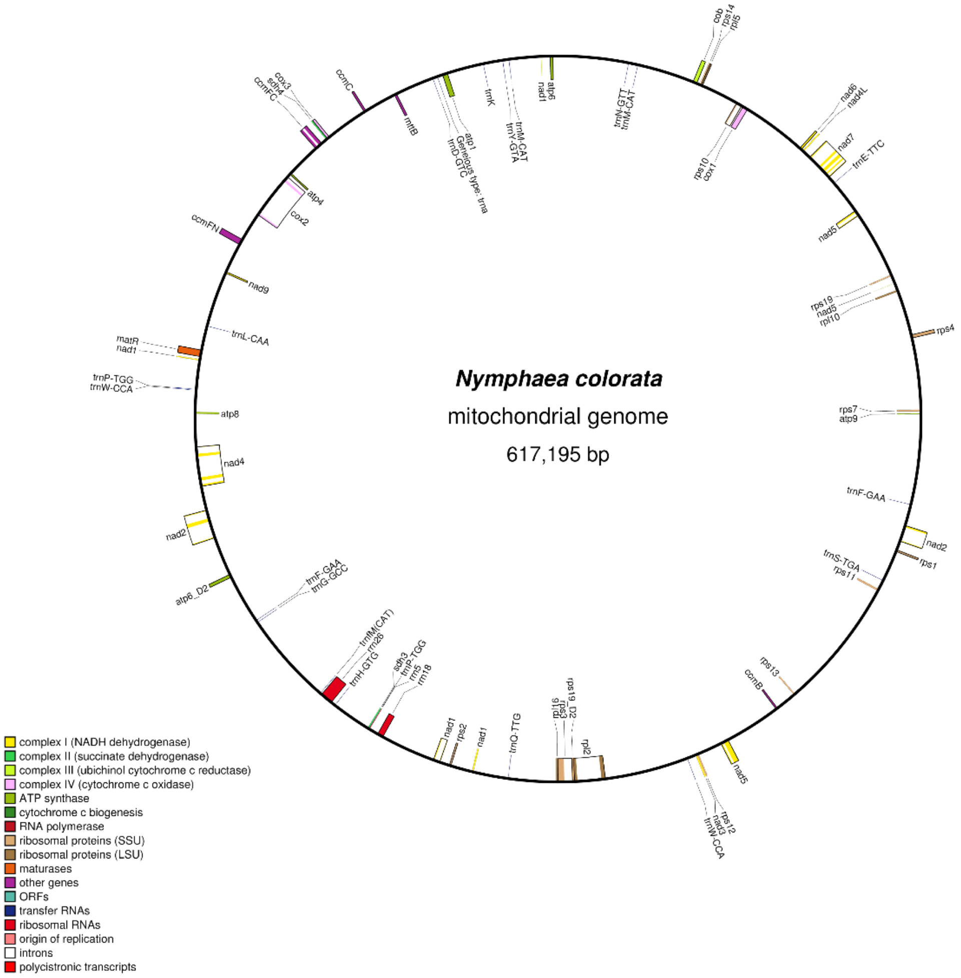

Supplementary Fig. 13 | The circular mitochondrial genome of *Nymphaea colorata*. The figure was generated using Organellar Genome DRAW (http://chlorobox.mpimp-golm.mpg.de/OGDraw.html). Colour-coded boxes indicate the genes in the genome.

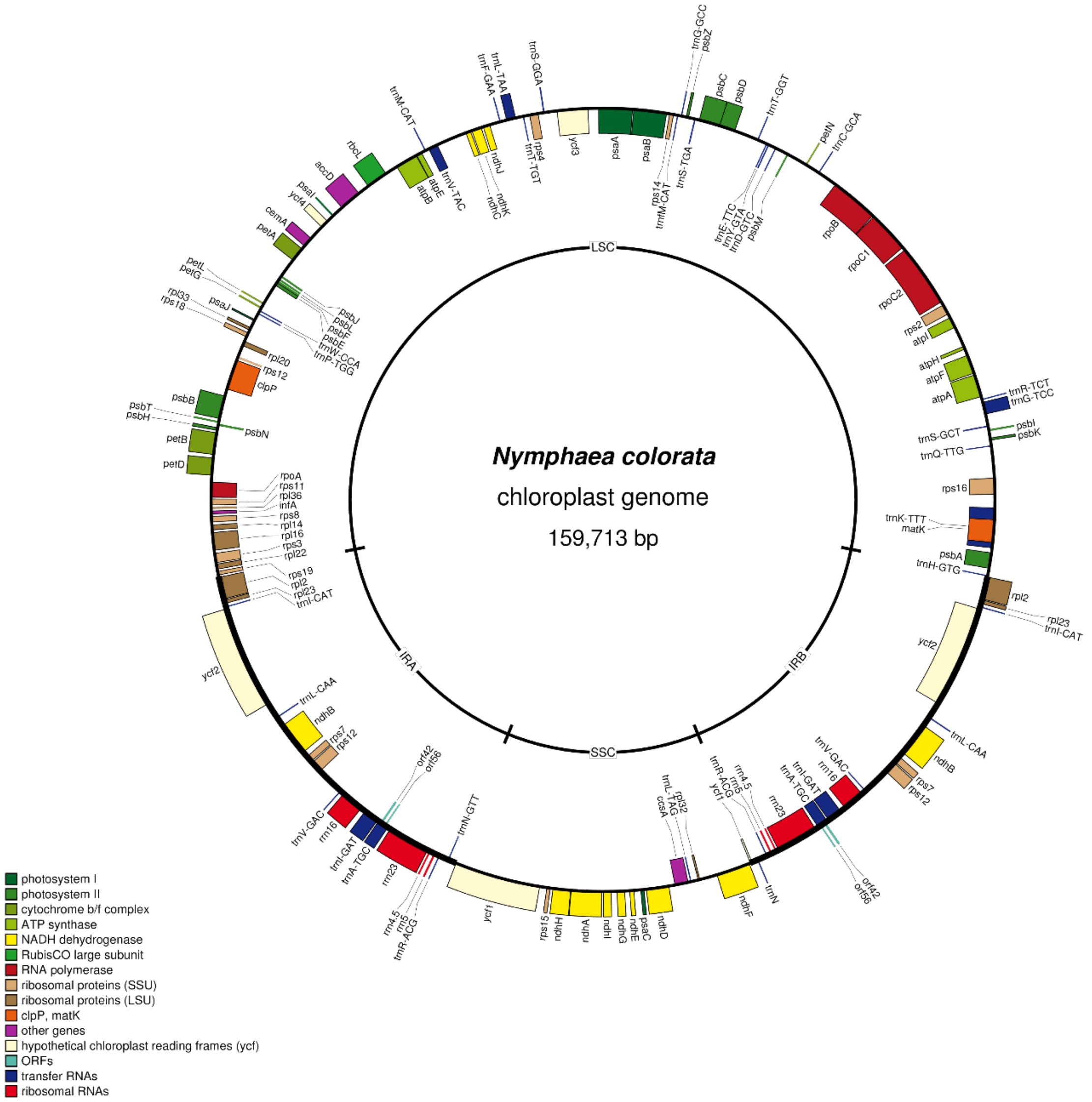

Supplementary Fig. 14 | The circular chloroplast genome of *Nymphaea colorata*. The figure was generated using Organellar Genome DRAW (chlorobox.mpimp-golm.mpg.de/OGDraw.html). Colour-coded boxes indicate the genes in the genome.

3.6 Transcriptome assembly and expression quantification

Materials and Methods

Transcriptomes from various water lilies were sequenced using the illumina platform; 18 Nymphaeales species (*N. colorata*, *N. mexicana*, *N. prolifera*, *N. tetragona*, *N. potamophila*, *N. rubra*, *N. caerulea*, *N.* 'midnight', *N.* 'Choolarp', *N.* 'Paramee', *N.* 'Woods blue goddess', *N. gigantea* 'Albert de Lestang', *N. gigantea* 'Hybrid I', *N.* 'Thong Garnjana', *Victoria cruziana*, *Euryale ferox*, *Nuphar lutea*, and *Brasenia schreberi*) and various organs and tissues from *N. colorata* (mature leaf, mature stem, juvenile flower, juvenile leaf, juvenile stem, carpel, stamen, sepal, and petal) were sampled (Supplementary Table 1). High-quality reads were obtained by removing adaptor sequences and filtering low-quality reads using TRIMMOMATIC[36] with default parameters. The resulting high-quality reads were *de novo* assembled using Trinity[37]. Protein sequences and coding sequences of transcripts were predicted using TransDecoder

(http://transdecoder.github.io). Redundant transcripts were removed by CD-HIT (http://weizhong-lab.ucsd.edu/cd-hit/) with a 98% identity cutoff for protein-coding transcripts. Within the Nymphaeales order, only two transcriptomes, *Nuphar advena* and *Cabomba caroliniana*, were downloaded from NCBI-SRA (SRX018920 and SRX3469536, respectively). The data were then assembled and annotated in this study. Other transcriptome datasets used in the tree of 115 plant species were previously published data[38] provided by Dr. Hong Ma from Fudan University (presently at Penn State University). Transcript abundance levels were normalized using the fragments per kilobase per million mapped reads (FPKM) method by Tophat[39] and Cufflink[40].

Results and Discussion

The details of the transcriptome assembly and annotation are listed in Supplementary Table **1**. Transcriptome sequences and annotations are available on our online water lily genome database (http://waterlily.eplant.org). Overall, these transcriptomes are well assembled and annotated, with contig N50 ranging from 587 bp to 1,870 bp. Unlike the sequenced genome of *N. colorata* with a GC content of 0.386, all of the transcriptomes have higher GC contents ranging from 0.415 to 0.432. This difference is consistent with the fact that transcriptomes mainly correspond to gene transcripts that are typically GC-rich rather than repetitive sequences.

4. Resolving deep phylogenetic relationships among Amborellales, Nymphaeales, and core angiosperms

4.1 Phylogenomic discordance among low-copy nuclear gene trees

Whether the order Nymphaeales or the order Amborellales, or a clade containing both Nymphaeales-Amborellales, is sister to all other angiosperms (Fig. a, illustrated as Type I, II, III trees, respectively). The *N. colorata* genome provides a new opportunity to use nuclear genes to address this important question. We performed a series of phylogenomic analyses with the newly annotated *N. colorata* genes and sequences from other available seed plant genomes and transcriptomes (see Methods). Using six representative eudicots, six representative monocots, and three different gymnosperm species (*G. biloba*, *P. abies* and *P. taeda*, Supplementary Table 6) as a different outgroup species, 2,169, 1,535, and 1,515 orthologous low-copy nuclear (LCN) genes were identified, respectively (see Methods) (Fig. 1b). Gene trees were generated using nucleotide sequences of these LCN genes with each one of the three gymnosperms as an outgroup. Among the LCN gene trees using *G. biloba* as an outgroup and with > 80% BS values, 62% (294 out of 475 trees) support *Amborella* as the earliest diverging lineage among extant angiosperms (Type II, Fig. 1c). Similarly, using *P. abies* or *P. taeda* as the outgroup species, 57% and 54% of the LCN gene trees, respectively, support *Amborella* as the earliest diverging angiosperm. In contrast, only 10%, 14%, 11% of the LCN gene trees that support Nymphaeales as the earliest diverging angiosperm (Type I, Fig. 1c) using the three above-mentioned outgroups, respectively; 28%, 29% and 35% of the LCN gene trees, respectively, support a clade of both Nymphaeales and *Amborella* as sister to all other angiosperms (Type III, Fig. 1c). LCN gene trees using amino acid sequences also show similar patterns that support *Amborella* as the sister group to all other extant angiosperms (see Methods and Supplementary Fig. 15).

The observation of the three different topology of gene trees can potentially be explained by incomplete lineage sorting (Supplementary Table 13), which could appear more frequently when evolutionary divergences have occurred in rapid succession. However, it is not clear whether the divergence events in the ANA-grade of angiosperms were particularly rapid, given the lack of fossils before the Early Cretaceous[41]. The phylogenetic discordance could also be due to an erosion of the historical signal, in view of the deep phylogenetic scale of the ANA-grade. For example, we observed that in *N. colorata*, genes supporting Type I trees tend to have higher substitution rates than those supporting Type II and Type III trees (Supplementary Fig. 16). This suggests that differences in substitution rates might contribute to the uncertainty in the placement of Nymphaeales. To account for incomplete lineage sorting and uneven substitution rates, we applied the multispecies coalescent model and a supermatrix method, respectively, to the LCN genes and found further support for the sister relationship between *Amborella* and all other extant flowering plants (Supplementary Fig. 17).

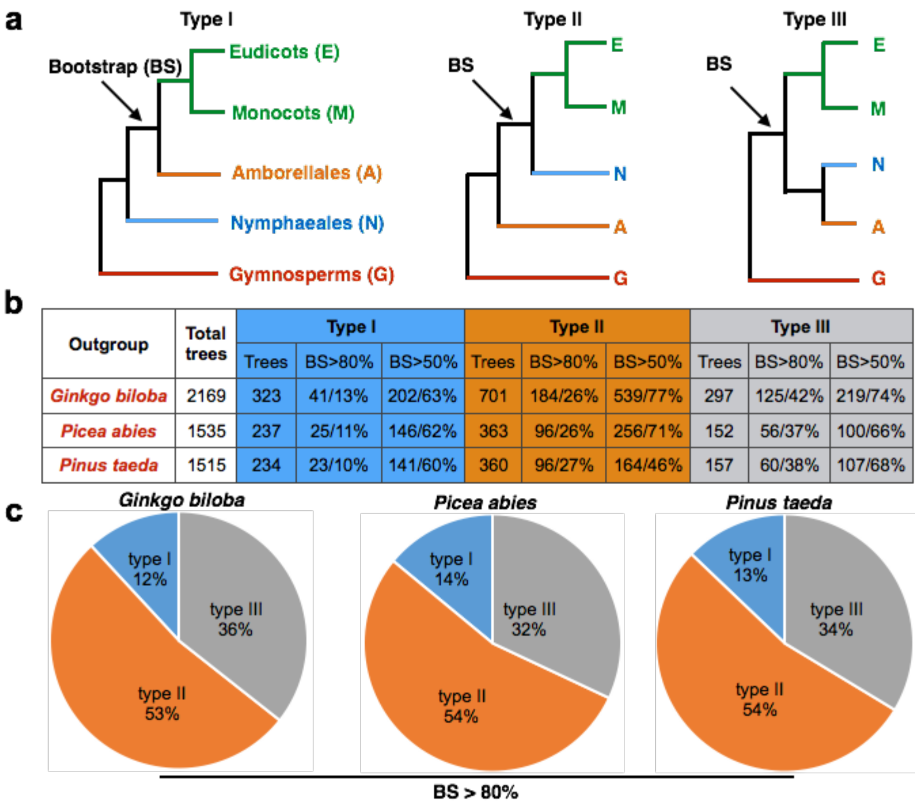

Supplementary Fig. 15 | Inference of the species tree for *Amborella* and water lilies using protein data. a, Three extant evolutionary scenarios for the major angiosperm clades. **b**, Statistics of single-copy nuclear genes for the phylogenetic tree based on proteins using one of three different gymnosperms as the outgroup. **c**, Tree numbers supporting the different species trees using one of three gymnosperm species. These results based on protein data support *Amborella* as the sister lineage to all other angiosperms and are consistent with the coding sequence data shown in **Fig. 1**.

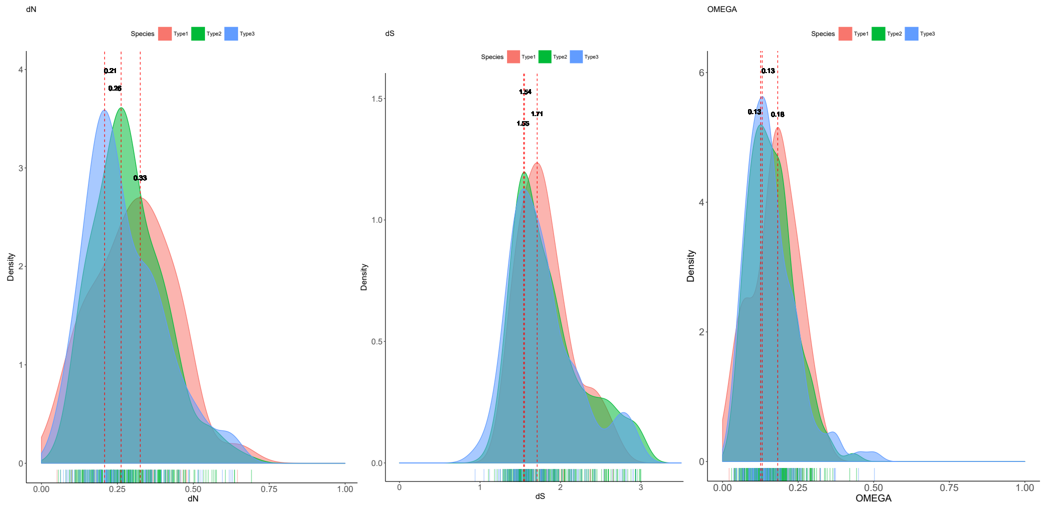

Supplementary Fig. 16 | Comparison of substitution rates among three types of low-copy nuclear genes used in Fig. 1. a, Distribution of the nonsynonymous substitution rate (*dN*) among the type I, type II, and type III low-copy nuclear (LCN) genes. **b**, Distribution of the synonymous substitution rate

(*dS*) among the three types of LCN genes. **c**, Distribution of omega values (*dN*/*dS*) among the three types of LCN genes. Red, Type I; green, Type II; blue, Type III.

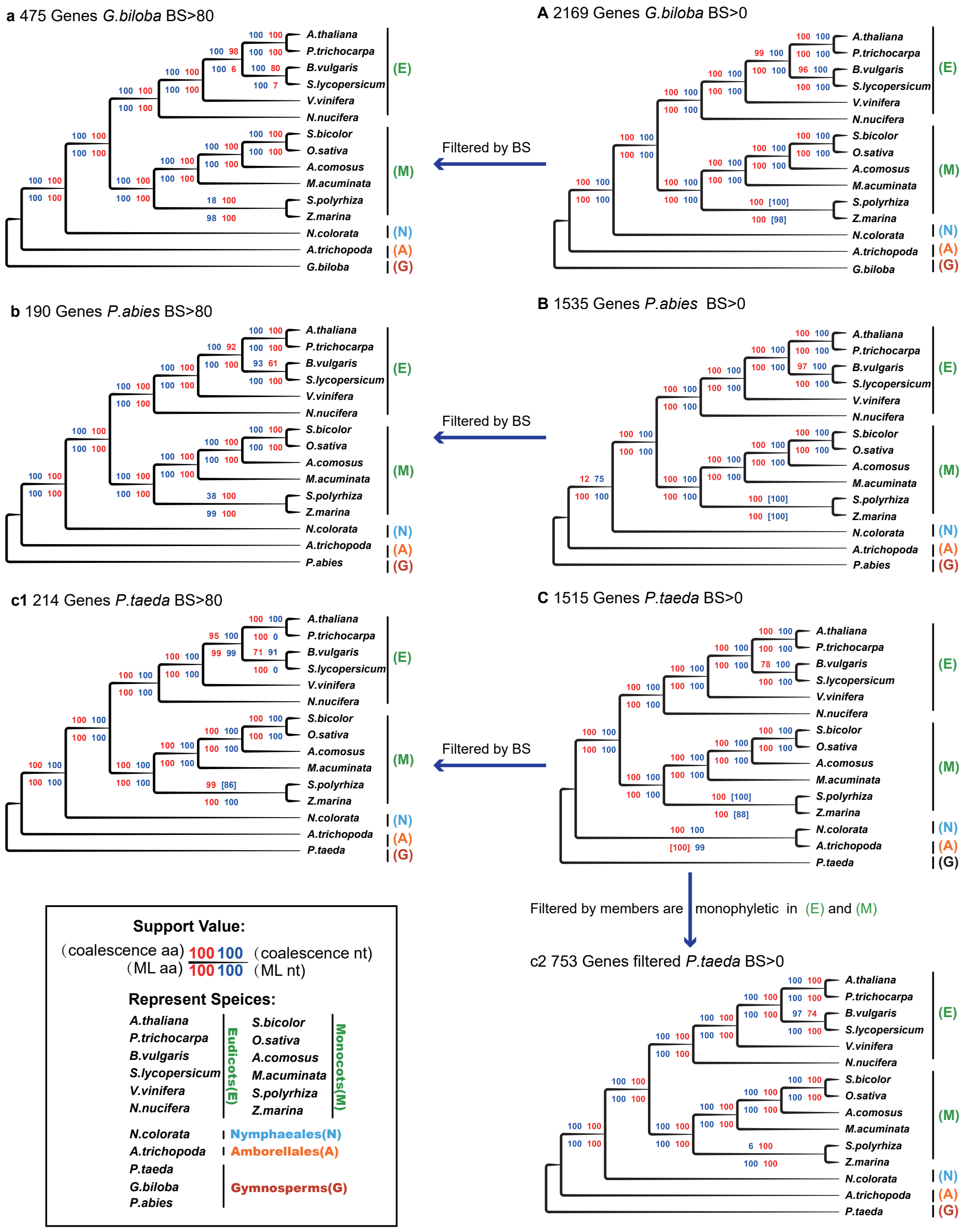

Supplementary Fig. 17 | Summary of phylogenetic trees inferred from different outgroups, methods, and types of gene markers. The statistics for all the orthologous groups (OGs) and species used here are shown in **Fig. 1b-1c** and **Supplementary Figure. 15**. Blue and red values indicate support values inferred from nucleotide and amino acid sequences, respectively. Support values above and below the branches were inferred using the multispecies coalescent and supermatrix methods, respectively. **A**, Summary of trees inferred from 2,169 OGs with single gene tree BS >0 and *Ginkgo biloba* as outgroup. **B**, Summary of trees inferred from 1,535 OGs with single gene tree BS >0 and

Picea abies as outgroup. **C**, Summary of trees inferred from 1,515 OGs with single gene tree BS >0 and *Pinus taeda* as outgroup. Summarized trees of a, b, and c1 were inferred from filtered OGs by main nodes BS >80. The 753 OGs used in c2 were filtered according to monophyly; if members of a given OG were not grouped together in eudicots and monocots, they were considered potentially paralogous and discarded.

4.2 Using low-copy nuclear genes to infer the tree and molecular dating of angiosperms

Materials and Methods

All of the angiosperm genomes used in this study were downloaded from the angiosperm genome database (http://www.angiosperms.org)[42] and the eplant database (www.eplant.org). To infer the angiosperm phylogeny we selected the following plant genomes as representatives for each clade: gymnosperms (*Picea abies*, *Ginkgo biloba*, and *Pinus taeda*; see assembly and annotation details in Supplementary Table 6), ANA-grade angiosperms (*Amborella*), eudicots (*Arabidopsis thaliana*, *Beta vulgaris*, *Populus trichocarpa*, *Solanum lycopersicum*, *Vitis vinifera*, and *Nelumbo nucifera*), and monocots (*Oryza sativa*, *Sorghum bicolor*, *Ananas comosus*, *Musa. acuminata*, *Spirodela polyrhiza*, and *Zostera marina*).

Because angiosperms have undergone several rounds of whole-genome duplication (WGD) events and subsequent gene losses, LCN orthologous groups (OGs) were selected as phylogenetic markers. To avoid possible biases of specific gene sets and the loss of potentially informative nuclear gene markers, candidate marker genes were retrieved from three groups (see pipeline in Supplementary Fig. 18).

The first group with an intersecting gene set of 931 OGs from two orthologous gene datasets was previously identified for an analysis of deep angiosperm phylogeny[38]. One dataset contained 4,180 OGs shared by nine angiosperm species with sequenced genomes (*Arabidopsis thaliana*, *Populus trichocarpa*, *Glycine max*, *Medicago truncatula*, *Vitis vinifera*, *Solanum lycopersicum*, *Oryza sativa*, *Sorghum bicolor*, and *Zea mays*) identified by HaMStR[36] (Deep Metazoan Phylogeny, http://www.deep-phylogeny.org/hamstr/). The other dataset contained 1,989 low-copy OGs identified using seven angiosperm species with sequenced genomes (*A. thaliana*, *P. trichocarpa*, *Prunus persica*, *V. vinifera*, *Mimulus guttatus*, *O. sativa*, and *S. bicolor*) identified using OrthoMCL v1.4[43] with default parameters. We selected the 931 OGs shared by the two datasets as phylogenetic markers representing conserved low-copy OGs across angiosperms.

To minimize the effect of hidden paralogues and identify the most probable orthologues, we carefully retrieved OGs from low taxon levels. The second group comprised 407 OGs carefully selected by gene length and species coverage ratio among 125 Rosaceae species; these were previously identified for a phylogenetic study of Rosaceae[44] and provide greater phylogenetic signal. The third group comprised 852 OGs for a phylogenetic study of Brassicaceae[45], and they were similarly selected by species coverage ratio and gene length.

Redundant sequences from the same genes were removed, resulting in 1,167 high-quality putative orthologous genes that were used to search for homologues in the other 115 flowering plant genomes and transcriptomes using HaMStR[36].

Sequences for each orthologous group were aligned using MAFFT v7.221[46] with the option "- auto", followed by manual adjustment to remove gaps using MEGA6[47]. Next, trimAL 1.4[48] was used to trim low-quality aligned regions with the option "-automated1". To reconstruct the deep phylogenetic relationships among angiosperms, we used the alignment of coding sequences (nucleotides) to generate a maximum likelihood (ML) tree. The coding sequences were converted from the protein alignment matrix and aligned by PAL2NAL[49]. ModelFinder[44] was used to select the best-fit model under the Bayesian information criterion (BIC). Phylogenetic reconstruction was performed stepwise with several carefully selected gene sets (1167, 834, 683, 602, and 445; see explanations in the next sentences) using the coalescence method implemented in ASTRAL v5.5.12[50]. From the 1,167 OGs, 834 OGs had length of at least 840 bp and a species coverage of 80% or more; 683 OGs were at least 1000 bp in length and had a species coverage 90% or more. To resolve the angiosperm deep phylogeny, it was necessary to exclude possible noise from paralogous genes and avoid systematic error arising from the large supermatrix. Therefore, 602 OGs were selected from the set of 834 OGs based on the topology of the low-copy gene trees exhibiting monophyly of each of Angiosperms, Eudicots, Monocots, and Gymnosperms. From the 602 OGs, 445 were selected that showed monophyly of each of Nymphaeales, Asterids, and Rosids. Finally, we selected 101 genes for further ML analysis based on the topology of the low-copy gene tree with each order among our taxon sampling as a monophyletic group. A maximum likelihood (ML) analysis was also performed with the 101 sequence supermatrix using RAxML v7.0.4[51] under the GTR+GAMMA+I model defined as the best-fit evolutionary model. Low-copy gene trees were reconstructed using RAxML v7.0.4 under the GTR+CAT model instead of the GTR+GAMMA model for computational efficiency. For each low-copy gene tree, 100 bootstrap replicates were generated for the coalescent analysis.

The workflow for the orthogroup identification used for species tree reconstruction.

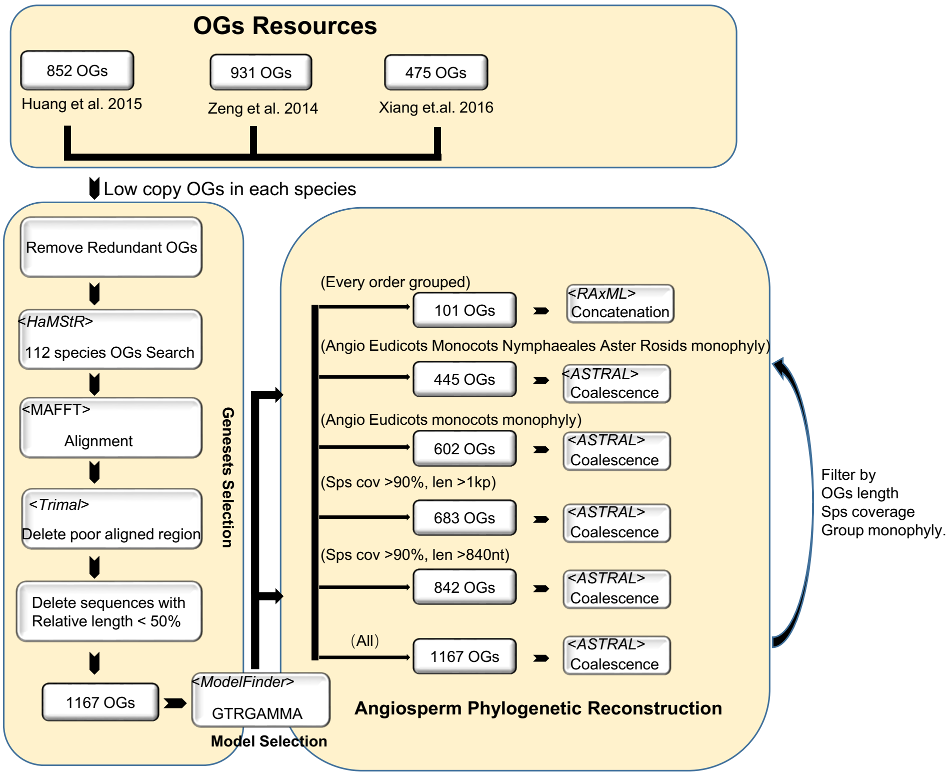

Supplementary Fig. 18 | Workflow for identifying the orthogroups used for species tree reconstruction in Fig. 1d. The complete pipeline was described in the Methods section of this supplementary file.

Molecular dating was carried out using a stringent set of 101 LCN genes (205,185 sites), together with 21 fossil-based age constraints on internal nodes of the tree (Supplementary Table 14). The tree topology was fixed to that inferred in our coalescent-based analysis of 1167 genes from 115 taxa. We performed a Bayesian phylogenomic dating analysis of the 101 selected genes in MCMCtree, part of the PAML package[52], and used approximate likelihood calculation for the branch lengths to improve computational tractability[53]. Molecular dating was performed using an auto-correlated model of among-lineage rate variation, the GTR substitution model, and a uniform prior on the relative node times. Posterior distributions of node ages were estimated using Markov chain Monte Carlo sampling, with samples drawn every 250 steps over 10 million steps following a burn-in of 500,000 steps. We checked for convergence by running the analysis in duplicate and checked for sufficient sampling using Tracer[53,54]. The results of this dating analysis are shown in Fig. 1d.

For comparison, we also inferred the divergence times of angiosperms using penalized likelihood in TreePL[55] and in r8s[56]. This approach was used because we rejected the hypothesis of a strict molecular clock (p-value < 0.01) using a likelihood-ratio test in PAUP 4.0 beta10[57]. To optimize the smoothing parameter for the data, we performed cross-validation and tested a range of smoothing parameters from 0.01 to 100,000 (algorithm=TN; crossv=yes; cvstart=-2; cvinc=0.5; cvnum=15). We identified an optimal value of 0.32 for the smoothing parameter, indicating the presence of substantial rate heterogeneity among branches. We used 100 bootstrap replicates in RAxML[51] to produce a set of input trees for calculating the 95% confidence intervals on our date estimates.

In treePL, the “prime” option was applied to optimize the parameters, and a “thorough” analysis was then carried out with the optimal parameters determined above (opt = 2, optad = 2 and optcvad = 2). To identify the best smoothing parameter, a ‘random subsample and replicate cross-validation was conducted with treePL. The best smoothing value was found to be 0.1 under the lowest chisq value. 95% confidence intervals for the node-age estimates were calculated following previously published methods. To allow for variation in branch-length estimates, we calculated 100 bootstrap replicates with the tree topology fixed to that of the above maximum-likelihood phylogram but with varying branch lengths. We then conducted treePL on these 100 replicates. Age statistics for all nodes were summarized with TreeAnnotator v.1.7[58].

Results and Discussion

Using four gymnosperms, *Ginkgo biloba*, *Picea abies*, *Pinus taeda*, and *Gnetum montanum*, together as the outgroup, the flowering plants could be divided into six lineages: *Amborella*, Nymphaeales, *Illicium*, monocots, magnoliids, and eudicots. Magnoliids clustered with eudicots, supporting the mesodicots superclade[30]. In the species tree in Supplementary Fig. 19, each node shows the supporting values from five methods, with the outgroup consisting of four gymnosperm species. Supplementary Fig. 20 shows a tree inferred using the concatenation method from the supermatrix with 101 low-copy nuclear genes. Taken together, the phylogenetic trees inferred from different outgroups, methods, and types of gene markers all supported that *Amborella* represents the sister lineage to all other extant angiosperms, with 100 high support values using three methods.

Our penalized-likelihood analyses placed the crown age of angiosperms placed at 231.45-251.58 and 248.83-250.45 million years ago (Ma) for TreePL and r8s, respectively (Supplementary Table 15). We dated the crown group of Nymphaeales at 133.00-175.42 and 146.88-148.64 Ma using the two methods, respectively. Both TreePL (Supplementary Fig. 21) and r8s (Supplementary Fig. 22) produced date estimates that were similar to those obtained using Bayesian relaxed-clock analysis, although the r8s method yielded much narrower 95% confidence intervals for each node.

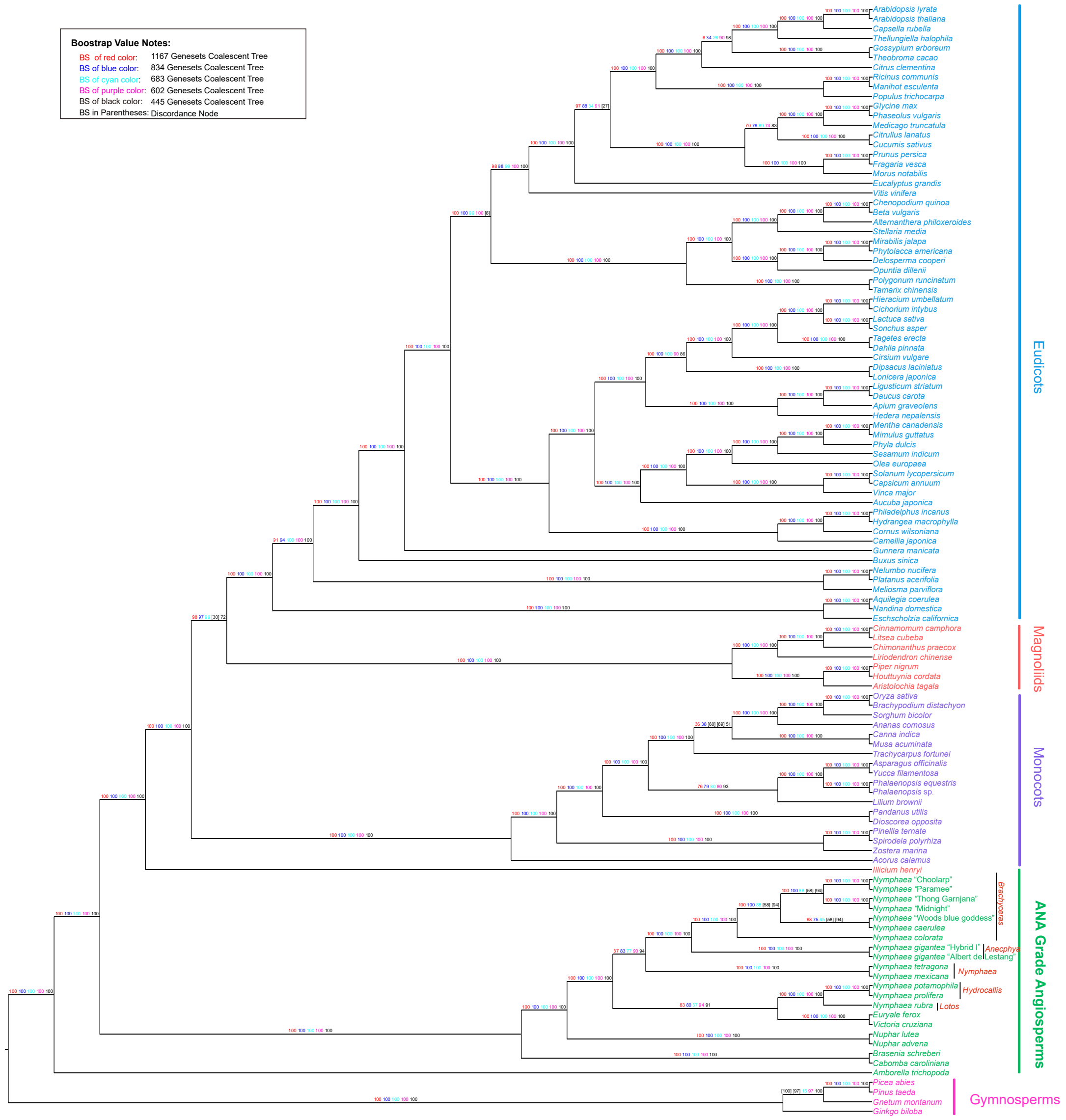

Supplementary Fig. 19 | The species tree of 115 seed plants inferred from five gene sets, with bootstrap values corresponding to Fig. 1d. Support values corresponding to 1167, 834, 683, 602, and 445 low-copy nuclear genes under the multispecies coalescent model implemented in ASTRAL (version 5.5.12).

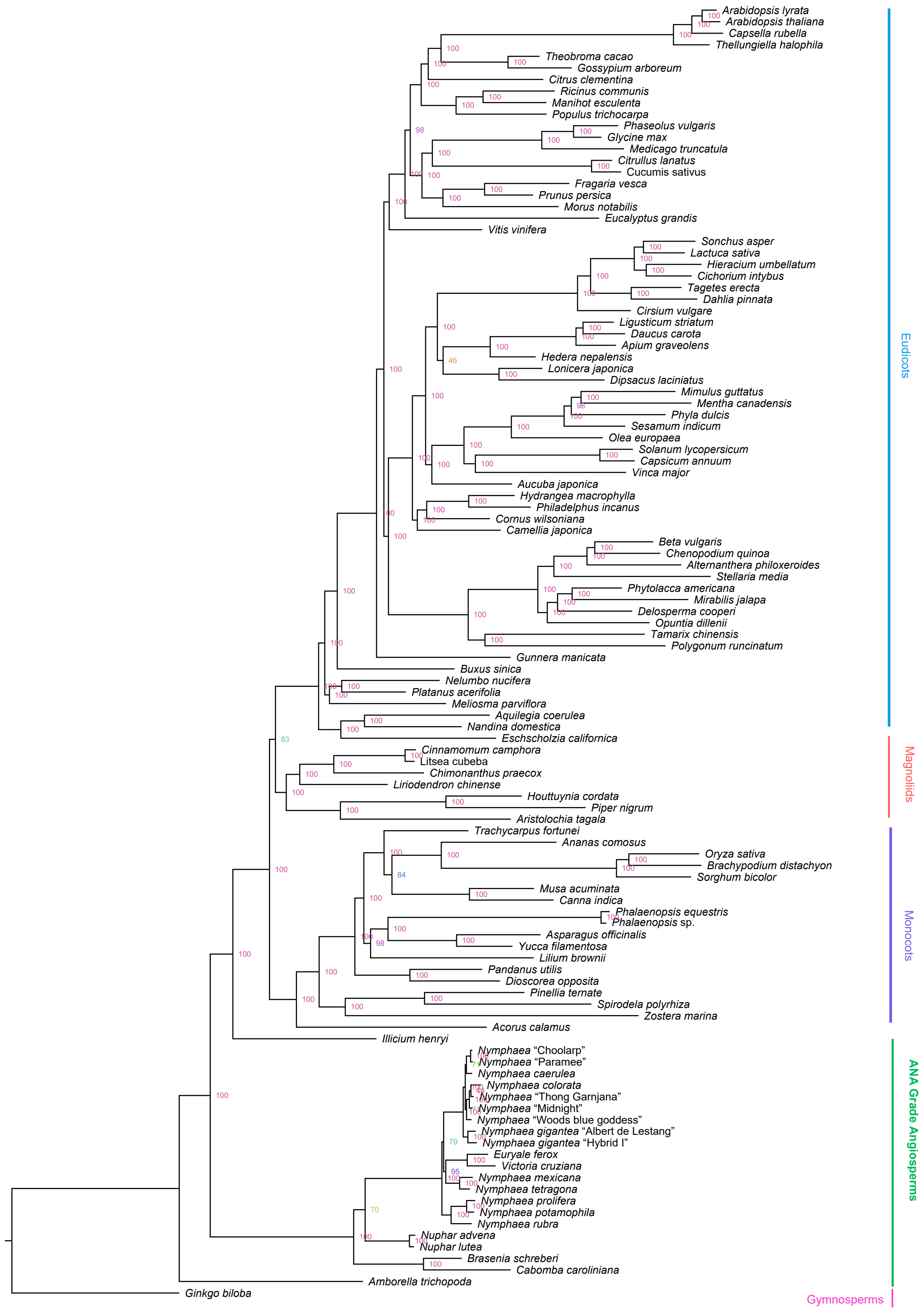

Supplementary Fig. 20 | The 101 low-copy nuclear gene supermatrix-based species tree showing that *Amborella*, and not Nymphaeales, is the sister group to all other angiosperms. Support values are shown on each node, with different colours indicating different levels of bootstrap support.

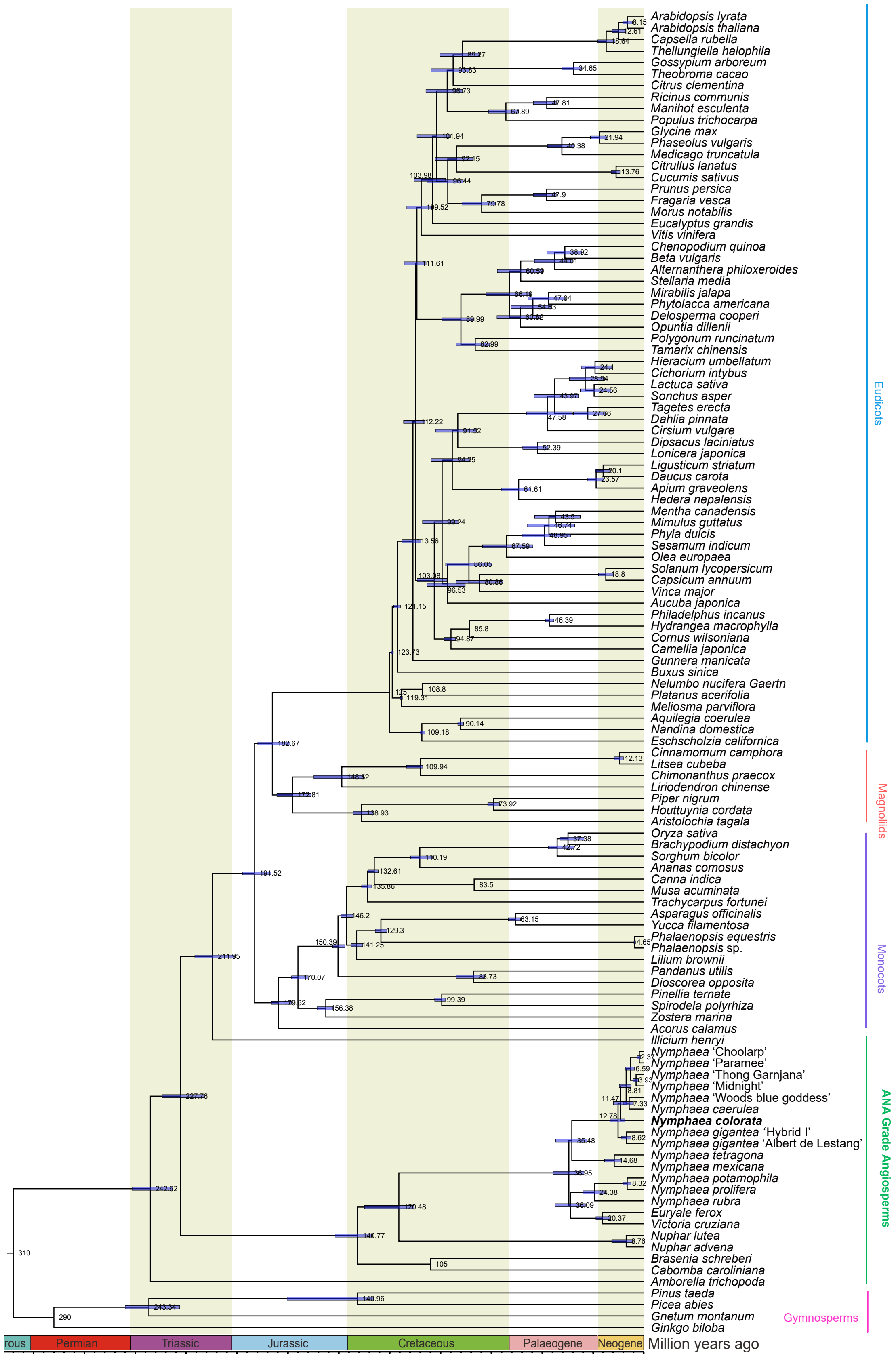

Supplementary Fig. 21 | Estimation of divergence times of 115 seed plants based on penalized likelihood in TreePL. The 95% confidence intervals are shown on each node. The tree topology corresponds to that of **Fig. 1d**.

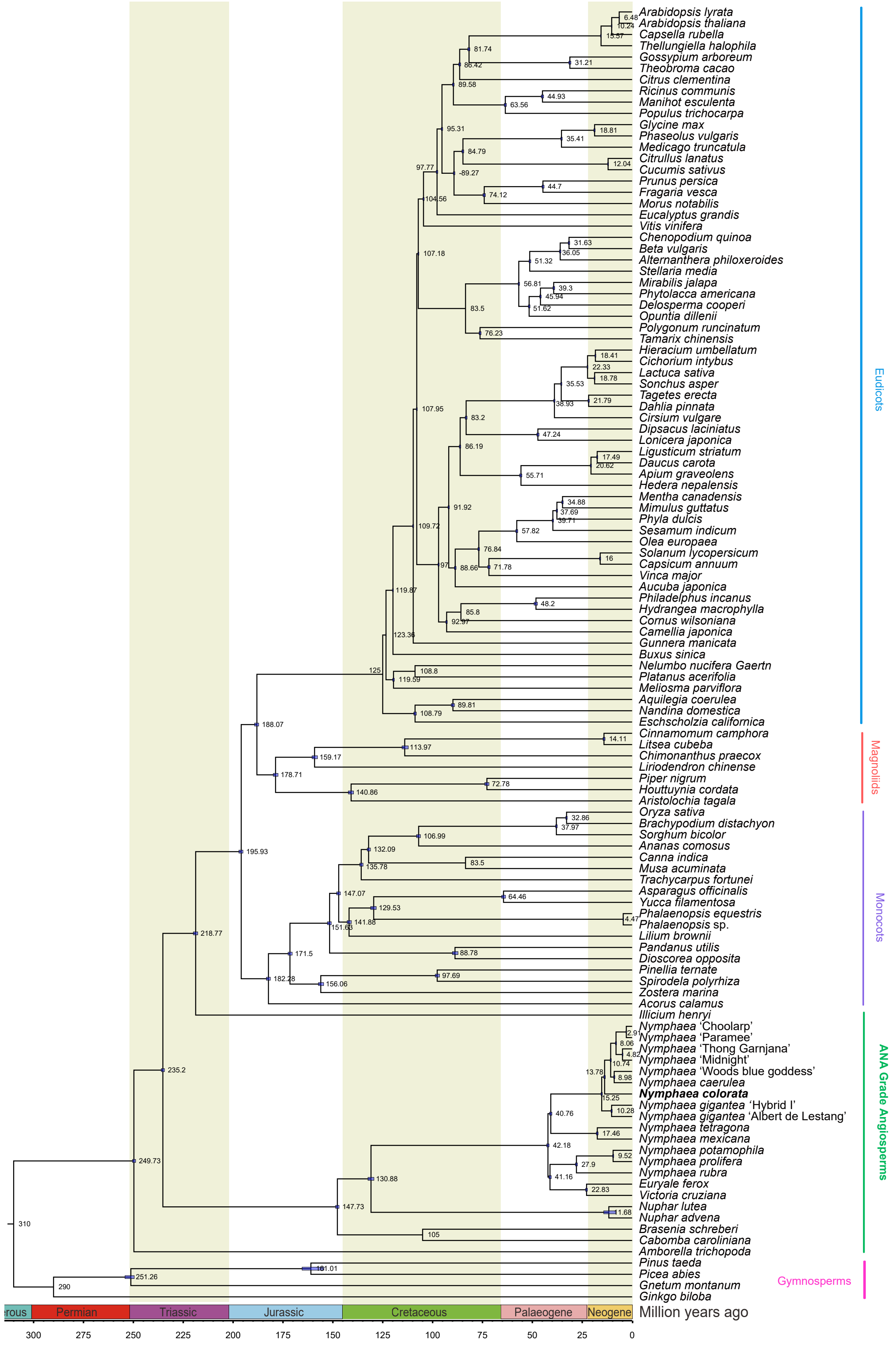

Supplementary Fig. 22 | Estimation of divergence times of seed plants based on penalized likelihood in r8s method. The 95% confidence intervals are shown on each node. The tree topology corresponds to that of **Fig. 1d**.

4.3 Chloroplast gene-based tree of angiosperms

In the tree constructed using 78 chloroplast genes across 361 species, the relationships among *Amborella*, *N. colorata*, and other flowering plants was well resolved. *Amborella* was the earliest divergent extant angiosperm branch, forming a sister group to all other extant flowering plants (Supplementary Figure. 23).

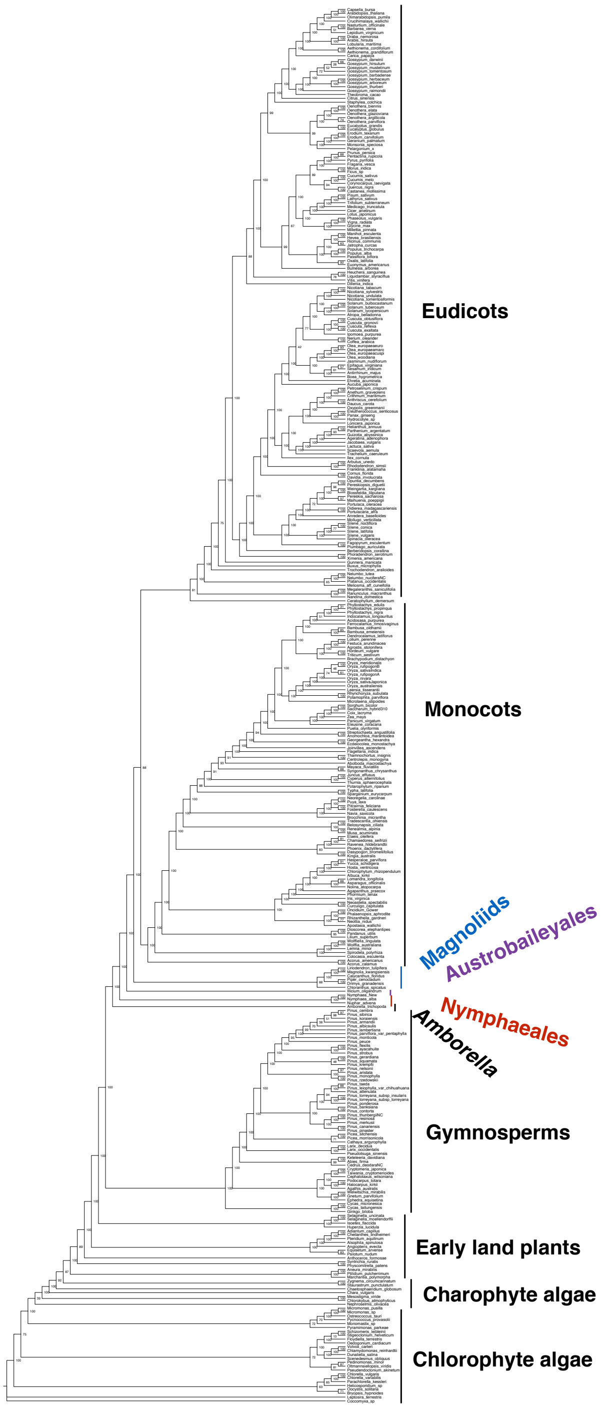

Supplementary Fig. 23| Tree of 361 representative green algae and plants based on 78 chloroplast genes.

4.4 Mitochondrial gene-based tree of angiosperms

The mitochondrial genomes of land plants typically contain 50 to 60 genes[59]. We used 67 plant species and 41 conserved mitochondrial (Mt) gene sets to infer the evolutionary history of flowering plants. In the tree (Supplementary Fig. 24), *N. colorata* formed a sister lineage to all other flowering plants with a relatively low bootstrap value of 67, and *Amborella* was the next sister lineage to the remaining angiosperms, suggesting that the reconstructed evolutionary history of angiosperm mitochondrial genes differs from those of the nuclear genes and the chloroplast genes.

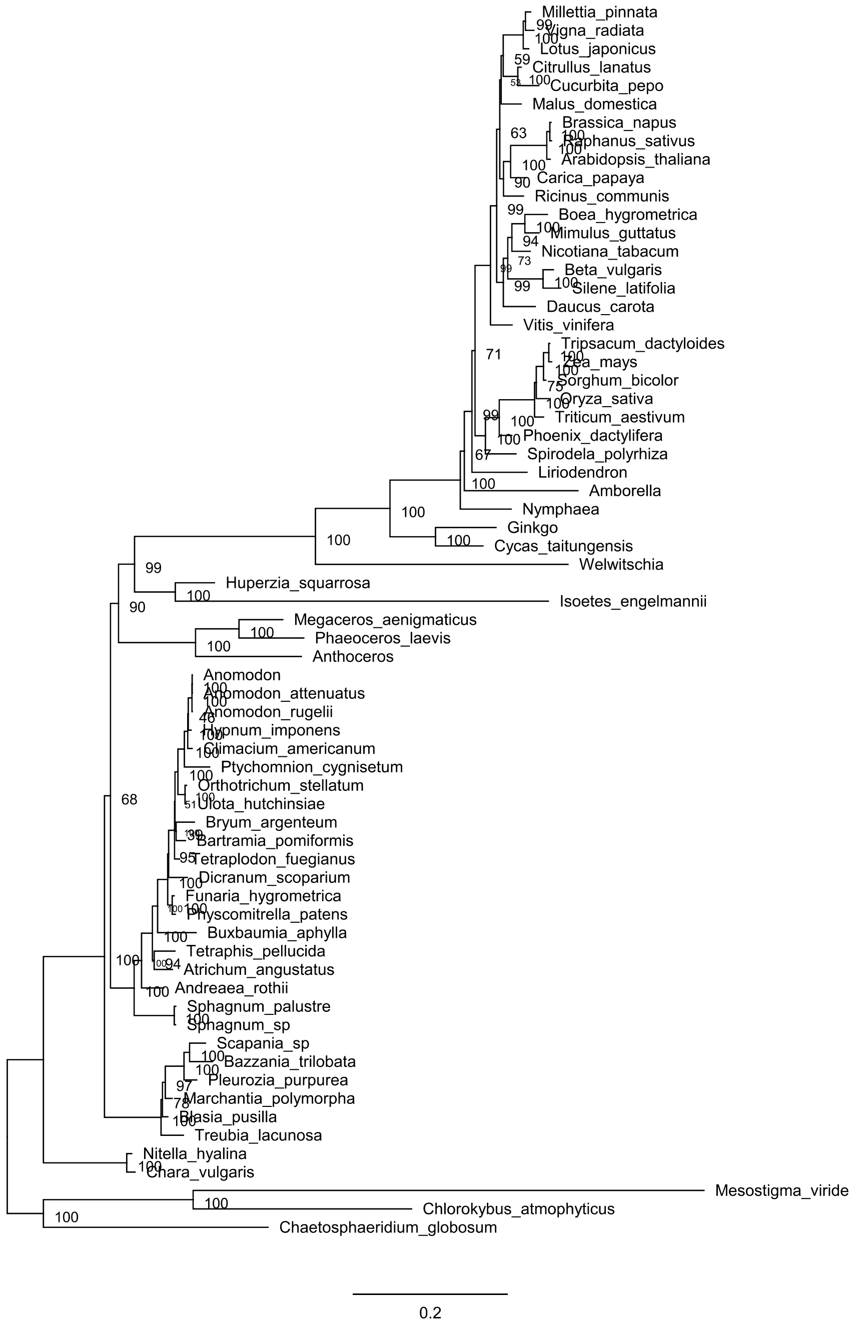

Supplementary Fig. 24 | Species tree inferred using 41 mitochondrial genes from 64 green plants. In this tree, water lily, rather than *Amborella*, is the sister group to all other angiosperm species.

5. Whole-genome duplication in the *N. colorata* genome

5.1 Intra- and inter-genomic collinearity analyses

Genome-wide comparison of gene order finds substantial genomic synteny in *N. colorata* (Extended Data Fig. 1f), with 2,858 gene pairs located in paralogous blocks (Supplementary Table 16). Intergenomic comparisons between *N. colorata* and *Amborella trichopoda*, *Nelumbo nucifera*, and *Vitis vinifera* are consistent with a lineage-specific WGD in *N. colorata* (Extended Data Fig. 2a). For example, *N. colorata* and *Amborella* show a 2:1 syntenic pattern, with two paralogous regions in the genome of *N. colorata* matching one region in the genome of *A. trichopoda*, while *N. colorata* and the eudicot *Nelumbo nucifera* show a 2:2 syntenic pattern and *N. colorata* and the eudicot *V. vinifera* show a 2:3 syntenic pattern, consistent with independent WGDs in the respective lineages of *N. colorata*, *Nelumbo nucifera*, and *V. vitis*[60].

5.2 Analyses of K_S distributions

Distributions of synonymous substitutions per synonymous site (K_S) for paralogues found in collinear regions (anchor pairs) and for the whole paranome of *N. colorata* further support an ancient lineage-specific WGD, both showing a signature peak at $K_S \approx 0.9$ (Extended Data Fig. 2b). Peaks at similar K_S values were identified from the transcriptomes of several other species in the family Nymphaeaceae, but the K_S distribution of *Cabomba caroliniana* in the family Cabombaceae showed no clear peak (Supplementary Fig. 26). Such a pattern of WGD signatures of similar ages across several lineages suggests a single WGD event, possibly shared among (at least) most or all genera in the family Nymphaeaceae.

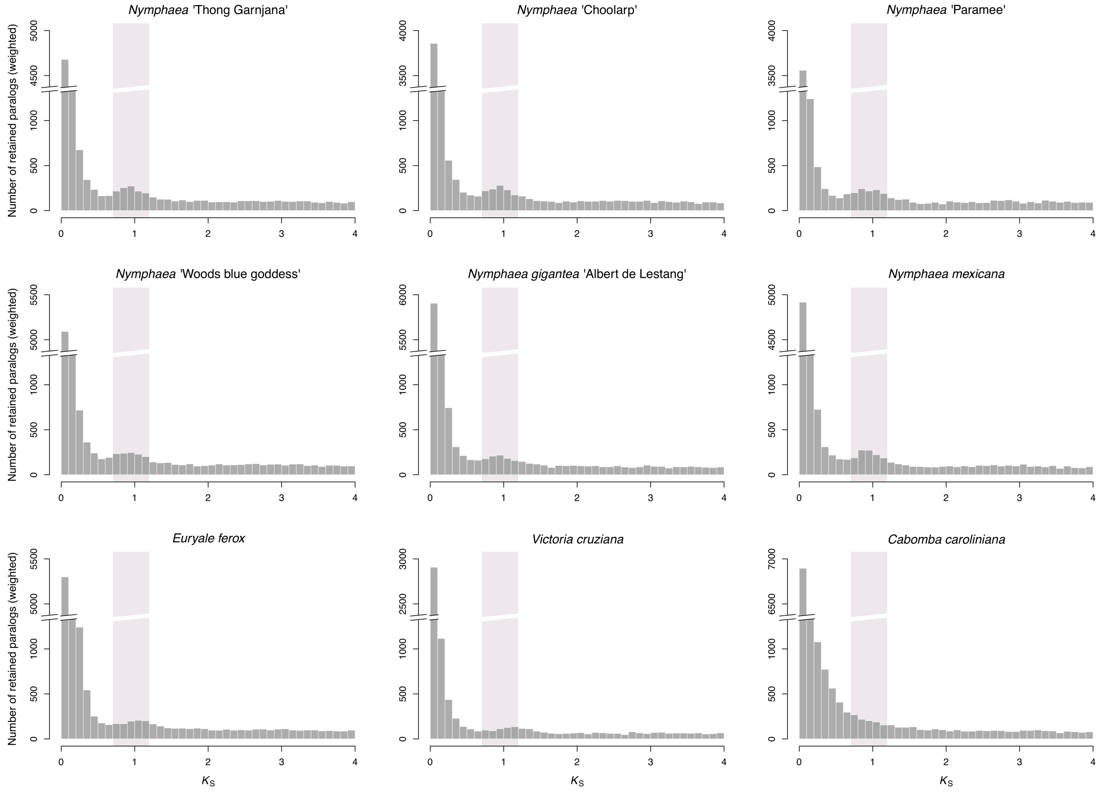

Supplementary Fig. 26 | Distributions of synonymous substitutions per synonymous site (K_S) of the whole paranome for nine Nymphaealean transcriptomes. K_S distributions of paralogues are shown in grey. The light grey rectangle in the background of each plot highlights the K_S range from 0.7–1.2 showing the K_S boundaries used to extract duplicate pairs in *N. colorata* for absolute phylogenomic dating of the WGD event (Extended Data Fig. 2d). Since *C. caroliniana* is a recent polyploid ($2n$ = 104)[8], it is possible that the remnants of an ancient WGD are obscured in the K_S distribution by the presence of many more recently duplicated genes in combination with slightly stronger saturation effects due to the higher substitution rate in *C. caroliniana*.

To further support and better place the potentially shared WGD in the Nymphaeales phylogeny, we compared the anchor-pair K_S distribution of *N. colorata* with K_S distributions of orthologues between *N. colorata* and species from other lineages in Nymphaeales and from ANA-grade angiosperm lineages (**Fig. 2a**). The anchor-pair WGD K_S peak of *N. colorata* is much younger than the K_S peaks of the orthologues between *N. colorata* and *Amborella* and *Illicium henryi* (order Austrobaileyales), further confirming a Nymphaeales-specific event. In contrast, the WGD K_S peak is much older than both the K_S peak for *N. colorata*–*Victoria cruziana* orthologues and the K_S peak for *N. colorata*–*Nuphar advena* orthologues. It is also slightly younger than but possibly overlapping with the K_S peak for *N. colorata*–*C. caroliniana* orthologues. If substitution rates were similar among all of these lineages (and between paralogues and orthologues), our results would suggest that the WGD occurred just after the divergence of *N. colorata* and *C. caroliniana* but prior to the divergence of *N. colorata* and *Nuphar advena*, *i.e.*, in the stem lineage of Nymphaeaceae. However, substitution rates do seem to vary considerably among Nymphaealean lineages (**Fig. 2b** and Extended Data Fig. 2c). Species in the genus *Nuphar* seem to have substantially lower substitution rates than the other lineages in Nymphaeaceae, whereas *C. caroliniana* seems to have a higher substitution

rate than lineages in the sister family Nymphaeaceae. Therefore, the K_S peak value of the *N. colorata–Nuphar advena* orthologues is likely to provide an underestimated age compared with that from the WGD K_S peak of *N. colorata*, whereas the age from the K_S peak value of the *N. colorata–C. caroliniana* orthologues is likely to be a slight overestimate (indicated by the arrows in **Fig. 2a**). Thus, it is possible that the WGD is shared between the families Nymphaeaceae and Cabombaceae and already occurred just prior to their divergence.

5.3 Timing of the WGD in *N. colorata*

Absolute dating of the paralogues of *N. colorata* using phylogenomic methods[61] suggests that the WGD identified in the genome of *N. colorata* occurred approximately 117–98 Ma (Extended Data Fig. 2d; using a set of orthogroups that include orthologues from *Amborella* and *Ginkgo biloba*, see **Methods**). Estimates of the divergence time between Nymphaeaceae and Cabombaceae vary widely, placing it as early as in the Jurassic (185–147 Ma in **Fig. 1d**) or in the lower Cretaceous (127–120 Ma, 95% highest posterior density (HPD)[62]; 117–105 Ma, 95% confidence intervals, CI[63]), or as late as the Eocene (75–38 Ma, 95% CI[64]; 72–16 Ma, 95% HPD[65]; 46–38 Ma, 95% CI[66]). Our absolute estimate for the timing of the WGD seems to overlap with the older estimates in the lower Cretaceous, further suggesting that the WGD could have occurred before or close to the divergence between Nymphaeaceae and Cabombaceae.

In addition, we built a second set of orthogroups for each WGD paralogous pair by waiving the requirement of orthologues from *Amborella* and *G. biloba* from the taxonomic sampling listed in the **Methods**, leading to a separate set of 329 orthogroups based on anchor pairs and 208 orthogroups based on peak-based duplicates. We used all the fossil calibrations as described in the **Methods**, except the fossil calibration used for the root in the previous starting tree. The node uniting the paralogues of *N. colorata* WGD with the eudicots and monocots was calibrated as a new root. Following Markov chain Monte Carlo sampling, we accepted 436 orthogroups and further analysed these as described in the **Methods**, resulting in an alternative timing estimation of the *N. colorata* WGD at approximately 102–93 Ma (Supplementary Fig. 27, earlier than the estimation based on the orthogroups with orthologues from *Amborella* and *G. biloba* in Extended Data Fig. 2d). This date again suggests that the WGD occurred before or close to the divergence between Nymphaeaceae and Cabombaceae (**Fig. 2b** and **c**), or corresponds to the divergence time between Nymphaeaceae and Cabombaceae as indicated in the allopolyploidy scenario (**Fig. 2d**).

It is important to note that a WGD had already been inferred and dated in *Nuphar advena* in a previous study[61]. Based on transcriptome data, a K_S peak was found at $K_S \approx 0.2–0.6$ and its absolute date was estimated at approximately 77–68 Ma. This lower date could suggest this to be a separate or additional independent WGD in *Nuphar*. However, due to the much lower substitution rates in *Nuphar* and considering that only one WGD has been identified in *Nuphar advena* based on the K_S age distribution of paralogues, we suggest that this WGD signature and the signatures in *N. colorata* and other species of Nymphaeaceae all represent the same, single WGD event. In that case, the large difference between the absolute WGD ages

estimated from *Nymphaea colorata* and *Nuphar advena* mirrors the large differences in estimates of deep divergence events within angiosperms.

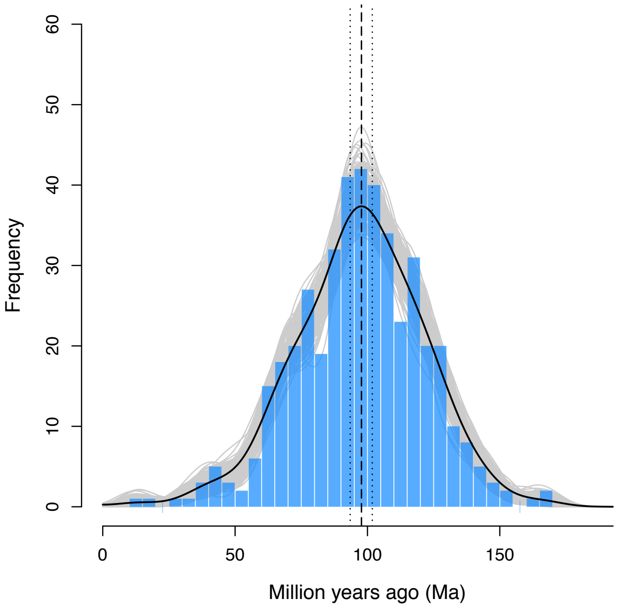

Supplementary Fig. 27 | Absolute age distribution obtained from phylogenomic dating of *Nymphaea colorata* WGD paralogues based on orthogroups without orthologues from *Amborella trichopoda* and *Ginkgo biloba*. The solid black line represents the kernel density estimate of paralogue date estimates, and the vertical dashed black line represents its peak at 98 million years ago (Ma). The grey lines represent density estimates from 2,500 bootstrap replicates, and the vertical black dotted lines represent the corresponding 90% confidence interval for the WGD age estimate, 102–93 Ma (see **Methods**). The blue histogram shows the raw distribution of time estimates for paralogue divergences.

5.4 Phylogenomic analyses of the WGD

To further test whether the WGD occurred before or after the divergence of the two families within Nymphaeales, we analysed gene trees that contained at least one anchor pair from *N. colorata* (see **Methods**). The *N. colorata* anchor pairs in 211 out of 246 gene trees (BS value ⩾ 80%) coalesced on the branch leading to Nymphaeales (**Fig. 2b**). Similarly, the anchor pairs in 216 out of 364 gene trees (BS values ⩾50%) coalesced on the branch leading to Nymphaeales (Supplementary Fig. 28). This would indeed suggest that the WGD occurred already before the divergence of Nymphaeaceae and Cabombaceae. Interestingly, only 28 of the 211 gene trees, gene trees retained both putative WGD duplicates in *C. caroliniana*, far fewer than the duplicates retained in the species in Nymphaeaceae (Supplementary Table 17). This could be true if most duplicates were lost in the lineage to *C. caroliniana* (**Fig. 2c**), which might explain the absence of a clear peak in the K_S distribution of paralogues from this species (Supplementary Fig. 26).

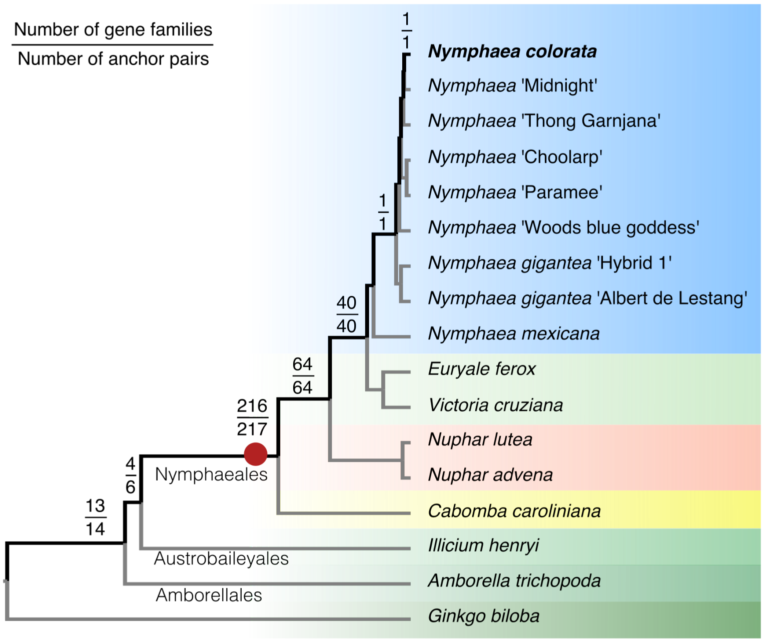

Supplementary Fig. 28 | Phylogenomic analysis of the WGD in Nymphaeales. The numbers on the branches of the species tree indicate the number of gene families with at least one anchor pair from *N. colorata* that coalesced on the respective branch (top) and the actual number of coalesced anchor pairs (bottom). The branch on which most of the anchor pairs in *N. colorata* coalesced is denoted by the red dot. All the duplication events have bootstrap values greater than or equal to 50%.

Alternatively, the absence of a clear K_S peak, the finding of few retained duplicates, and the substantial overlap of the date estimates for both the WGD and the divergence between Nymphaeaceae and Cabombaceae suggest that the above signatures for a shared WGD event could instead be interpreted as an allopolyploidy event that occurred shortly after the divergence between Nymphaeaceae and Cabombaceae ancestors (**Fig. 2d**). The two parental ancestors of such a putative allopolyploid, of which one was more closely related to the ancestor of Cabombaceae than the other, formed a tetraploid hybrid that gave birth to the lineage leading to Nymphaeaceae. In such an allopolyploidization scenario, the anchor pairs of *N. colorata* would coalesce not to the time when the hybridization occurred but to the time when the two parents diverged, *i.e.*, the evolutionary split between Nymphaeaceae and Cabombaceae. The K_S peaks observed in the species of Nymphaeaceae would then reflect the divergence of the two parental ancestors, thus similar to the *N. colorata*–*C. caroliniana* ortholog K_S peak. Because the allopolyploidy event gave rise to the ancestor of the lineage leading to Nymphaeaceae, but not Cabombaceae, species like *C. caroliniana* do not have such a peak in their K_S distributions. The limited number of duplicates in *C. caroliniana* that coalesced prior to the divergence of Nymphaeaceae and Cabombaceae might have resulted from small-scale duplication events. Our absolute phylogenomic dating of the paralogues in *N. colorata* would hence provide support for the divergence of Nymphaeaceae and Cabombaceae in the Early Cretaceous (Extended Data Fig. 2d). Nymphaealean fossils from the Early Cretaceous are consistent with such an early divergence of Nymphaeaceae and Cabombaceae[67]. For example, the fossil *Monetianthus mirus* from the crown group of

Nymphaeaceae is ~113 million years old[68], and the fossil *Scutifolium jordanicum* from the stem group of Cabombaceae is at least 105 million years old[69].

5.5 Pre-angiosperm WGD event

To detect evidence for any pre-angiosperm WGD event, we compared the genomes of the gymnosperm *G. biloba*, *A. trichopoda*, and *N. colorata*. Specifically, we extracted gene pairs inferred to be remnants of such an event and performed detailed analyses using default parameters on a set of matching regions with local synteny and four representative gene trees of syntenic genes. The chromosome synteny was plotted using the JCVI utility libraries (https://github.com/tanghaibao/jcvi) with default parameters.

Similar to the findings from the *Amborella* genome, our comparative analyses identified several duplicated blocks that appeared to predate the divergence of Amborellales and Nymphaeales. Local synteny analyses across *G. biloba*, *Amborella*, and *N. colorata* identified several cases where one *G. biloba* region aligned with up to two *Amborella* regions, supporting a pre-angiosperm WGD (since no lineage-specific WGD was found in *Amborella*); in turn, each *Amborella* region aligned with up to two *N. colorata* regions, supporting the additional WGD in the Nymphaealean lineage. Additionally, consecutive gene trees sampled from the selected regions were consistent with the timing of the WGD events as expected from the local synteny (Supplementary Fig. 29). We identified a total of 244 gene pairs that supported a pre-angiosperm WGD, and these were located in 52 pairs of *Amborella* scaffolds. Similarly, we identified a total of 153 gene pairs in *N. colorata* that might also be derived from this WGD event. The weaker signal in *Nymphaea* for duplicated regions from such older WGD events was most likely due to the presence of the more recent WGD in the Nymphaealean lineage and subsequent fractionations that may have further altered the ancestral gene orders[9].

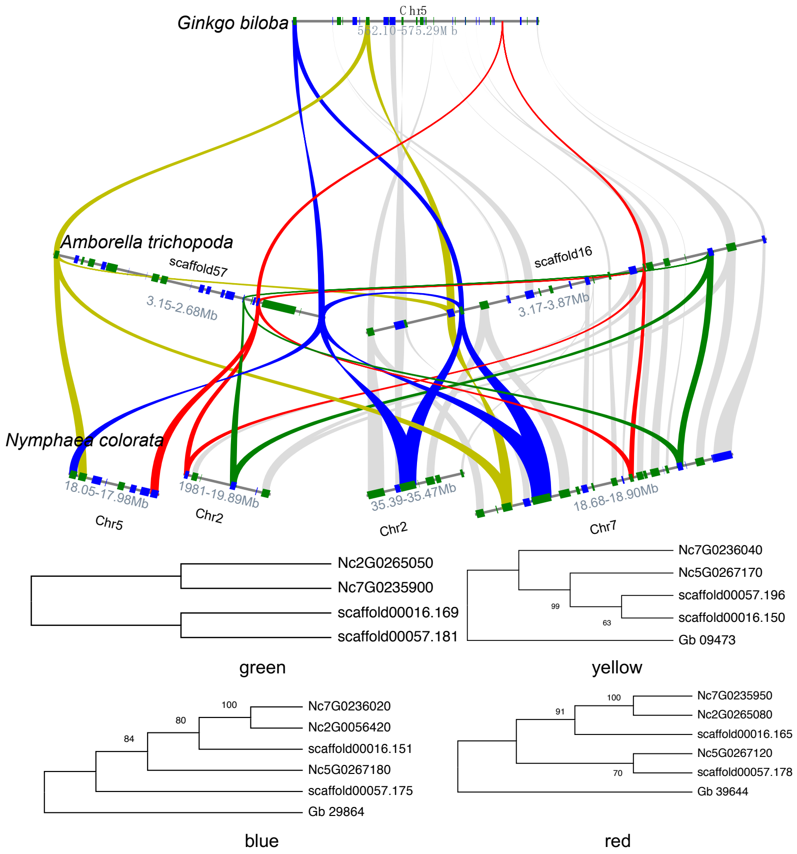

Supplementary Fig. 29 | Exemplar local syntenic regions in support of a pre-angiosperm whole genome duplication (WGD) event. A single *Ginkgo biloba* region is aligned to two *Amborella* regions and four *N. colorata* regions, consistent with a pre-angiosperm WGD event followed by WGD specific to the Nymphaealean lineage. Note that the two regions on *Nymphaea* Chr2 were split as they were aligned consecutively to *Amborella* scaffold16, but they should be treated here as one single syntenic region. Four sets of syntenic gene groups across multiple regions that are inferred to be derived from a pre-angiosperm WGD are shown in different colours; the four reconstructed gene trees shown at the bottom are in the corresponding colours.

6. Genes related to floral development in *N. colorata*

Angiosperm flowers typically have specialized scents, colourful perianths such as petals, and both male and female reproductive organs, unlike gymnosperms, which have unisexually reproductive cones (strobili) with no specialized scent or colour. The flower of *Amborella* (the sole species in Amborellales), however, is simple and either male or female, with sepal or sepal-like perianth organs that lack floral scent, hence lacking many of the characteristics seen in typical angiosperms. In contrast, the flowers of water lilies have diverse scents and colours, and with both male and female organs, similar to the flowers of *Illicium* (a genus from Austrobaileyales). The flowers of mesangiosperms have evolved further diversity in these features, being either fragrant or non-scented, colourful or white, unisexual or bisexual, and possess various additional modifications such as spots, trichomes, and nectaries (**Supplementary Fig. 1**).

6.1 MADS-box transcription factors

MADS-box genes encode eukaryote-specific transcription factors controlling multiple developmental programs[70]. The MADS-box gene family is divided into two types: type I and type II. Type I includes three subfamilies, α, β, and γ, and type II includes MIKC* and MIKC^{c}. The MIKC^{c} genes are the best studied in terms of their expression patterns and associated mutant phenotypes, and their functions in floral organ specification are well characterized. The MIKC^{c} subfamily comprises the following groups: *TM3*/*SOC1*, *TM8*, *AP3*, *PI*, *AGL32*/*GMM13*, *AGL12*, *SEP*, *AP11/FUL*, *ANR1*, *AGL15*, *SVP*, *AG*, *FLC*, *OsMADS32*, *AGL6*, and *STK*. Although this subfamily has been extensively studied, it is still unclear how many type II MIKC^{c} groups evolved in the ancestor of flowering plants or in the ancestor of seed plants[71].

Materials and Methods

To identify *N. colorata* MADS-box genes, we searched the predicted proteome of *N. colorata* using hmmsearch in HMMER[72], based on the seed SRF-TF (PF00319) from the Pfam database[73]. MADS-box classification was based on sequence similarity searches of identified MADS-box genes from *Arabidopsis* and *Amborella*[15]. The obtained results were manually curated, including the concatenation of two *ANR1* genes (NC3G0228930+NC3G0228920, NC9G0169500+NC9G0169520), one *AG* gene (NC9G0111830+NC9G0111840), the *SOC1* gene (NC9G0274620+NC9G0274600), and the *STK* gene (NC7G0291940+NC7G0291950). For evolutionary analysis, we aligned sequences using MAFFT [46] with the parameter E-INS-I (accurate). A phylogenetic tree of MADS-box genes was constructed using FastTree 2.1.10 software[74] and edited in MEGA 6[47].

To characterize the tandem duplicated MADS-box genes, we firstly identified the genome-wide syntenic genes in the genomes of *N. colorata*, *Amborella*, *Nelumbo nucifera*, *Vitis vinifera*, and *Spirodela polyrhiza* using MCScanX[75] with default parameter settings. The putative tandem duplicated MADS-box genes were manually checked based on their genomic location information.

Results and Discussion

Nymphaeales not only occupies an important evolutionary position in the angiosperm lineage but also has several key floral characteristics, such as the presence of multiple floral organs, similar to magnoliids, and the presence of both stamens and carpels in the same flower. The latter feature is similar to most angiosperms but different from *Amborella* and gymnosperms. As a member of Nymphaeales, *N. colorata* represents an excellent genomic resource for investigating the early evolution of floral developmental programs, especially for comparison with eudicot and monocot species. Similar to *Illicium* and *Magnolia*, *N. colorata* has somewhat differentiated sepals and petals.

N. colorata encodes 70 MADS-box genes, in contrast to the 33 MADS-box genes in *Amborella trichopoda.* Some Type I MADS-box genes are critical for endosperm development initiation[76]. Expansion of Type I Mα genes (31 paralogues) was observed in *N. colorata*, compared to six paralogues in *Amborella*. The known functions of Type I genes in female gametophyte development[77] suggest that their expansion in *N. colorata* might have contributed to larger numbers of ovules, resulting in abundant seed production. *N. colorata* and *Amborella* both had two MIKC* group members.

NcMIKC^c consists of 15 groups, including *AG*, *STK*, *AGL12*, *TM3/SOC1*, AGL6, *SEP1*, *FLC*, *AP1/FUL*, *AGL15*, *ANR1*, *AP3*, *PI*, *AGL32*, *SVP*, and *OsMADS32* (Supplementary Fig. 30). Note that *SEP3* and *TM8* were not found in the *N. colorata* genome, nor were they present in any of the 20 transcriptomes of water lilies. Since genes of the *AP1*/*FUL* group are present in *Amborella* and *FLC* is a sister group to *AP1*/*FUL, FLC* was likely present in the last common ancestor of angiosperms and subsequently lost or not found in *Amborella*. In addition, two *FLC* homologues were found in the *N. colorata* genome on syntenic chromosome regions (**S**upplementary Fig. 30), likely resulting from the Nymphaealean WGD.

Besides the evolutionary history as shown in Supplementary Figures 31-34, we further investigated the expression profiles of the Type II–MIKC^c genes in the vegetative and floral organs of *N. colorata* (**Fig. 3**). Following the Nymphaealean WGD, both duplicates of the C-function genes *AGa* and *AGb,* were retained on collinear blocks (Extended Data Fig. 4c). *AGb* is expressed in all floral organs, while *AGa* is mainly expressed in the stamens and carpels, suggesting that *AGa* has gained specialized function in these organs. Moreover, the expansion of the *AG* genes due to the WGD and their differential expression patterns might have contributed to the increased number of stamens and carpels in Nymphaeaceae.

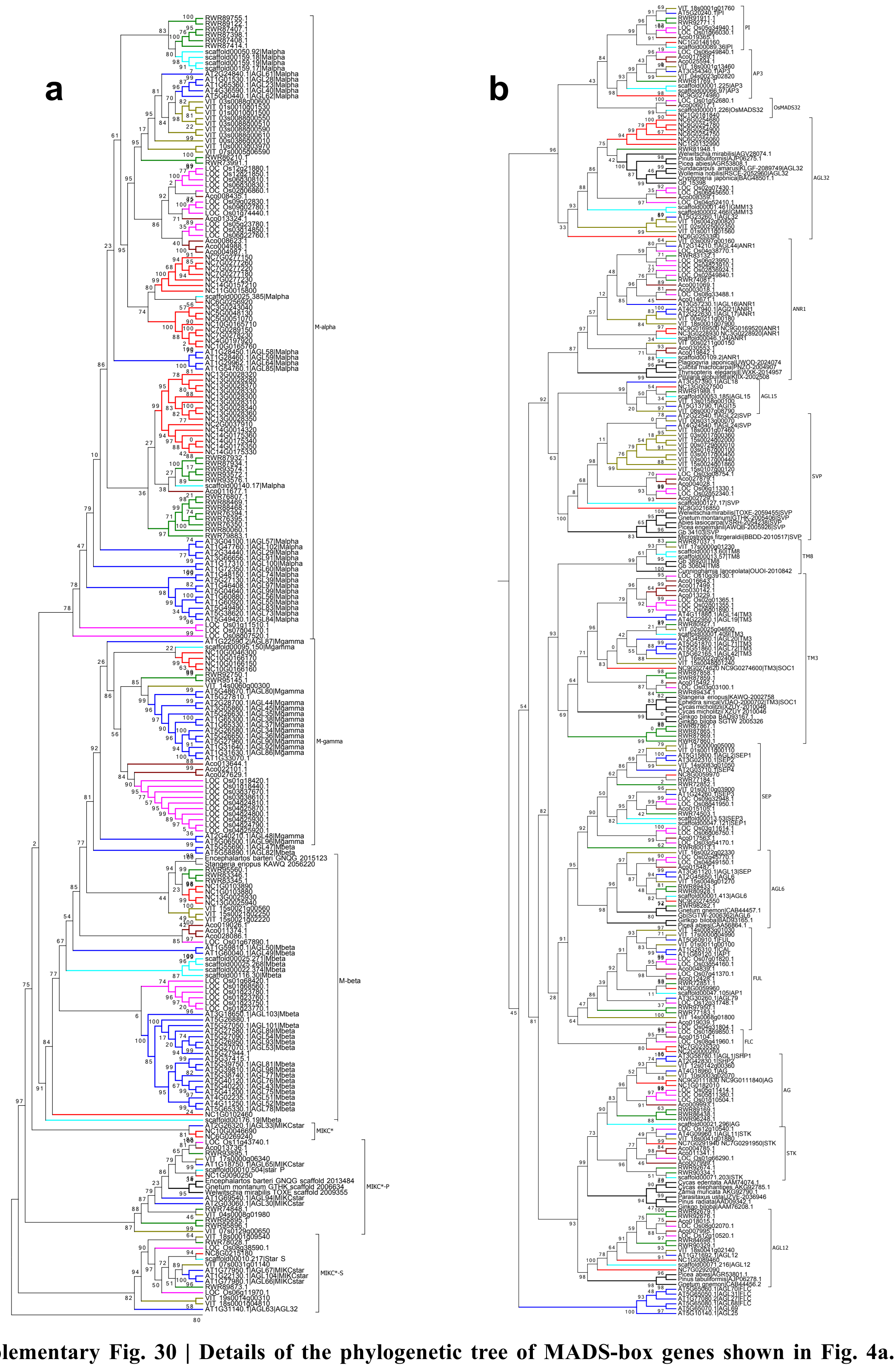

Supplementary Fig. 30 | Details of the phylogenetic tree of MADS-box genes shown in Fig. 4a. Note that the NC2G000030 was not shown due to its poor gene length. The sampled angiosperm species include *Arabidopsis thaliana* (gene symbols start with AT), *Vitis vinifera* (VIT), *Cinnamomum kanehirae* (RWR), *Oryza sativa* (LOC), *Ananas comosus* (Aco), *Nymphaea colorata* (Nc), *Amborella trichopoda* (scaffold).

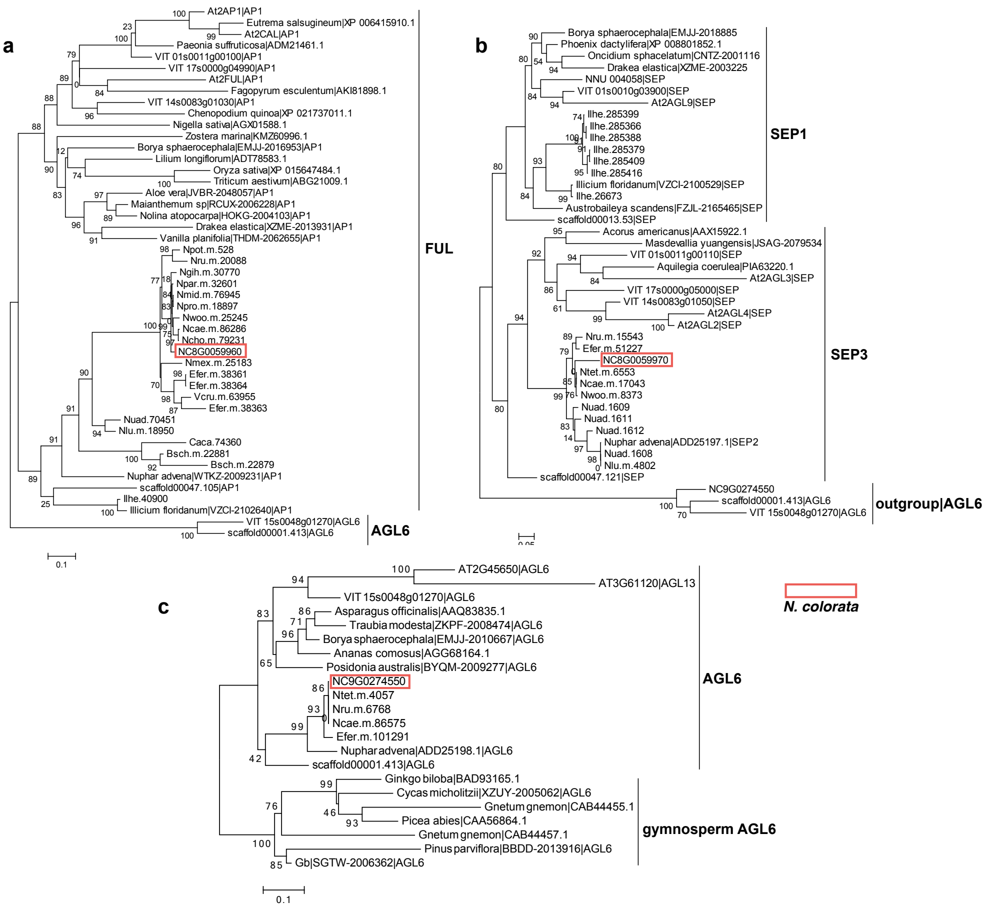

Supplementary Fig. 31 | Details for the A- and E-function MADS-box genes and close relatives. (a) The phylogenetic tree of AP1 from *Nymphaea colorata* and other representative seed plants. **(b)** The phylogenetic tree of SEP from *N. colorata* and other representative seed plants. **(c)** The phylogenetic tree of AGL6 from *N. colorata* and other representative seed plants.

Water lily has several copies of *AGL32*, also called B-sister genes for their close relationship to the B-function genes *AP3* and *PI* (Supplementary Fig. 32). Intriguingly, one of the *AGL32* homologues (NC6G0254900) is expressed in petals at a relatively high level, while another homologue (NC6N0254780) has a higher level of expression in stamens than in other floral organs (**Fig. 3a**), suggesting that they contribute to B-function and might have experienced subfunctionalization for petal and stamen development, respectively.

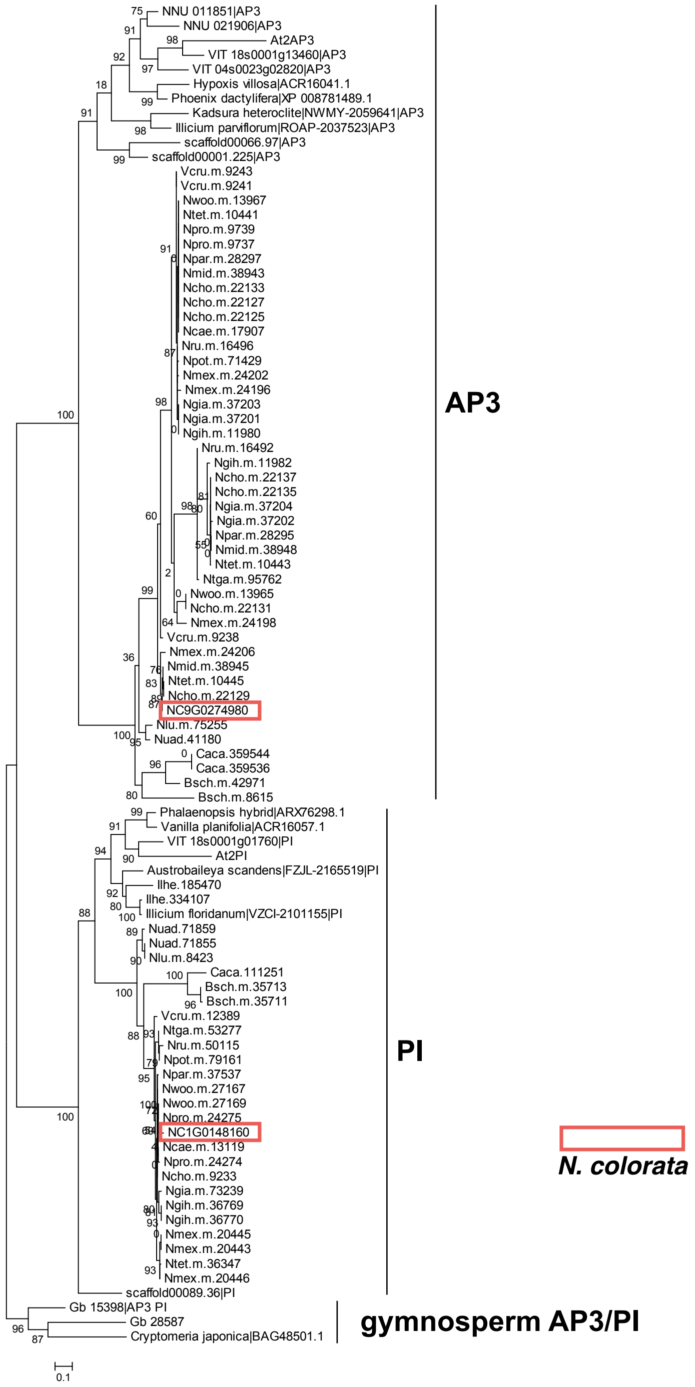

Supplementary Fig. 32 | Details for the B-function and B-sister MADS-box genes. The phylogenetic tree of AP3-PI from *Nymphaea colorata* and other representative seed plants.

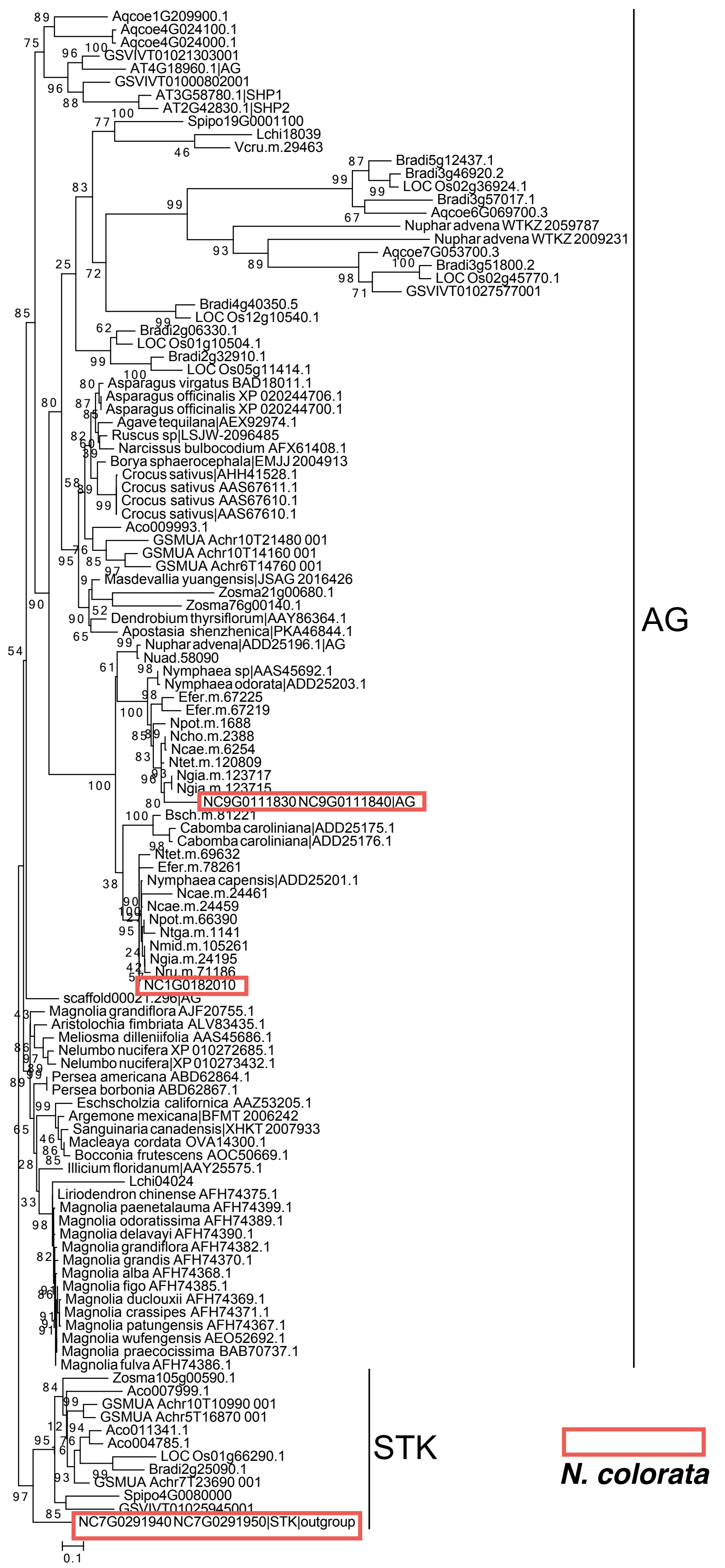

Supplementary Fig. 33 | Details for the C-function MADS-box genes and close relatives. The phylogenetic tree of AG-STK sequences from *Nymphaea colorata* and other representative seed plants.

The MADS-box gene tree that includes homologues from several seed plants shows that *AGL6, SEP1/SEP3* and *AP1/FUL* belong to the same large clade (Supplementary Fig. 30). In addition, the high-quality chromosome assembly of *N. colorata* allows the detection of the tandem gene array of *AP1/FUL* and *SEP1* by comparison among *N. colorata*, *Amborella*, the monocot *Spirodela polyrhiza*, and the eudicot *Vitis vinifera*. Given that gymnosperm orthologues for *AGL6* clustered with *SEP1*, our results indicate that *AP1/FUL* and *SEP1* originated from an ancient tandem duplication event prior to the divergence of seed plants (Extended Data Fig. 3b). The two duplicate genes were then retained in the extant angiosperms and eventually resulted in the A function gene (*AP1/FUL*) for the specification of sepals and petals, and E function genes (*SEP*) that encode proteins interacting with ABC function proteins to determine floral organ identity[78].

6.2 Expansion of genes regulating the morphogenesis of male organs and female organs in Nymphaeales

In *Arabidopsis*, genes involved in meristem size and maintenance include the following[79]: *ARGONAUTE1* (*AGO1*), *TESMIN/TSO1-like* (*TSO1*), *CLAVATA2* (*CLV2*), *CA^{2+}-DEPENDENT NUCLEASE* (*CAN*), *FASCICLIN* (*FAS*), *HANABA/MONOPOLE* (*HAN/MNP*), *SHOOT MERISTEMLESS* (*STM*), *ULTRAPETALA1* (*ULT1*), *ULTRAPETALA2* (*ULT2*), *UNUSUAL FLORAL ORGANS* (*UFO*), and *WIGGUM/ENHANCED RESPONSE TO ABA 1* (*WIG/ERA1*). Genes involved in organ boundary establishment include *CUP-SHAPED COTYLEDON1/2* (*CUC1/CUC2*), *CUC3*, and *SUPERMAN/FLORAL ORGAN NUMBER 1* (*SUP/ FON1*). A non ABC-gene involved in organ type specification and identity determination is *LEUNIG/ROTUNDA 2* (*LUG/RON2*).

Genes involved in floral meristem and primordia (and organ) polarity establishment include *ABONORMAL FLORAL ORGANS* (*AFO*), *ASYMMETRIC LEAVES 1/2* (*AS1/AS2*), *JAGGED* (*JAG*), *JAGGED LATERAL ORGANS* (*JLO*), *KANADI 1,2,3,4* (*KAN1,2,3,4*), *NUBBIN/JAGGED-LIKE* (*NUB/JAG*), *PHABULOSA* (*PHB*), *PHAVOLUTA* (*PHV*), *YABBY3* (*YAB3*), *AINTEGUMENTA* (*ANT*), *ETTIN/AUXIN RESPONSE TRANSCRIPTION FACTOR 3* (*ETT/ARF3*), *P-GLYCOPROTEIN 19* (*PGP19*), *PERIANTHIA* (*PAN*), *PETAL LOSS* (*PTL*), *PIN-FORMED 1,3,7* (*PIN1,3,7*), *PINOID* (PID), and *TOUSLED* (*TSL*).

Genes involved in floral organ morphogenesis include *EARLY BOLTING IN SHORT DAYS* (*EBS*), *FRILL1/STEROL METHYLTRANSFERASE 2* (*FRL1/SMT2*), *NAC-LIKE, ACTIVATED BY AP3/PI* (*NAP*), *RABBIT EARS* (*RBE*), CC-type glutaredoxin (also named *ROXY1*), *SPOROCYTELESS/NOZZLE* (*SPL/NZZ*), *STERILE APETALA* (*SAP*), and *STRUBBELIG-RECEPTOR* (*SUB*).

Genes mainly involved in stamen development include *ABORTED MICROSPORES* (*AMS*), *AUXIN RESPONSE TRANSCRIPTION FACTOR6,8* (*ARF6,8*), *BETA-AMYLASE1,2,3* (*BAM1,2,3*), *CORONATINE INSENSITIVE 1* (*COI1*), *DEFECTIVE ANTHER DEHISCENCE 1* (*DAD1*), *DELAYED DEHISCENCE 1/OXOPHYTODIENOATE-REDUCTASE 3* (*DDE1/OPR3*), *DYSFUNCTIONAL TAPETUM 1* (*DYT1*), *EXCESS MICROSPOROCYTES1/EXCESS MICROSPOROCYTES1* (*EXS/EMS1*), *FATTY ACID DESATURASE3,7,8* (*FAD3,7,8*), *GA INSENSITIVE DWARF1* (*GID1*), *MALE STERILITY 1* (*MS1*), *MYB DOMAIN PROTEIN 36*

(*MYB36*), *MEIOSIS DEFECTIVE 1/MULTIPLE CHLOROPLAST DIVISION SITE 1* (*MEI1/MCD1*), *MYB108*, *NAC SECONDARY WALL THICKENING PROMOTING FACTOR1,2,3* (*NST1,2,3*), *MALE-STERILE 5* (*MS5*), *RECEPTOR-LIKE PROTEIN KINASE 2* (*RPK2*), *REPRESSOR OF GA* (*RGA*), and CC-type glutaredoxin (also named *ROXY2*).

Genes mainly involved in carpel development include *SWITCH1* (*SWI1*), *ALCATRAZ* (*ALC*), *LEUNIG_HOMOLOG* (*LUH*), *SEUSS* (*SEU*), *BELL 1* (*BEL1*), *BREVIPEDICELLUS*/*KNOTTED-LIKE FROM ARABIDOPSIS THALIANA* (*BP/KNAT1*), *CRABS CLAW* (*CRC*), *DETERMINATE*, *INFERTILE 1* (*DIF1*), *HECATE 1* (*HEC1,2*), *HUELLENLOS* (*HLL*), *INDEHISCENT* (*IND*), *INNER NO OUTER*/*YABBY* (*INO/YAB4*), *KNOTTED-LIKE FROM ARABIDOPSIS THALIANA 2* (*KNAT2*), *NGATHA1,2,3,4* (*NGA1,2,3,4*), *NO TRANSMITTING TRACT* (*NTT*), *REPLUMLESS* (*RPL*), *SHORT INTEGUMENTS 1* (*SIN1*), *SPATULA*/*ALCATRAZ* (*SPT/ALC*), and *STYLISH 1,2* (*STY1,2*).

Other genes involved in organ growth include *BIG BROTHER* (*BB*) and *HAWAIIAN SKIRT* (*HWS*). Seed alignment sequences of the protein families were downloaded from the Pfam database (http://pfam.xfam.org). We used hmmsearch or BLAST to identify the family members. All of the retrieved family members were curated using phylogenetic analysis to remove unrelated sequences and to identify close homologues to the *Arabidopsis* genes (Supplementary Figs 34-35, Supplementary Table 19).

Results and Discussion

A number of putative stamen genes were duplicated (Extended Data Figs 4-5), including: CCxC/S-type GRXs (named ROXYs), *DYSFUNCTIONAL TAPETUM 1* (*DYT1*), *NAC SECONDARY WALL THICKENING PROMOTING FACTOR* (*NST*), *RESTORATION ON GROWTH ON AMMONIA* (*RGA*), *BARELY ANY MERISTEM 1/2* (*BAM1/2*), *BAM3*, *CORONATINE INSENSITIVE*1 (*COI1*), *RECEPTOR-LIKE PROTEIN KINASE* (*RKP2*). Similarly, duplicated genes for carpel development include *KNOTTED-LIKE FROM ARABIDOPSIS THALIANA* (*KNAT*), *SWITCH1* (*SWI1*), *STYLISH* (*STY*) 1, *SEUSS* (*SEU*), *SEUSS-LIKE* 2 (*SLK2*), *LEUNIG* (*LUG*), *LEUNIG_HOMOLOG* (*LUH*), *REPLUMLESS* (*RPL*), *YABBY* (*YAB*), *NGATHA1* (*NGA1*), *NO TRANSMITTING TRACT* (*NTT*), and *HALF FILLED* (*HAF*). Approximately half of these duplicates are located in syntenic blocks (Supplementary Figs 34-35, Supplementary Table 19), indicating that these duplicates resulted from the Nymphaealean WGD event. Retention of these duplicates suggests that this event might have played an important role in the evolution and development of carpel and stamen in water lilies.

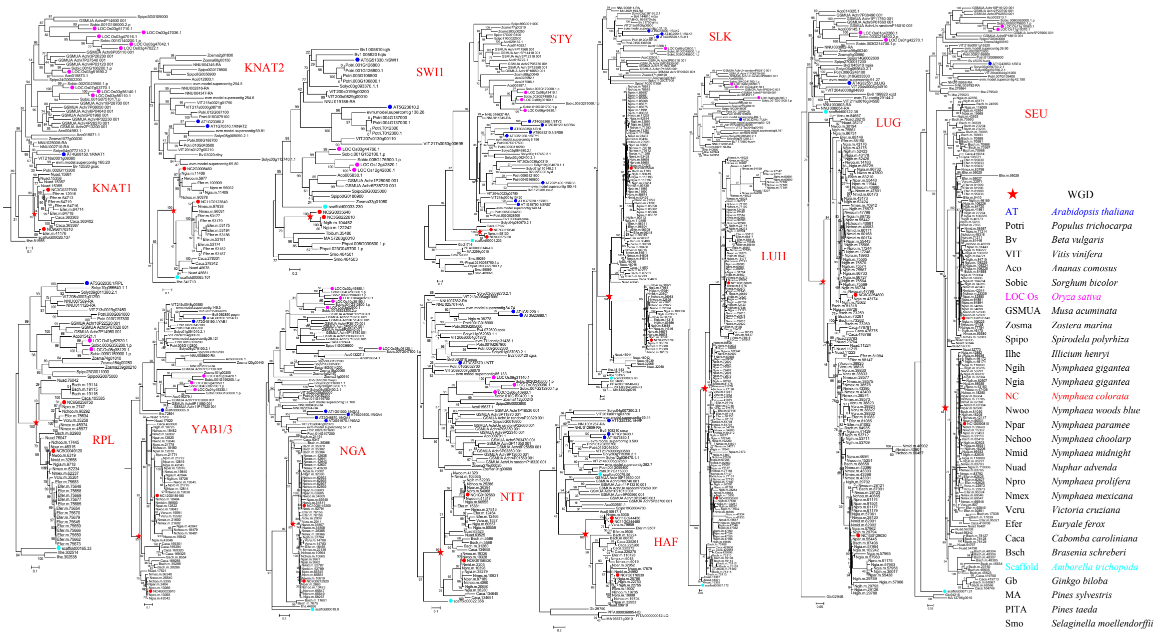

Supplementary Fig. 34 | Genes involved in carpel development had two copies that evolved from the Nymphaealean WGD. The regulatory pathways of these genes have been described in Extended Data Fig. 4**.** *KNOTTED-LIKE FROM ARABIDOPSIS THALIANA 1* (*KNAT1*) encodes a homeobox protein from the class I *KNOX* family of transcriptional regulators. *KNAT1* ensures proper development of pedicels, inflorescence internodes, and carpels. *KNAT2* plays a role in the activation of carpel development regulators, independent of *AG*. *STY* is a member of the *SHI* gene family and encodes a protein with a RING finger-like zinc finger motif. *STY* is important for proper development of both the style and the stigma as well as the vascular system of the gynoecium. *REPLUMLESS* (*RPL*) encodes a homeodomain transcription factor involved in organ identity specification through the repression of *AG* expression in the first two whorls of organs. *NO TRANSMITTING TRACT* (*NTT*) encodes a C2H2/C2HC zinc finger transcription factor specifically expressed in the transmitting tract and involved in transmitting tract development and pollen tube growth. *NGATHA1* (*NGA1*) encodes an AP2/B3-like transcription factor family protein and mainly participates in style and stigma development (at least in part) by mediating auxin synthesis in the apical region of the gynoecium.

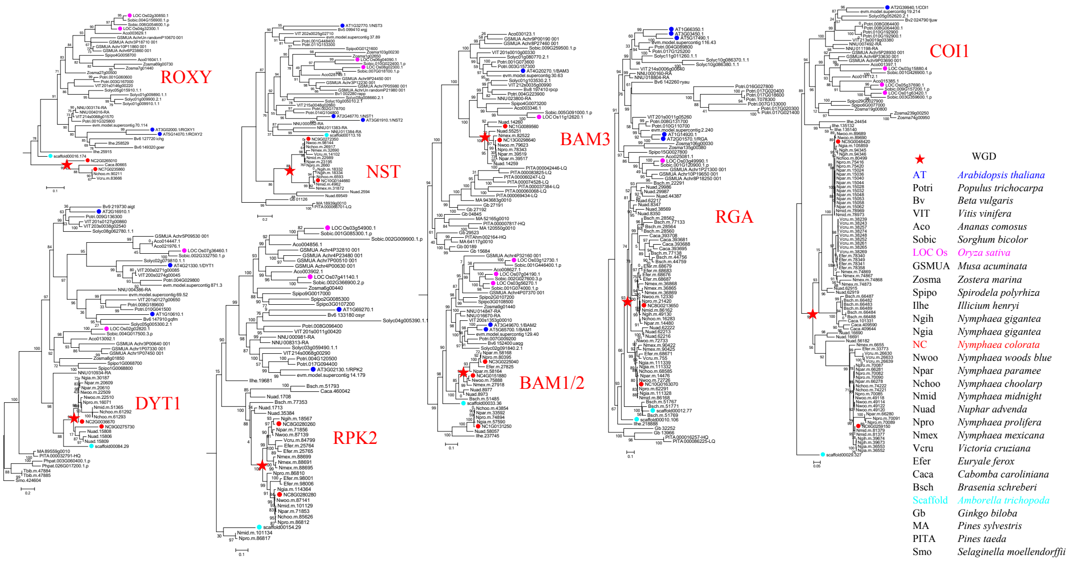

Supplementary Fig. 35 | Genes involved in stamen development had two copies evolved from the Nymphaealean WGD. The regulatory pathways of these genes have been described in Extended Data Fig. 5. *BARELY ANY MERISTEM 1/2/3* (*BAM1/2/3*) encode receptor-like kinases that regulate early anther development. *SWITCH1* (*SWI1*) encodes a novel protein involved in sister chromatid cohesion and meiotic chromosome organization during both male and female meiosis. *DYSFUNCTIONAL TAPETUM 1* (*DYT1*) encodes a bHLH transcription factor strongly expressed in the tapetum from late anther stage 5 to early stage 6 and at a lower level in meiocytes in *Arabidopsis*. The *dyt1* mutant exhibits abnormal anther morphology beginning at anther stage 4 in *Arabidopsis*. *RECEPTOR-LIKE PROTEIN KINASE 2* (*RPK2*) encodes a leucine-rich repeat receptor-like kinase that regulates anther development, tapetal function, and middle layer differentiation. *NAC* transcription factors (*NST1*, *NST2*, and *NST3*) positively regulate the secondary thickening of walls. *NST1* acts in the anther endothecium, the replum margin, and the endocarp b layer of the valve. *NST2* acts in the anther endothecium and is partially redundant with *NST1*. *NST3* acts in the replum margin and in the endocarp b layer of the valve and is partially redundant with *NST1*. *CORONATINE INSENSITIVE 1* (*COI1*) may recruit regulators of pollen development for modification by ubiquitination. It is needed in the JA response, which regulates defence against some pathogens, wound healing, and pollen fertility. **h**, *REPRESSOR OF GA* (*RGA*) encodes a transcriptional repressor of the homeotic genes *AP3*, *PI*, and *AG*. **i**, *ROXY1* and *ROXY2* are CC-type glutaredoxin genes. *ROXY1* is involved in petal initiation in a position-dependent mode rather than an organ-dependent mode. *ROXY1* influences the temporal and spatial expression of *AG* by restricting it to the 3rd and 4th whorls. Together with *ROXY2*, it controls anther development.

6.3 Expansion of genes regulating the floral induction network

Genes regulating floral induction have also been extensively documented, particularly in *Arabidopsis* and rice (*Oryza sativa*)[80]; however, they remain understudied in early-diverging lineages of angiosperms. Homologues of the *Flowering Locus T* (*FT*), which control the flowering transition[81], have expanded to five members in *N. colorata*, through the

Nymphaealean WGD as well as tandem duplications. In contrast, there is only one *FT* gene in *Amborella* (Extended Data Fig. 6a). Both *GIGANTEA* (*GI*) and *CONSTANS* (*CO*) promote flowering under long days and *GI* enhances the expression of both *FT* and *CO*[26,27]. *GI* is relatively conserved in copy number between eudicots and monocots, and has only one member in *Amborella*, yet three *GI* homologues were found in *N. colorata* (Extended Data Fig. 6b). There are also two copies of *CO* in *N. colorata* (Extended Data Fig. 6c), compared with one in *Amborella.*

It is worth noting that *N. colorata* is an ever-blooming plant that continues to flower even when the temperature drops to lower than 18 °C. *Flowering Locus C* (*FLC*) homologues are important repressors of flowering controlled by prolonged cold or vernalization, thus affecting length of crop growth and yields[82]. *FLC* genes were previously only identified in monocots and eudicots, but not found in *Amborella*[82]. Here, we identified two *FLC* homologues in *N. colorata* that originated from the Nymphaealean WGD (Extended Data Fig. 6d). Both *FLC* homologues show expression in vegetative organs and floral organs (Fig. 3). These findings might suggest that the *FLC*-mediated floral repression might have already been present in the ancestor of extant angiosperms, but lost in *Amborella*. The *FLC*, *FT*, *GI*, and *CO* genes are in the same pathway regulating flowering time (Extended Data Fig. 6e), and their duplicates originating from the Nymphaealean WGD have all been retained, potentially contributing to the long-lasting blooming in *N. colorata*.

6.4 Expansion of auxin-related genes and the regulation of floral opening and closure in *N. colorata*

The emission of fragrant molecules in *N. colorata* is controlled by floral diurnal opening and nocturnal closure, which is tightly regulated by the circadian clock, which is in turn controlled by auxin pathways shown in our previous studies[83]. We found significant expansion of auxin-related gene families, such as GLYCOSIDE HYDROLASE3 (GH3), in the genome of *N. colorata* (36 members), compared with only 6 members in *Amborella*, 14 in *Oryza sativa*, 16 in *Nelumbo nucifera*, and 9 in *Vitis vinifera* (Supplementary Fig. 36). The copy number of auxin-inducible genes (*SMALL AUXIN-UPREGULATED*, *SAUR*) in *N. colorata* (62 members) is, in general, higher than those in *Amborella* (30 members) and the gymnosperm *Ginkgo biloba* (48 members), and comparable to that in monocots but lower than that in eudicots (Supplementary Fig. 37).

In addition, we measured the expression of *GH3* and *SAUR* genes in *N. colorata* during the circadian cycle. The expression profiles of auxin-inducible genes at five time points throughout the day (6:00 am, 7:00 am, 10:00 am, 2:00 pm, and 4:00 pm) were measured. Several genes were found to be maximally expressed at 10:00 am, when the opening of the *N. colorata* flower is at its largest angle. The following genes may contribute to circadian regulation of floral opening and closure: NC2G0004540, NC2G0004520, NC2G0286630, NC2G0286700, NC2G0286680, NC2G0286660, NC2G0286610, NC2G0004510, NC2G0286650, NC2G0286640, and NC2G0286720. Surprisingly, we annotated a super cluster consisting of 18 auxin-responsive genes, and seven of the 18 genes responded to the circadian clock and had maximal expression at 10:00 am. Most of the genes showed highest

expression at 10:00 am when the flower is open with the widest angle, whereas some are expressed most highly at 4:00 pm when the flower is closed (Supplementary Fig. 37). The expansion in copy number of these two gene families might play a role in the regulation of cell elongation and promotion of flower opening, similar to the observations in sunflower[84].

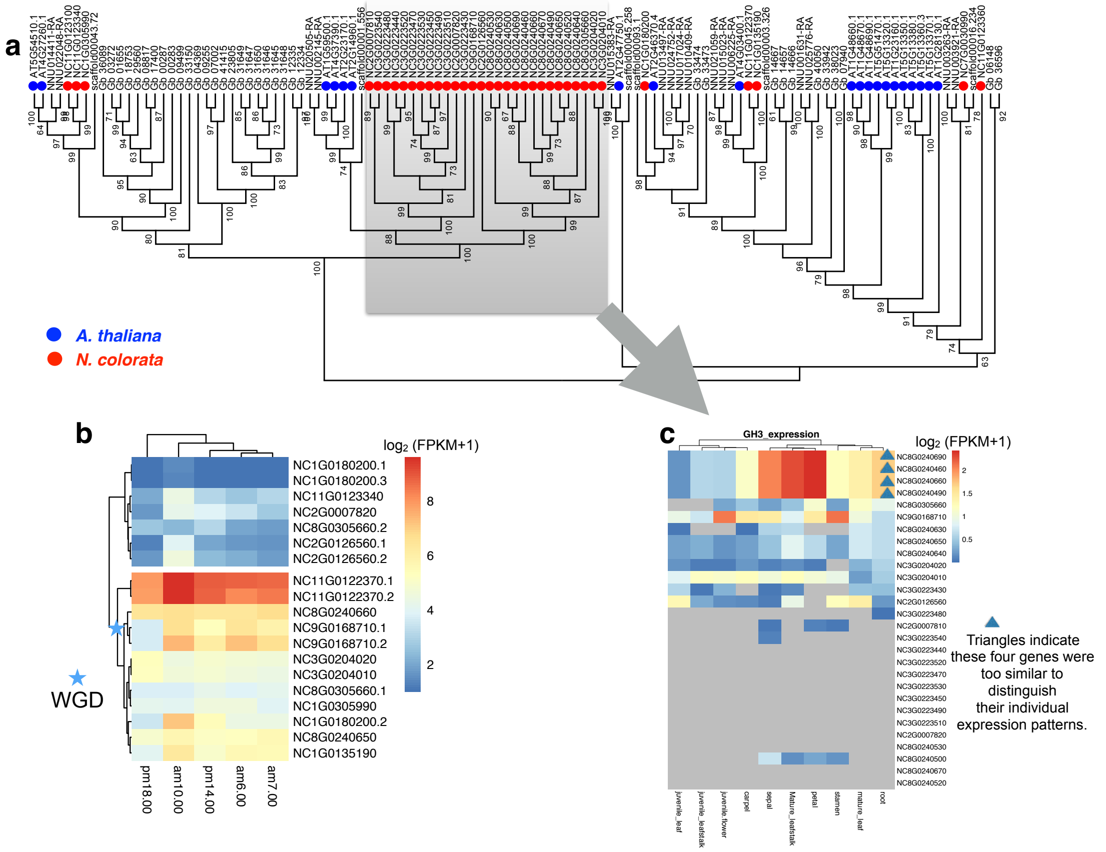

Supplementary Fig. 36 | The expansion of *GH3* genes may be correlated with the elongated stem and frequent opening and closure of the *Nymphaea colorata* flower. a, Expansion of *GH3* as shown in the phylogenetic tree. The *GH3* gene family in angiosperms was divided into five groups. A cluster in *N. colorata* expanded significantly to 29 members. **b**, The diurnal expression patterns of *GH3* genes in the *N. colorata* flower. The blue star indicates the Nymphaealean WGD event. **c**, The *GH3* gene cluster on chromosome 8 (NC8G0240690, NC8G0240460, NC8G0240660, and NC8G0240490) contains auxin-responsive genes that are expressed significantly in the mature leafstalk as well as the sepal and petal floral organs, suggesting that they may regulate the flower's behaviour of reaching out of water and floral opening and closure.

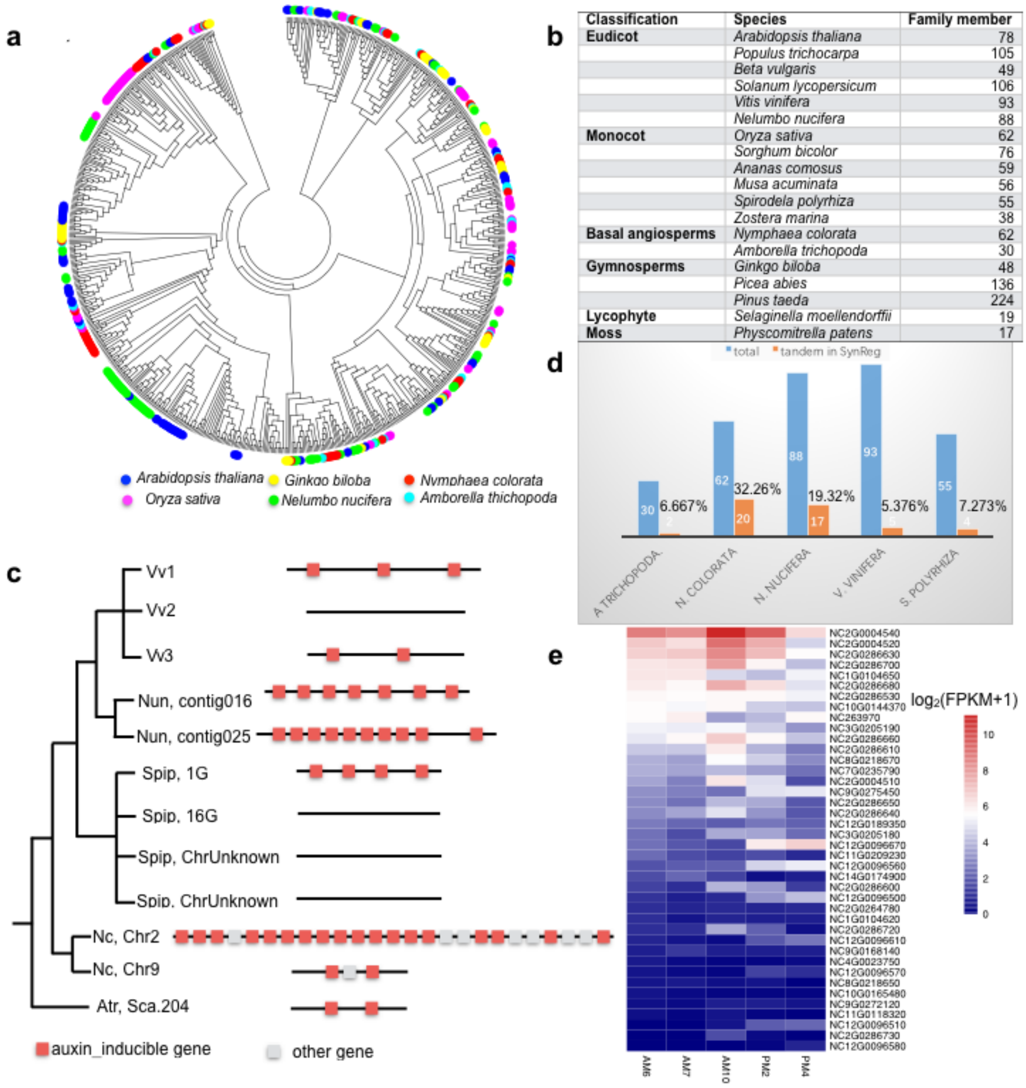

Supplementary Fig. 37 | The auxin-inducible gene family (Pfam ID: PF02519) was expanded in the *Nymphaea colorata* genome. **a**, Phylogenetic tree of the auxin-inducible gene family in representative seed plants. The numbers of auxin-inducible genes in each species are as follows: *N. colorata* (62); *A. thaliana* (78); *N. nucifera* (88); *O. sativa* (62); *G. biloba* (48); *A. trichopoda* (30). **b**, Gene family size across representative land plants. **c**, Tandem duplicated auxin-inducible genes across syntenic chromosomes of five plants, *V. vinifera*, *N. nucifera*, *S. polyrhiza*, *N. colorata*, and *A. trichopoda*. The aquatic sacred lotus (*N. nucifera*) also had significant tandem duplication of auxin-inducible genes. **d**, Proportion of auxin-inducible genes located in syntenic regions among total auxin-inducible genes. **e**, The expression profile of the auxin-inducible gene family in *N. colorata* at five time points: 6:00 am, 7:00 am, 10:00 am, 2:00 pm, and 4:00 pm.

7. Floral scent and colour in *N. colorata*

7.1 Explosive expansion of the terpene synthase gene family in *N. colorata*

Materials and Methods

Most plant terpenes are secondary metabolites, synthesized as a result of selective adaptation to multiple ecological niches. In plants, terpene synthase (TPS) genes form a mid-sized gene family[85], and they synthesize a diverse array of terpenes. We predicted the TPS genes using two hidden-Markov model seed sequences, the TPS N-terminal domain (PF01397) and the TPS metal-binding domain (PF03936), as search queries against the predicted proteome using hmmsearch in HMMER[72]. The search results were combined, overlapping sequences were filtered, and each sequence was manually curated to ensure that the gene length was accurate. For TPS identification in the water lily transcriptomes, sequences were manually screened to remove identical sequences to ensure that only unique genes were retained. Sequence alignment and phylogenetic tree construction were performed as described in the methods in the MADS-box section above. The sequence motif was drawn using WebLogo software with default parameters (http://weblogo.berkeley.edu/).

Results and Discussion

We performed the biosynthetic gene cluster analysis using plantiSMASH[86]. A number of gene clusters were predicted including those containing TPS genes (Supplementary Figure. 38) in part because many TPS genes are tandem duplicates. We found that some enzymes were clustered with the TPS gene, but did not find that p450 was clustered with TPS. According to the functional description of these enzymes, these gene clusters are not predicted to be on the pathway for synthesis of sesquiterpenes.

Through careful manual curation, we identified 93 TPS genes in the *N. colorata* genome. The phylogenetic tree classified them into the following previously established subfamilies: b, c, e/f, and g (Fig. 4b). *Amborella* TPS genes were classified into subfamilies b, c, e/f, g, and x, indicating that both *Amborella* and *N. colorata* lost or did not evolve subfamily a, which has members in monocots (*Oryza sativa* and *Sorghum bicolor*) and eudicots (*Arabidopsis thaliana* and *Populus trichocarpa*). The subfamilies c and e/f are involved in the biosynthesis of phytohormone gibberellins and other diterpenoids, the subfamilies b and g typically encode monoterpene synthases. The terpenoids as floral scent constituents of *N. colorata* are sesquiterpenes, which in monocots and eudicots are known to be produced by the TPS-a subfamily proteins. The lack of the TPS-a subfamily in the *N. colorata* genome suggests that the TPS-b/g subfamilies evolved new catalytic functions to produce sesquiterpenes in flowers.

There was a dramatic expansion of subfamily b in *N. colorata*, resulting in 86 TPS-b genes in this species (**Supplementary Table 20**). We also examined whether this expansion was unique to *N. colorata* or if it also encompassed other water lilies (**Supplementary Table 21**). In the different water lily species, subfamily b generally had more members than other subfamilies, suggesting that expansion of subfamily b may have occurred in the last common

ancestor of water lilies. Surprisingly, 62 subfamily-b TPS genes from *N. colorata* lacked the two conserved catalytic motifs 'DDxxD' and 'N/DDxxS/TxxxD/E' (**Supplementary Figures 39-40**), which are typically found in other known plant TPSs[85].

To investigate whether these genes are functional and where they are expressed, we performed sequence and expression analyses. From this analysis, we noticed the unusual explosion of the TPS-b subfamily with more than 80 members. Only six TPS genes showed expression in flowers, including one TPS-b subfamily. The *NC11G0123420* is the only gene to be highly expressed in the petal (Extended Data Fig. 7). We also found the *NC11G0123420* encoding protein has retained the two catalytic motifs (Extended Data Fig. 7), suggesting that it may have catalytic functions to produce sesquiterpenes in flowers of *N. colorata*.

Among fatty acid derivatives of *N. colorata* floral scent constituents is methyl decanoate (**Fig. 4a**), which has not been detected as floral scent compounds in monocots or eudicots[87]. As a fatty acid methylester, it could be conceived to be the product of methylation of the carboxyl group of decanoic acid. We hypothesized that it is biosynthesized by a SABATH methyltransferase[88]. An analysis of the expression patterns of the 12 *SABATH* genes in *N. colorata* indicated that NC11G0120830 has the highest level of expression in the petal (**Fig. 4c and Supplementary Fig. 41**). Therefore, it was the leading candidate responsible for the biosynthesis of methyl decanoate. To verify this prediction, a full-length cDNA of NC11G0120830 was cloned into a protein expression vector and the recombinant protein expressed in *Escherichia coli* was tested for methyltransferase activity using decanoic acid and a few other related fatty acids as substrates. NC11G0120830 exhibited the highest catalytic activity towards decanoic acid (**Fig. 4d**). Its product was verified to be the carboxyl methylester of decanoic acid (**Supplementary Fig. 42**), indicating that NC11G0120830 functions as a fatty acid methytransferase (FAMT) (**Supplementary Fig. 42**), a novel activity of the SABATH family. It was noted that NC11G0120830 could also use octanoic acid as substrate (**Fig. 4c**). Its product methyl octanoate is a minor constituent of the floral scent of *N. colorata* (**Fig. 4a**). The TPS and SABATH families might have played critical roles in the evolution of floral scent in water lilies. It also suggests parallel evolution of floral scent constituents in Nymphaeales and mesangiosperms.

Cluster	Record	Type	From	To	Size (kb)	Core domains	CD-HIT Clusters
Cluster 1	Chr1	Saccharide	8933038	8997718	64.68	AMP-binding, Glycos_transf_2	3
Cluster 2	Chr1	Terpene	35909415	35994683	85.27	Terpene_synth, Terpene_synth_C	6
Cluster 3	Chr10	Saccharide-Polyketide	12726555	12900013	173.46	Chal_sti_synt_C, Chal_sti_synt_N, UDPGT_2, p450	4
Cluster 4	Chr10	Saccharide	17056891	17219965	163.07	NAD_binding_1, UDPGT_2, UbiA	4
Cluster 5	Chr11	Saccharide	5781238	6087333	306.1	Epimerase, UDPGT_2, p450	3
Cluster 6	Chr11	Putative	7252017	7456158	204.14	Methyltransf_2, Peptidase_S10	5
Cluster 7	Chr12	Terpene	13875418	13979212	103.79	Methyltransf_7, Terpene_synth, Terpene_synth_C	3
Cluster 8	Chr13	Terpene	3628671	3876562	247.89	Amino_oxidase, Epimerase, Prenyltrans, SQHop_cyclase_C, SQHop_cyclase_N	3
Cluster 9	Chr13	Terpene	4607138	4707345	100.21	SQHop_cyclase_C, SQHop_cyclase_N	3
Cluster 10	Chr13	Terpene	5004755	5122848	118.09	SQHop_cyclase_C, SQHop_cyclase_N	3
Cluster 11	Chr13	Terpene	13491076	13527259	36.18	Terpene_synth, Terpene_synth_C	3
Cluster 12	Chr14	Polyketide	40859	498496	457.64	Chal_sti_synt_C, Chal_sti_synt_N, Transferase, adh_short	4
Cluster 13	Chr2	Putative	1012260	1182139	169.88	Epimerase, p450	4
Cluster 14	Chr2	Saccharide	8454982	8519597	64.61	UDPGT_2, p450	3
Cluster 15	Chr2	Saccharide	10219947	10609103	389.16	Epimerase, UDPGT_2	3
Cluster 16	Chr3	Saccharide	6591839	6727352	135.51	Epimerase, UDPGT_2	3
Cluster 17	Chr3	Saccharide-Alkaloid	18624254	18705027	80.77	Acetyltransf_1, Bet_v_1, Glycos_transf_2	3
Cluster 18	Chr3	Saccharide	22249341	22301442	52.1	2OG-FeII_Oxy, DIOX_N, Glycos_transf_2, UDPGT_2	3
Cluster 19	Chr3	Putative	25814929	25997119	182.19	Peptidase_S10, Transferase	4
Cluster 20	Chr3	Saccharide	28289224	28380360	91.14	2OG-FeII_Oxy, DIOX_N, UDPGT_2	4
Cluster 21	Chr4	Terpene	5442016	5497632	55.62	Terpene_synth, Terpene_synth_C	3
Cluster 22	Chr4	Saccharide-Terpene	14384768	14494163	109.39	Glycos_transf_1, Terpene_synth, Terpene_synth_C	3
Cluster 23	Chr4	Saccharide	19565836	19712418	146.58	Transferase, UDPGT_2, adh_short	3
Cluster 24	Chr4	Putative	24058106	24229165	171.06	Methyltransf_2, p450	4
Cluster 25	Chr4	Saccharide	24571695	24649611	77.92	SE, UDPGT_2	3
Cluster 26	Chr5	Polyketide	11014004	11296556	282.55	Chal_sti_synt_C, Chal_sti_synt_N, Epimerase, Methyltransf_11, Peptidase_S10	5
Cluster 27	Chr6	Polyketide	1211157	1345515	134.36	Chal_sti_synt_C, Chalcone, adh_short_C2	3
Cluster 28	Chr6	Terpene	14600994	14830169	229.18	Acetyltransf_1, Chalcone_2, Terpene_synth, Terpene_synth_C	3
Cluster 29	Chr6	Putative	19065087	19202365	137.28	adh_short, p450	4
Cluster 30	Chr6	Terpene	21461507	21576391	114.88	Terpene_synth, Terpene_synth_C	4
Cluster 31	Chr7	Putative	4670496	4904608	234.11	Acetyltransf_1, Amino_oxidase, COesterase, Epimerase	5
Cluster 32	Chr8	Saccharide	3334970	3460119	125.15	UDPGT_2, p450	3
Cluster 33	Chr8	Saccharide	14906122	14994573	88.45	Amino_oxidase, Methyltransf_11, UDPGT_2	4
Cluster 34	Chr9	Saccharide	11648959	11741802	92.84	Glycos_transf_1, SQS_PSY, adh_short_C2	3
Cluster 35	Chr9	Putative	22014437	22117918	103.48	Methyltransf_11, p450	5
Cluster 36	scaffold1	Terpene	4568478	4650176	81.7	Terpene_synth, Terpene_synth_C	

TPS gene cluster   Terpene synthesized by SQHop cyclase domain containing gene cluster

Supplementary Fig. 38 | Genome-wide clustering analyses of the *Nymphaea colorata* genes using plantiSMASH[86] reveal multiple tandem duplications of the TPS genes. 36 biosynthetic gene clusters were identified in the genome of *N. colorata*, of which 9 were TPS gene related clusters.

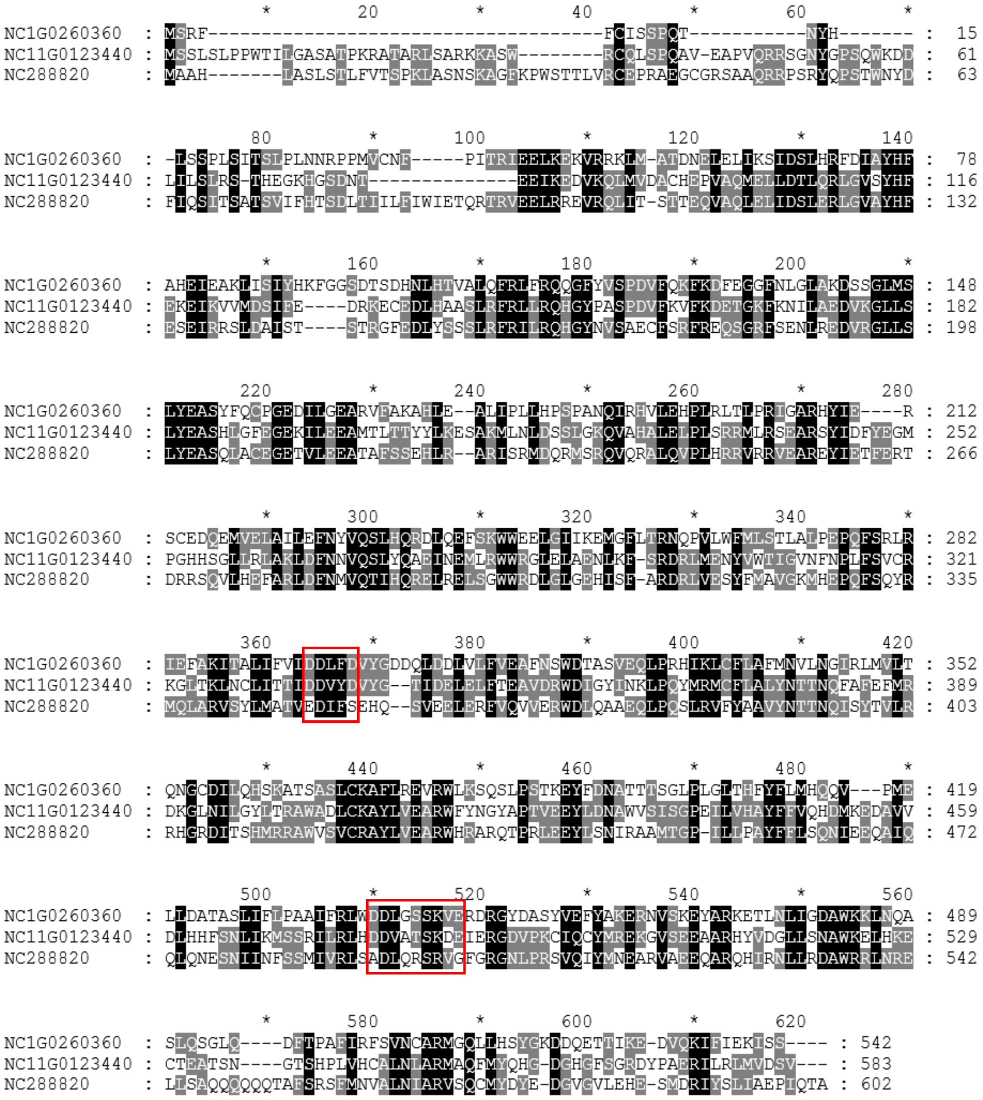

Supplementary Fig. 39 | Alignment of three representative terpene synthases (TPSs) from *Nymphaea colorata*. NC1G0260360 belongs to the TPS-g subfamily, and NC11G0123440 and NC288820 are TPS-b subfamily members. While both NC1G0260360 and NC11G0123440 contain two highly conserved catalytic motifs (red box), these motifs are absent in NC288820.

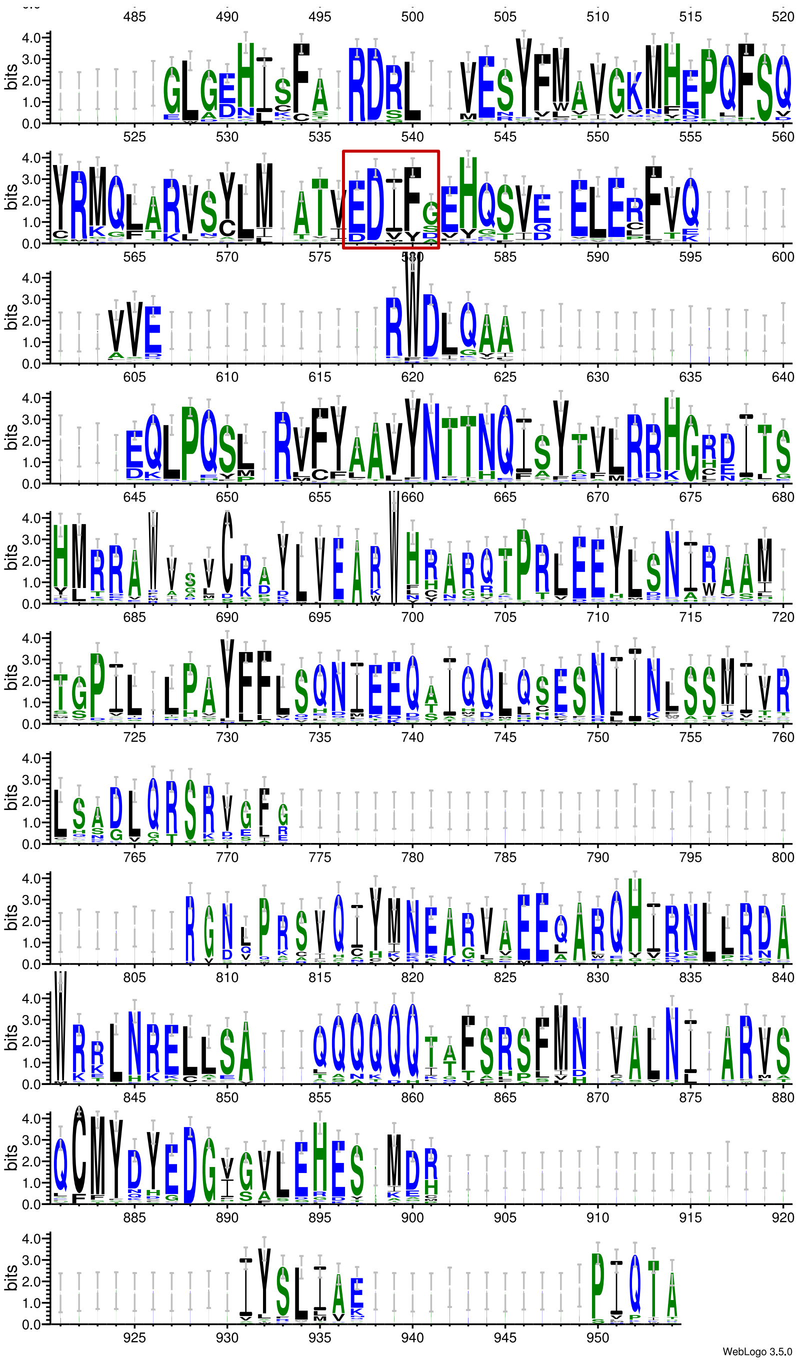

Supplementary Fig. 40 | WebLogo diagram for 62 subfamily-b terpene synthases from *Nymphaea colorata.* This diagram shows the region where the two conserved catalytic motifs 'DDxxD' and 'N/DDxxS/TxxxD/E' are typically found in known plant terpene synthases. Except for the gene NC11G0123420, the motif changed to 'EDxxx' and the second motif was completely absent in the rest subfamily b members.

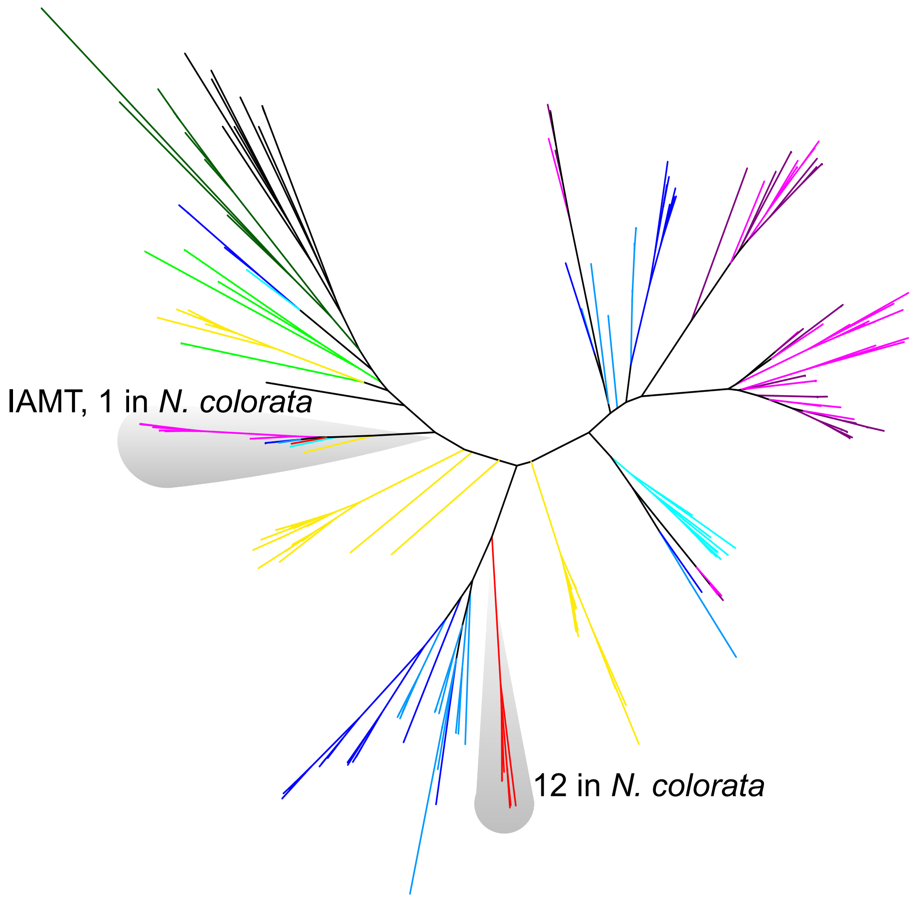

Supplementary Fig. 41 | A phylogenetic tree of SABATH methyltransferases from *Nymphaea colorata* and selected plants. IAMT, indole-3-acetic acid methyltransferase. Other species reference figure 4b.

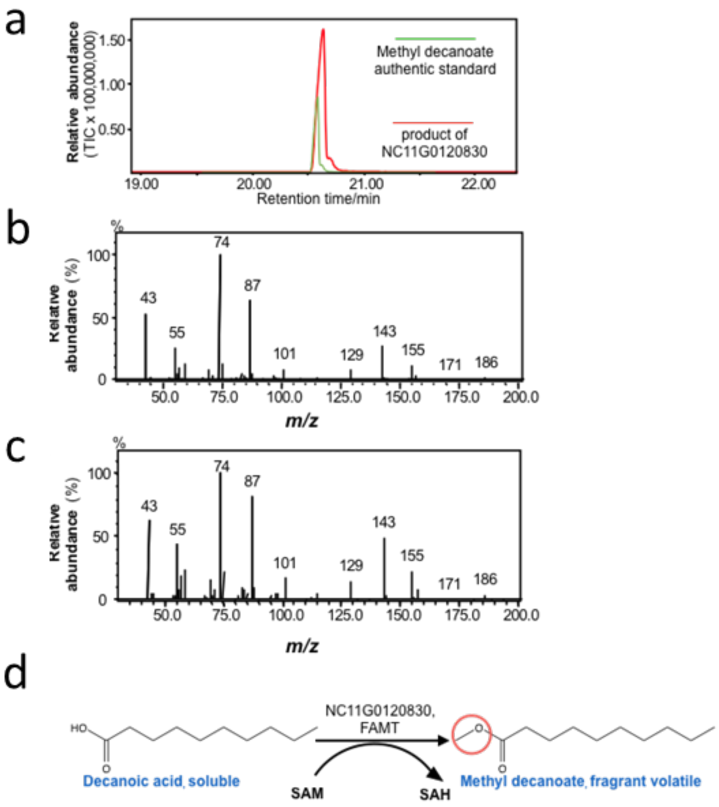

Supplementary Fig. 42 | Experimental validation of the catalytic product of NC11G0120830. a, the product of NC11G0120830 is methyl decanoate verified by authentic standard. Mass spectra of methyl decanoate authentic standard (**b**) and the product of NC11G0120830 using decanoic acid as substrate (**c**). **d,** Reaction scheme catalysed by NC11G0120830 as a fatty acid methyltransferase (FAMT) for the production of methyl decanoate using decanoic acid as substrate. SAM, *S*-adenosyl-L-methionine. SAH: *S*-adenosyl-homocysteine. Three biological repeats were performed independently with similar results.

7.2 Molecular basis of the blue floral pigment in *N. colorata* petals

Materials and Methods

Approximately 0.05 g of frozen dried petals of *N. colorata* were pulverized in liquid nitrogen, extracted with 1 mL of extracting solution (99.8: 0.2, v/v, methanol: formic acid) in a test tube, sonicated with KQ-500DE ultrasonic cleaner (Ultrasonic instruments, Jiangsu Kunshan, China) at 20 °C for 20 min, and then centrifuged in SIGMA 3K30 (SIGMA centrifuge, Germany) with 10,000 g for 10 min. The supernatants were collected into fresh tubes. The above operation was repeated three times. All extracts were combined and filtered through 0.22 μm reinforced nylon membrane filters (Shanghai ANPEL, Shanghai, China) before the I-Class ultra-high-performance liquid chromatography (I-Class UPLC) (Waters, USA) analysis. We made three replicates for each sample.

We used I-Class ultra-high-performance liquid chromatography/Xevo triple-quadrupole mass spectrometry (I-Class UPLC/Xevo TQ MS) for qualitative analysis. The liquid chromatograph was equipped with an ACQUITY UPLC HSS C18 column (2.1 mm · 100 mm, 1.7 μm) (Waters, USA). Eluent A was 1% formic acid aqueous solution and Eluent B was acetonitrile. The following gradient profile was used: 5% B at 0 min, 45% B at 6 min, 90% B at 7 min, 10% B at 7.1 min, 10% B at 10 min, 5% B at 10.2 min, 5% B at 13 min. The flow rate was 0.4 mL/min and the injected volume was 7 μL. Column temperature was maintained at 35 °C and sample temperature was 10 °C. Chromatograms of anthocyanidins and other flavonoids were acquired at 525 nm and 350 nm, respectively. We performed mass spectrometry with the following conditions: the positive-ion (PI) mode for anthocyanidins and negative-ion (NI) mode for other flavonoids; capillary voltage of 3.00 kV; cone voltage of 27 V for PI mode and cone voltage of 50 V for NI mode; desolvation gas (N_2) flow of 800 L/h; cone gas flow of 50 L/h; collision gas flow of 0.12 mL/min; collision energy of 23 eV; desolvation temperature of 400 °C; source temperature of 150 °C; and scanning range of 100–1000 (m/z) units.

Transcriptome data for blue-petals cultivars of *N. colorata* have been obtained from previous tissue transcriptome sequencing data. The transcriptome material of the white-petal cultivars was taken in the same way as the previous method, and we obtained the transcriptome data of the petals, carpel, sepal, stamens, leaves and roots of the white-petal cultivars. They were analysed to obtain expression values of each gene in different tissues.

For the qPCR quantification of floral color genes, the total RNAs from leave of 12 coloured water lilies (*N*. 'Perri', *N*. 'Kala sunlight', *N*. 'Moon light', *N*. 'Ox eye', *N*. 'Panama Pacific', *N*. 'Fox fire', *N*. 'Hilary', *N*. 'Danquanshi', *N*. 'Islamda', *N*. 'Indian red', *N*. 'campfire', and *N*. 'Ganna') were extracted. These include thee yellowish petal water lilies (*N*. 'Perri', *N*. 'Kala sunlight', and *N*. 'Moon light'), six bluish or purplish petal water lilies (*N*. 'Ox eye', *N*. 'Panama Pacific', *N*. 'Fox fire', *N*. 'Hilary', *N*. 'Danquanshi', and *N*. 'Islamda'), and three reddish water lilies (*N*. 'Indian red', *N*. 'campfire', and *N*. 'Ganna').

The reference genes for qPCR were AP47 (NC4G0238290) and ACT11 (NC13G0025720), which were chosen based on a previous study[89]. The specific primers were designed using Roche LCPDS2 software. For ACT11 gene NC13G0025720, the forward and reverse primers are GTCTGGATTGGAGGGTCTA and CTCATCATATTCTGCCTTCGC. For the AP47 gene NC4G0238290, the forward and reverse primers are ACAATCAAGGAATTGGGTAGG and CTGGCACTTTGACTACAACTC. For ANS gene NC9G0274510, the forward and reverse primers are CTTGATAATCCATGTGGGCG and CCTCACCTTCTCCTTGTTC. For the UDPGT gene NC8G0218160, the forward and reverse primers are CCAGCCGACCAACTGTAGATA and GCACTCTCTTTCCATTCGT. The reaction system was: 2*ChamQ SYBR qPCR Master Mix, 5 μL; 10 μM Forward primer, 0.2 μL; 10 μM Reverse primer, 0.2 μL; cDNA, 1 μL; Nuclease-free H_2O, 3.6 μL. PCR cycles 95 °C 30s; 95 °C 10 s, 60 °C 30 s, 40 cycles. Each study was repeated three times.

Results and Discussion

Analysis of the expression atlas of genes for the delphinidin-modification enzyme, Uridine Diphosphate glucuronyltransferase (UDPGT), showed that two genes in *N. colorata*

(NC3G0231100 and NC8G0211600) had the highest expression values in blue petals (Supplementary Fig. 43). We compared the genes involved the floral pigment biosynthetic pathway obtained from transcriptomes of the blue- and white-petal cultivars. The two UGTs also had the highest expression values in white petals and did not show significant expression bias between the blue and white cultivars. However, we identified two genes that have significantly higher expression in the blue petal than in white petal. One gene encodes anthocyanidin synthase (ANS, NC9G0274510) and the other encodes UDPGT (NC8G0218160). The predicted products of the proteins encoded by these two genes are the last two steps of this pathway; therefore, the two genes are the key genes for the synthesis of blue pigments in the petals, suggesting a potentially critical role of these genes in blue colouration.

Based on the qPCR analyses, we identified the expression of the ANS gene NC9G0274510 and the UDPGT gene NC8G0218160 across the 12 different petal-coloured water lilies (Supplementary Fig. 44). Using both reference genes as control, AP47 (NC4G0238290) and ACT11 (NC13G0025720), we found similar patterns that this ANS gene was only highly expressed in the petal of *N*. ‘Fox fire’, which has blue-purple petals. However, this UDPGT gene was highly expressed in all the bluish petal water lilies and had very low expression levels in the yellowish or reddish petal water lilies. These also suggest these two genes are potentially regulators responsible for synthesizing the blue anthocyanins in the petals.

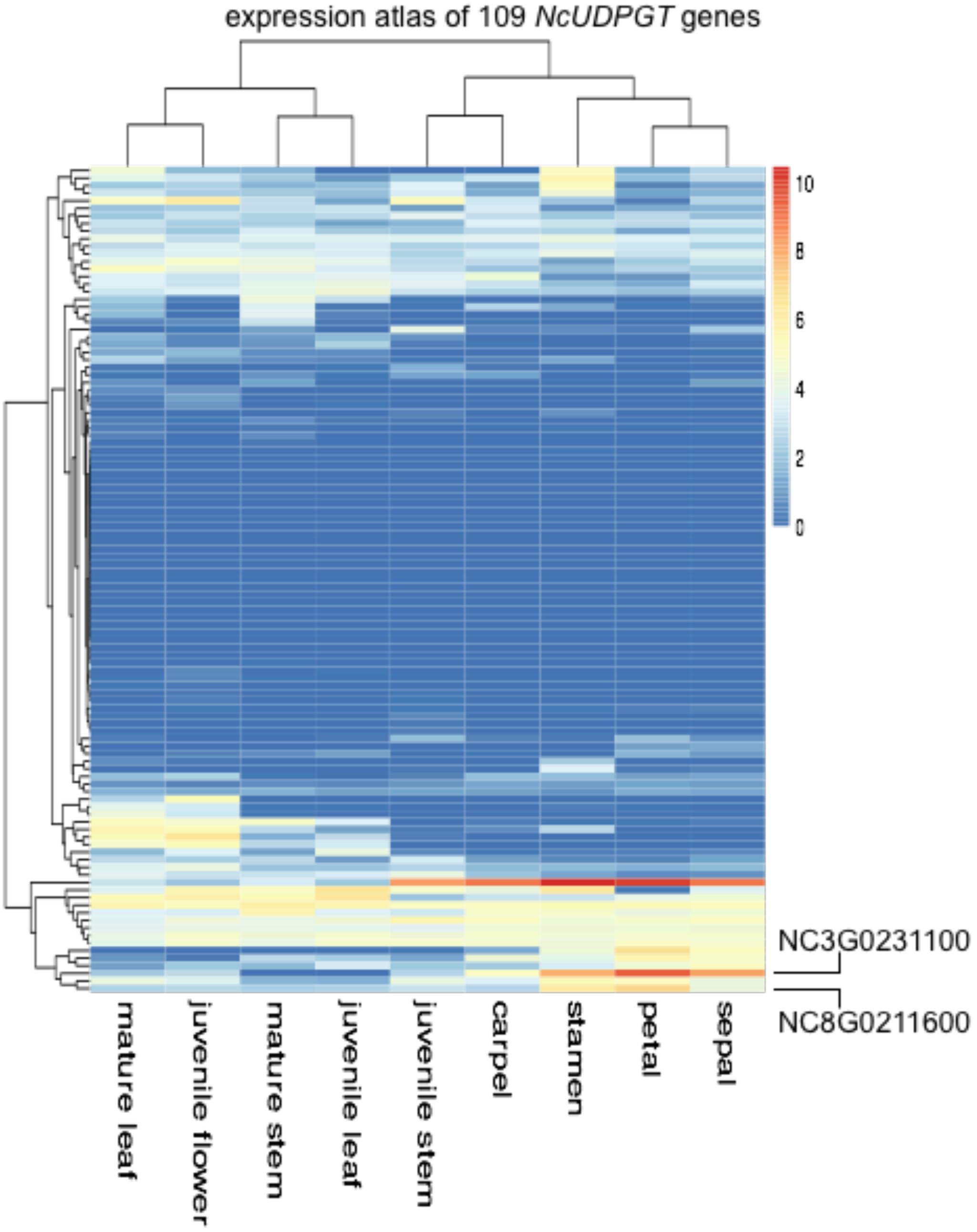

Supplementary Fig. 43 | Expressional profile of UDPGT genes from different organs of *Nymphaea colorata* (blue).

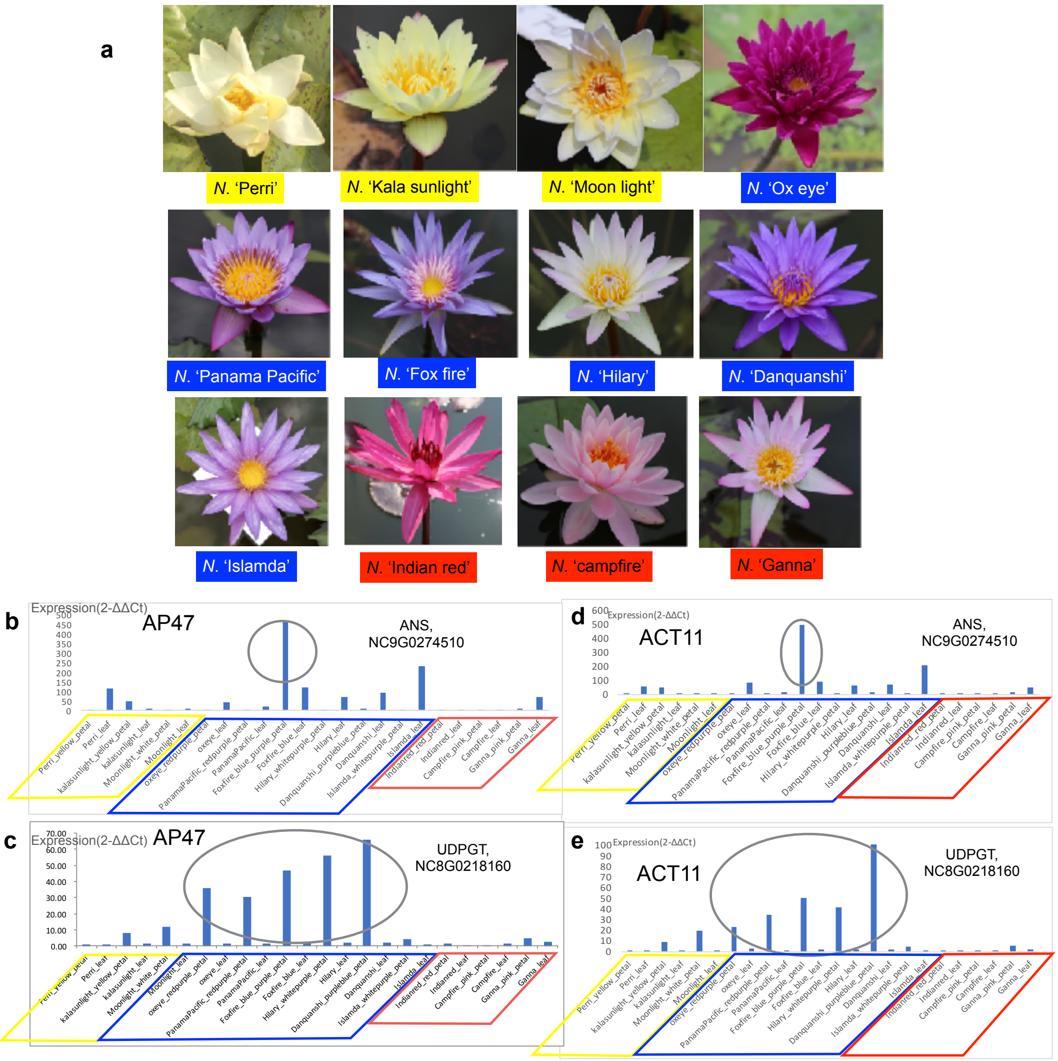

Supplementary Fig. 44 | qPCR based expression profile of ANS and UDPGT genes from water lilies with different petal colours. **a**, The sampled 12 water lilies with three major classes of petal colours. **b**, Using AP47 as the reference gene, the expression of NC9G0274510 among the 12 water lilies. **c**, Using AP47 as the reference gene, the expression of NC8G0218160 among the 12 water lilies. **d**, Using ACT11 as the reference gene, the expression of NC9G0274510 among the 12 water lilies. **e**, Using ACT11 as the reference gene, the expression of NC8G0218160 among the 12 water lilies. Three biological repeats were performed independently and the values shown are the average value of three repeats.

8. Genomic basis of stress and immune signalling in *N. colorata*

8.1 Expansion of *N. colorata* kinome

We observed significant expansions of immune and stress-related genes (Extended Data Fig. 9a) in *N. colorata* compared with *Amborella*. The angiosperm kinomes are usually larger than those in other land plants and other eukaryotes, with a multitude of functions including a significant role in plant immune and stress responses[90]. In *N. colorata*, we annotated a total of 1,148 kinase genes, vastly exceeding the 647 kinases found in *Amborella* (Supplementary Fig. 45).

In land plants, the kinome form the largest gene family, and can be divided into two groups, receptor-like kinases (RLKs) usually located in the membrane and soluble kinases (SKs) usually located in the cytosol[90]. RLKs are responsible for sensing and transducing the extracellular environmental signals into the cell, while SKs are responsible for the signal cascade and activation of target transcription factors, which in turn activate the target genes to respond to the environmental stimuli. The kinome represents the largest gene family in land plant genomes; for example, the *Arabidopsis* genome encodes 942 kinase genes[91], and the soybean kinome comprises 2,166 kinase genes[92].

In *N. colorata*, 1,148 kinase genes were annotated, which is 1.77 times greater than the 647 kinase genes in *Amborella* and also greater than the 1,008 kinases in *Arabidopsis* (the number in the TAIR 10 annotation). The *N. colorata* kinome is also larger than the kinomes of the following species: *Ricinus communis* (868), *Medicago truncatula* (911), *Cucumis sativus* (776), *Prunus persica* (1,024), *Citrus sinensis* (1,018), *Citrus clementina* (1,145), *Arabidopsis lyrata* (998), *Carica papaya* (601), *Vitis vinifera* (877), *Mimulus guttatus* (992), *Aquilegia coerulea* (981), *Brachypodium distachyon* (1,041), *Sorghum bicolor* (1,093), *Spirodela polyrhiza* (784), and *Zostera marina* (743). Phylogenetic inference and locus analyses confirmed that the WGD and tandem duplication events contributed to the accumulation of kinase genes in the water lily genome (**Supplementary Fig. 45**). Note the *N. colorata* genome encodes 754 RLK genes, which has more members than the eudicot grape and *Arabidopsis*.

a

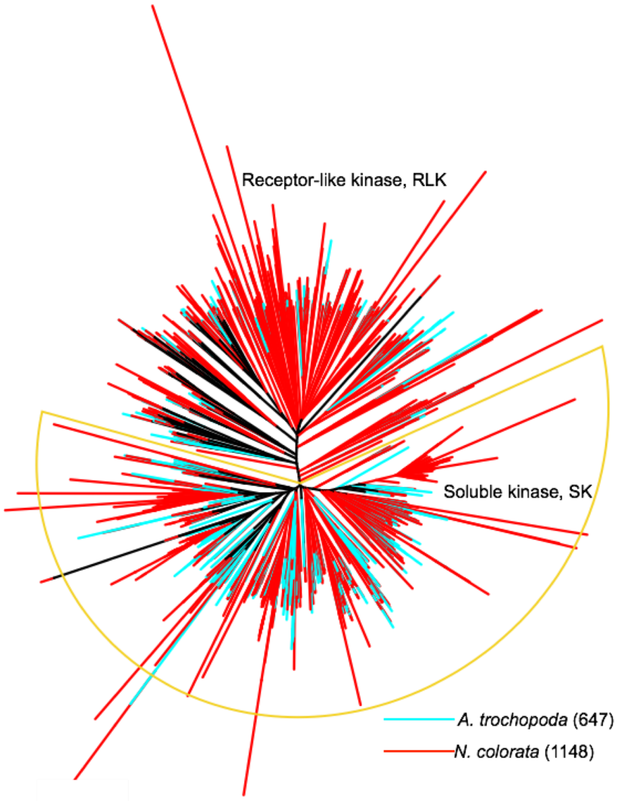

b

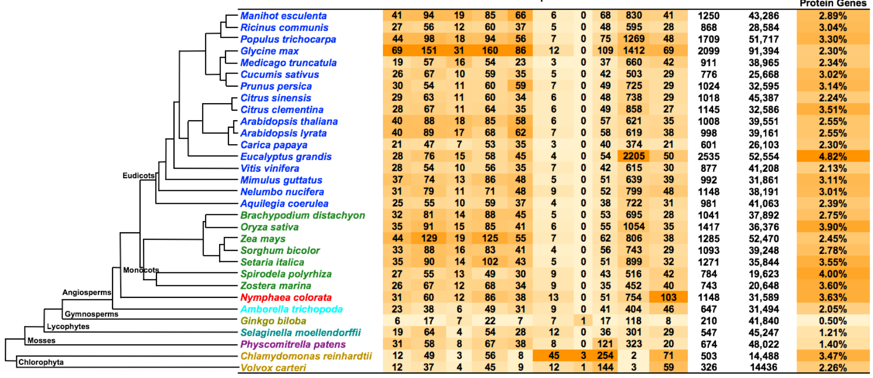

	AGC	CAMK	CK1	CMGC	STE	Plant-specific	TK	TKL	RLK_Pelle	Others	Total Pkinase	Total Protein Genes	Total Pkinase/Total Protein Genes
Manihot esculenta	41	94	19	85	66	6	0	68	830	41	1250	43,286	2.89%
Ricinus communis	27	56	12	60	37	5	0	48	595	28	868	28,584	3.04%
Populus trichocarpa	44	98	18	94	56	7	0	75	1269	48	1709	51,717	3.30%
Glycine max	69	151	31	160	86	12	0	109	1412	69	2099	91,394	2.30%
Medicago truncatula	19	57	16	54	23	3	0	37	660	42	911	38,965	2.34%
Cucumis sativus	26	67	10	59	35	5	0	42	503	29	776	25,668	3.02%
Prunus persica	30	54	11	60	59	7	0	49	725	29	1024	32,595	3.14%
Citrus sinensis	29	63	11	60	34	6	0	48	738	29	1018	45,387	2.24%
Citrus clementina	28	67	11	64	35	6	0	49	858	27	1145	32,586	3.51%
Arabidopsis thaliana	40	88	18	85	58	6	0	57	621	35	1008	39,551	2.55%
Arabidopsis lyrata	40	89	17	68	62	7	0	58	619	38	998	39,161	2.55%
Carica papaya	21	47	7	53	35	3	0	40	374	21	601	26,103	2.30%
Eucalyptus grandis	28	76	15	58	45	4	0	54	2205	50	2535	52,554	4.82%
Vitis vinifera	28	54	10	56	35	7	0	42	615	30	877	41,208	2.13%
Mimulus guttatus	37	74	13	86	48	5	0	51	639	39	992	31,861	3.11%
Nelumbo nucifera	31	79	11	71	48	9	0	52	799	48	1148	38,191	3.01%
Aquilegia coerulea	25	55	10	59	37	4	0	38	722	31	981	41,063	2.39%
Brachypodium distachyon	32	81	14	88	45	5	0	53	695	28	1041	37,892	2.75%
Oryza sativa	35	91	15	85	41	6	0	55	1054	35	1417	36,376	3.90%
Zea mays	44	129	19	125	55	7	0	62	806	38	1285	52,470	2.45%
Sorghum bicolor	33	88	16	83	41	4	0	56	743	29	1093	39,248	2.78%
Setaria italica	35	90	14	102	43	5	0	51	899	32	1271	35,844	3.55%
Spirodela polyrhiza	27	55	13	49	30	9	0	43	516	42	784	19,623	4.00%
Zostera marina	26	67	12	68	34	9	0	35	452	40	743	20,648	3.60%
Nymphaea colorata	31	60	12	86	38	13	0	51	754	103	1148	31,589	3.63%
Amborella trichopoda	23	38	6	49	31	9	0	41	404	46	647	31,494	2.05%
Ginkgo biloba	6	17	7	22	7	7	1	17	118	8	210	41,840	0.50%
Selaginella moellendorffii	19	64	4	54	28	12	0	36	301	29	547	45,247	1.21%
Physcomitrella patens	31	58	8	67	38	8	0	121	323	20	674	48,022	1.40%
Chlamydomonas reinhardtii	12	49	3	56	8	45	3	254	2	71	503	14,488	3.47%
Volvox carteri	12	37	4	45	9	12	1	144	3	59	326	14436	2.26%

c

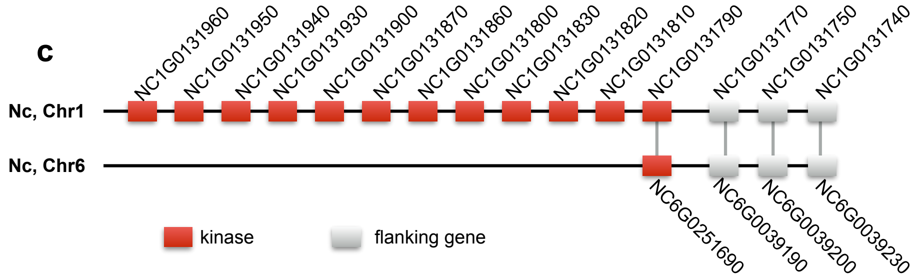

Supplementary Fig. 45 | The kinome tree of *Nymphaea colorata* and *Amborella trichopoda*. a, The tree was divided into two parts, receptor-like kinases usually located in the membrane and soluble kinases usually located in the cytosol. A total of 1,148 kinase genes were annotated in *N. colorata*, which is 1.77 times greater than the 647 kinases in *Amborella* and also more than the 1,026 kinases in *Arabidopsis* (we found a few more than in the previous report[91]). **b**, The distribution of kinase genes across the representative algae and land plants. The background colours indicate the numerical variation in each species. Note that *N. colorata* encodes the highest proportion of kinase genes

compared to gymnosperms and other earlier land plants. **c**, Example regions showing that tandem duplication and WGD contributed to the expansion of the *N. colorata* kinome.

8.2 R genes in the *N. colorata* genome

The plant R gene family is usually divided into three subfamilies according to their domain constitutions: TIR-NBS-LRR (TNL), CC-NBS-LRR (CNL), and RPW8-NBS-LRR (RNL)[93]. The 416 R genes in the *N. colorata* genome were divided into the three subfamilies as follows: 271 CNLs, 129 TNLs, and 16 RNLs. According to the phylogenetic tree constructed using multiple representative land plants (Extended Data Fig. 9b), CNLs and TNLs expanded significantly in *N. colorata*, compared with only 89 CNLs and 15 TNLs in *Amborella*. Since CNLs have been implicated in bacterial pathogen response in soybean and *Arabidopsis*[93], their expansion in *N. colorata* suggests that altered pathogen resistance may have contributed to the evolution of *N. colorata*. Similar to eudicots, there is a striking expansion of TNLs in *N. colorata* compared with *Amborella* (only 15 TNLs) and with none in 9 representative monocots. RNLs also expanded substantially in *N. colorata*, some eudicots, and gymnosperms, especially compared with 1 RNL in *Amborella*.

8.3 Anti-fungal domain-containing genes in *N. colorata*

Proteins with a stress-antifungal domain PF01657 (Pfam database, http://pfam.xfam.org) are involved in salt stress responses and have anti-fungal activities[94]. There were 102 anti-fungal domain-containing genes in *N. colorata*, in contrast with only 31 in the *Amborella* genome. The family of anti-fungal domain-containing genes was divided into six subfamilies according to the tree topology in this study. We found dramatic expansion of *N. colorata* genes (77 genes) in group V, in contrast with the 10 group V genes in *Amborella* (Supplementary Fig. 46). The dramatic expansion in the *N. colorata* genome suggests that these genes may have contributed to stress adaptation in this species. Compared with *Amborella,* we also found a dramatic expansion of the the xylanase inhibitor (TAXi domain) gene family in *N. colorata* (232 in *N. colorata* versus 70 in *Amborella*) (Supplementary Fig. 47).

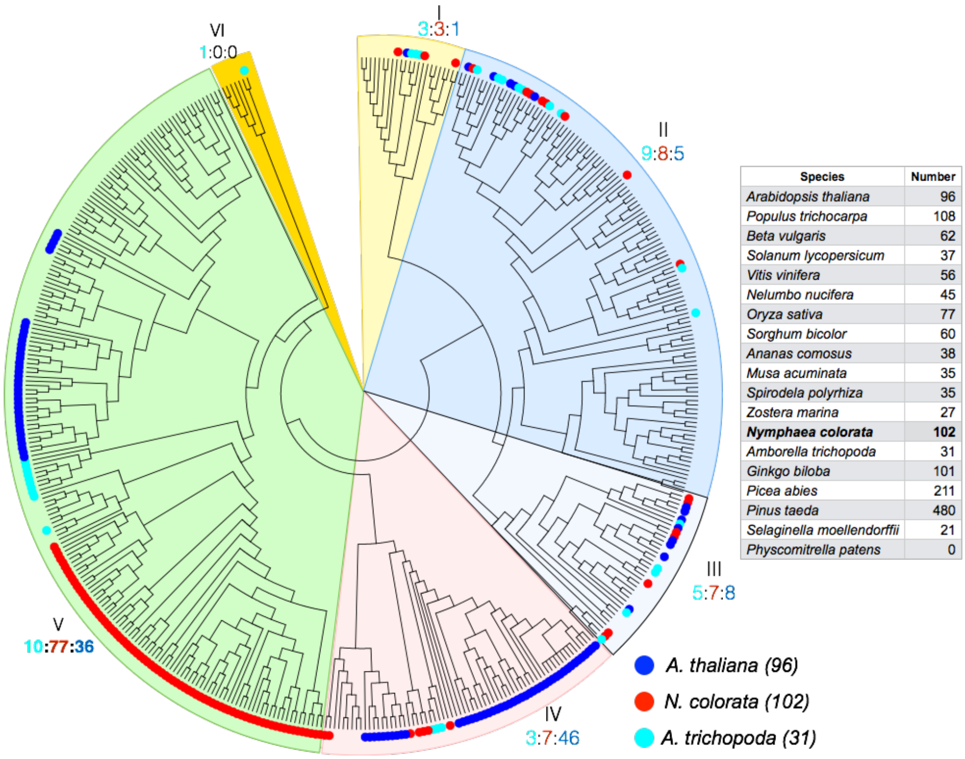

Species	Number
Arabidopsis thaliana	96
Populus trichocarpa	108
Beta vulgaris	62
Solanum lycopersicum	37
Vitis vinifera	56
Nelumbo nucifera	45
Oryza sativa	77
Sorghum bicolor	60
Ananas comosus	38
Musa acuminata	35
Spirodela polyrhiza	35
Zostera marina	27
Nymphaea colorata	**102**
Amborella trichopoda	31
Ginkgo biloba	101
Picea abies	211
Pinus taeda	480
Selaginella moellendorffii	21
Physcomitrella patens	0

Supplementary Fig. 46 | Phylogeny of anti-fungal domain-containing stress-related genes using sequences from *Amborella trichopoda*, *Nymphaea colorata*, and *Arabidopsis thaliana*.

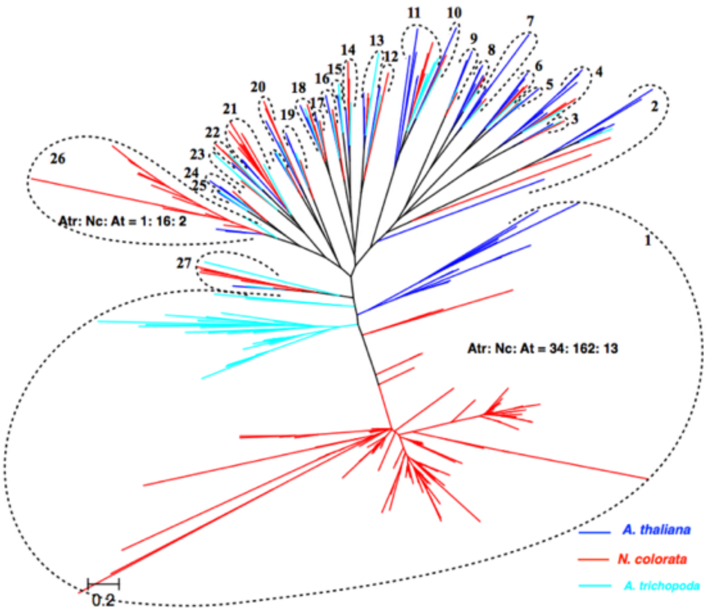

Species	Number
Arabidopsis thaliana	69
Populus trichocarpa	93
Beta vulgaris	53
Solanum lycopersicum	101
Vitis vinifera	613
Nelumbo nucifera	84
Oryza sativa	125
Sorghum bicolor	103
Ananas comosus	111
Musa acuminata	73
Spirodela polyrhiza	38
Zostera marina	46
Nymphaea colorata	**232**
Amborella trichopoda	70
Ginkgo biloba	65
Picea abies	192
Pinus taeda	380
Selaginella moellendorffii	43
Physcomitrella patens	38

Supplementary Fig. 47 | Phylogenetic tree of the TAXI domain-containing gene family. The tree was divided into 26 subfamilies according to the tree topology, with diversification at the ancestor of angiosperms. Explosive expansion was found in subfamily 1.

8.4 Expansion of the WRKY gene family in *N. colorata*

The WRKY transcription factor genes are widely distributed from chlorophyte algae to flowering plants, with roles in signalling pathways such as biotic and abiotic stress pathways as well as growth and development[95]. The *N. colorata* genome contains 69 WRKY genes, which is more than the 32 genes in *Amborella* and 39 in *Ginkgo* (Supplementary Fig. 48). The *N. colorata* WRKY genes fall into subfamilies I, IIa, IIb, IIc, IId, IIe, and III. We also found three WRKY gene clusters in the *N. colorata* genome, NC4G0199920-NC4G0199930-NC4G0199940-NC4G0199950-NC4G0199960, NC6G0255840-NC6G0255850-NC6G0255860, NC6G0255970-NC6G0255980-NC6G0255990, NC8G0124330-NC8G0124390-NC8G0124520, which could partially account for the large size of the WRKY family in *N. colorata*.

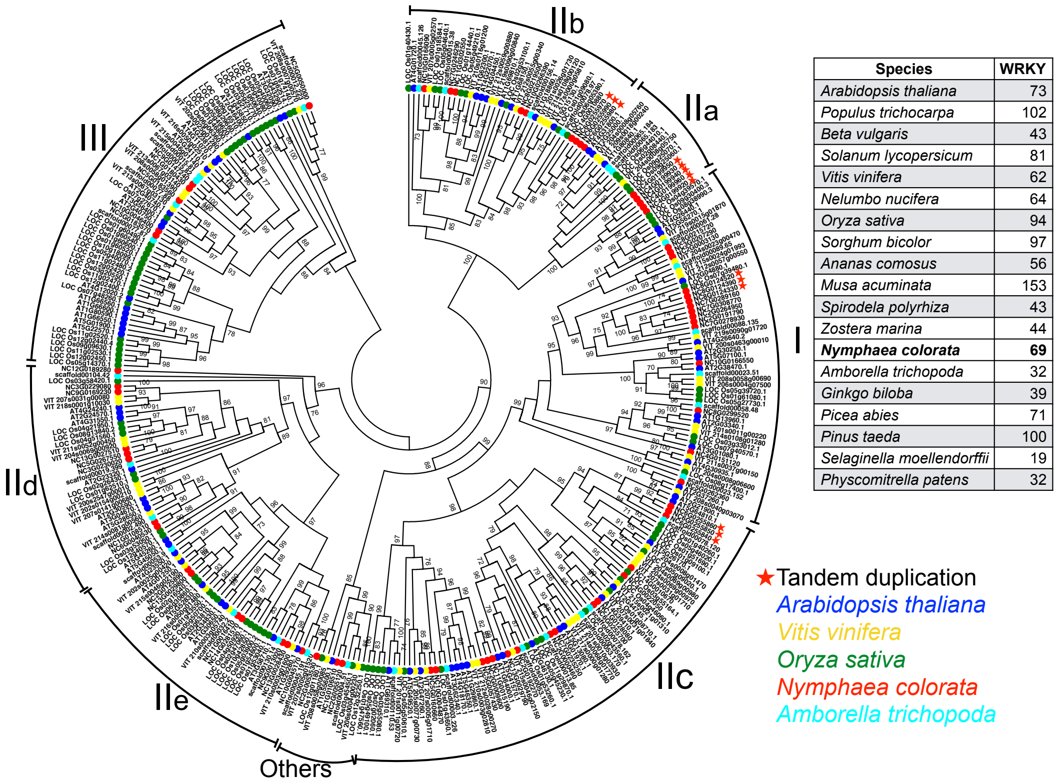

Species	WRKY
Arabidopsis thaliana	73
Populus trichocarpa	102
Beta vulgaris	43
Solanum lycopersicum	81
Vitis vinifera	62
Nelumbo nucifera	64
Oryza sativa	94
Sorghum bicolor	97
Ananas comosus	56
Musa acuminata	153
Spirodela polyrhiza	43
Zostera marina	44
Nymphaea colorata	**69**
Amborella trichopoda	32
Ginkgo biloba	39
Picea abies	71
Pinus taeda	100
Selaginella moellendorffii	19
Physcomitrella patens	32

Supplementary Fig. 48 | Phylogenetic tree of WRKY genes and their subfamily classification. WRKY transcription factors are components of plant signalling networks that regulate plant responses to biotic and abiotic stresses; they are also involved in plant developmental processes. WRKYs are well studied in the model plants *Arabidopsis* and rice. All transcription factors were compared with *Amborella*, and the number of WRKY genes in *N. colorata* was twice the number in *Amborella*. Subfamilies I, IIa, IIc, and IId had 6, 5, 6, and 8 WRKY genes, respectively. WRKYs from subfamilies I and IIc have been characterized as key transcription factors that regulate both biotic and abiotic stresses, and they are important players in plant defence responses as shown in *Arabidopsis* and rice. Therefore, the expanded WRKY family in *N. colorata* may have contributed to the wide adaptation of water lily compared with the narrow distribution of *Amborella*.

References

1 Saarela, J. M. *et al.* Hydatellaceae identified as a new branch near the base of the angiosperm phylogenetic tree. *Nature* **446**, 312-315 (2007).

2 Byng, J. W. *et al.* An update of the Angiosperm Phylogeny Group classification for the orders and families of flowering plants: APG IV. *Bot. J. Linn. Soc.* **181**, 1-20 (2016).

3 Christenhusz, M. J. M. & Byng, J. W. The number of known plants species in the world and its annual increase. *Phytotaxa* **261**, 201-217 (2016).

4 Borsch, T., Lohne, C. & Wiersema, J. Phylogeny and evolutionary patterns in Nymphaeales: integrating genes, genomes and morphology. *Taxon* **57**, 1052-1081 (2008).

5 Biswal, D. K., Debnath, M., Kumar, S. & Tandon, P. Phylogenetic reconstruction in the order Nymphaeales: ITS2 secondary structure analysis and *in silico* testing of maturase k (*matK*) as a potential marker for DNA bar coding. *BMC Bioinformatics* **13**, S26 (2012).

6 Sauquet, H. *et al.* The ancestral flower of angiosperms and its early diversification. *Nat. Commun.* **8**, 16047 (2017).

7 Chen, F. *et al.* Water lilies as emerging models for Darwin's abominable mystery. *Hort. Res.* **4**, 17051 (2017).

8 Doran, A. S., Les, D. H., Moody, M. L. & Phillips, W. E. *Nymphaea* 'William Phillips', a new intersubgeneric hybrid. *Hortscience* **39**, 446-447 (2004).

9 Pellicer, J., Kelly, L. J., Magdalena, C. & Leitch, I. J. Insights into the dynamics of genome size and chromosome evolution in the early diverging angiosperm lineage Nymphaeales (water lilies). *Genome* **56**, 437-449 (2013).

10 Wu, J. *et al.* The genome of the pear (*Pyrus bretschneideri* Rehd.). *Genome Res.* **23**, 396-408 (2013).

11 Servant, N. *et al.* HiC-Pro: an optimized and flexible pipeline for Hi-C data processing. *Genome Biol.* **16**, 259 (2015).

12 Koren, S. *et al.* Canu: scalable and accurate long-read assembly via adaptive *k*-mer weighting and repeat separation. *Genome Res.* **27**, 722-736 (2017).

13 Akdemir, K. C. & Chin, L. HiCPlotter integrates genomic data with interaction matrices. *Genome Biol.* **16**, 198 (2015).

14 Simao, F. A., Waterhouse, R. M., Ioannidis, P., Kriventseva, E. V. & Zdobnov, E. M. BUSCO: assessing genome assembly and annotation completeness with single-copy orthologs. *Bioinformatics* **31**, 3210-3212 (2015).

15 Albert, V. A. *et al.* The *Amborella* genome and the evolution of flowering plants. *Science* **342**, 1241089 (2013).

16 Levitsky, V. G. RECON: A program for prediction of nucleosome formation potential. *Nucleic Acids Res.* **32**, W346–W349 (2004).

17 Price, A. L., Jones, N. C. & Pevzner, P. A. *De novo* identification of repeat families in large genomes. *Bioinformatics* **21**, I351-I358 (2005).

18 Tempel, S. Using and understanding RepeatMasker. *Methods Mol. Biol.* **859**, 29-51 (2012).

19 Benson, G. Tandem repeats finder:a program to analyze DNA sequences. *Nucleic Acids Res.* **27**, 573-580 (1999).
20 Xu, Z. & Wang, H. LTR_FINDER: an efficient tool for the prediction of full-length LTR retrotransposons. *Nucleic Acids Res.* **35**, W265-W268 (2007).
21 Mizuno, H. *et al.* Sequencing and characterization of telomere and subtelomere regions on rice chromosomes 1S, 2S, 2L, 6L, 7S, 7L and 8S. *Plant J.* **46**, 206-217 (2006).
22 VanBuren, R. *et al.* Single-molecule sequencing of the desiccation-tolerant grass *Oropetium thomaeum*. *Nature* **527**, 508-511 (2015).
23 Melters, D. P. *et al.* Comparative analysis of tandem repeats from hundreds of species reveals unique insights into centromere evolution. *Genome Biol.* **14**, R10 (2013).
24 Shippen, D. E. & Mcknight, T. D. Telomeres, telomerase and plant development. *Trends Plant Sci.* **3**, 126-130 (1998).
25 Oliveira, L. C. & Torres, G. A. Plant centromeres: genetics, epigenetics and evolution. *Mol. Biol. Rep.* **45**, 1491-1497 (2018).
26 Langmead, B. & Salzberg, S. L. Fast gapped-read alignment with Bowtie 2. *Nat. Methods* **9**, 357-359 (2012).
27 Yang, X. Z. & Li, L. miRDeep-P: a computational tool for analyzing the microRNA transcriptome in plants. *Bioinformatics* **27**, 2614-2615 (2011).
28 Stifanic, M. & Batel, R. Genscan for *Arabidopsis* is a valuable tool for predicting sponge coding sequences. *Biologia* **62**, 124-127 (2007).
29 Stanke, M. & Morgenstern, B. AUGUSTUS: a web server for gene prediction in eukaryotes that allows user-defined constraints. *Nucleic Acids Res.* **33**, W465-W467 (2005).
30 Tian, T. *et al.* agriGO v2.0: a GO analysis toolkit for the agricultural community, 2017 update. *Nucleic Acids Res.* **45**, W122-W129 (2017).
31 Bundock, P. & Hooykaas, P. An *Arabidopsis hAT*-like transposase is essential for plant development. *Nature* **436**, 282-284 (2005).
32 Ke, M. Y. *et al.* Auxin controls circadian flower opening and closure in the waterlily. *BMC Plant Biol.* **18**, 143 (2018).
33 Xue, J. Y., Liu, Y., Li, L. B., Wang, B. & Qiu, Y. L. The complete mitochondrial genome sequence of the hornwort *Phaeoceros laevis*: retention of many ancient pseudogenes and conservative evolution of mitochondrial genomes in hornworts. *Curr. Genet.* **56**, 53-61 (2010).
34 Taylor, Z. N., Rice, D. W. & Palmer, J. D. The complete moss mitochondrial genome in the angiosperm *Amborella* is a chimera derived from two moss whole-genome transfers. *PLoS One* **10** (2015).
35 Goremykin, V. V., Hirsch-Ernst, K. I., Wolfl, S. & Hellwig, F. H. Analysis of the *Amborella trichopoda* chloroplast genome sequence suggests that Amborella is not a basal angiosperm. *Mol. Biol. Evol.* **20**, 1499-1505 (2003).
36 Bolger, A. M., Lohse, M. & Usadel, B. Trimmomatic: a flexible trimmer for Illumina sequence data. *Bioinformatics* **30**, 2114-2120 (2014).

37 Grabherr, M. G. *et al.* Trinity: reconstructing a full-length transcriptome without a genome from RNA-Seq data. *Nat. Biotechnol.* **29**, 644-652 (2011).
38 Zeng, L. *et al.* Resolution of deep angiosperm phylogeny using conserved nuclear genes and estimates of early divergence times. *Nat. Commun.* **5**, 4956 (2014).
39 Trapnell, C., Pachter, L. & Salzberg, S. L. TopHat: discovering splice junctions with RNA-Seq. *Bioinformatics* **25**, 1105-1111 (2009).
40 Trapnell, C. *et al.* Differential gene and transcript expression analysis of RNA-seq experiments with TopHat and cufflinks. *Nat. Protoc.* **9**, 2513-2513 (2014).
41 Friis, E. M., Pedersen, K. R. & Crane, P. R. Diversity in obscurity: fossil flowers and the early history of angiosperms. *Philos. T. R. Soc. B* **365**, 369-382 (2010).
42 Chen, F. *et al.* The sequenced angiosperm genomes and genome databases. *Front. Plant Sci.* **9**, 418 (2018).
43 Li, L., Stoeckert, C. J. & Roos, D. S. OrthoMCL: Identification of ortholog groups for eukaryotic genomes. *Genome Res.* **13**, 2178-2189 (2003).
44 Xiang, Y. *et al.* Evolution of rosaceae fruit types based on nuclear phylogeny in the context of geological times and genome duplication. *Mol. Biol. Evol.* **34**, 262-281 (2017).
45 Huang, C. H. *et al.* Resolution of Brassicaceae phylogeny using nuclear genes uncovers nested radiations and supports convergent morphological evolution. *Mol. Biol. Evol.* **33**, 394-412 (2016).
46 Katoh, K. & Standley, D. M. MAFFT multiple sequence alignment software version 7: improvements in performance and usability. *Mol. Biol. Evol.* **30**, 772-780 (2013).
47 Tamura, K., Stecher, G., Peterson, D., Filipski, A. & Kumar, S. MEGA6: molecular evolutionary genetics analysis version 6.0. *Mol. Biol. Evol.* **30**, 2725-2729 (2013).
48 Capella-Gutierrez, S., Silla-Martinez, J. M. & Gabaldon, T. trimAl: a tool for automated alignment trimming in large-scale phylogenetic analyses. *Bioinformatics* **25**, 1972-1973 (2009).
49 Suyama, M., Torrents, D., Bork, P. & Delbru, M. PAL2NAL: robust conversion of protein sequence alignments into the corresponding codon alignments. *Nucleic Acids Res.* **34**, W609-W612 (2018).
50 Mirarab, S. & Warnow, T. ASTRAL-II: coalescent-based species tree estimation with many hundreds of taxa and thousands of genes. *Bioinformatics* **31**, 44-52 (2015).
51 Stamatakis, A. RAxML-VI-HPC: maximum likelihood-based phylogenetic analyses with thousands of taxa and mixed models. *Bioinformatics* **22**, 2688-2690 (2006).
52 Yang, Z. PAML 4: Phylogenetic analysis by maximum likelihood. *Mol. Biol. Evol.* **24**, 1586-1591 (2007).
53 dos Reis, M. & Yang, Z. Approximate likelihood calculation on a phylogeny for Bayesian estimation of divergence times. *Mol Biol Evol* **28**, 2161-2172 (2011).

54 Rambaut, A., Drummond, A. J., Xie, D., Baele, G. & Suchard, M. A. Posterior summarization in Bayesian phylogenetics using Tracer 1.7. *Syst Biol* **67**, 901-904 (2018).
55 Smith, S. A. & O'Meara, B. C. treePL: divergence time estimation using penalized likelihood for large phylogenies. *Bioinformatics* **28**, 2689-2690 (2012).
56 Sanderson, M. J. r8s: inferring absolute rates of molecular evolution and divergence times in the absence of a molecular clock. *Bioinformatics* **19**, 301-302 (2003).
57 Wilgenbusch, J. C. & Swofford, D. in *Curr. Prot. Bioinfor.* Vol. 6.4.1-6.4.28 (2003).
58 Drummond, A. J., Suchard, M. A., Xie, D. & Rambaut, A. Bayesian phylogenetics with BEAUti and the BEAST 1.7. *Mol. Biol. Evol.* **29**, 1969-1973 (2012).
59 Gualberto, J. M. *et al.* The plant mitochondrial genome: dynamics and maintenance. *Biochimie* **100**, 107-120 (2014).
60 Van De Peer, Y., Mizrachi, E. & Marchal, K. The evolutionary significance of polyploidy. *Nat. Rev. Genet.* **18**, 411-424 (2017).
61 Vanneste, K., Baele, G., Maere, S. & Van de Peer, Y. Analysis of 41 plant genomes supports a wave of successful genome duplications in association with the Cretaceous-Paleogene boundary. *Genome Res.* **24**, 1334-1347 (2014).
62 Magallon, S., Hilu, K. W. & Quandt, D. Land plant evolutionary timeline: gene effects are secondary to fossil constraints in relaxed clock estimation of age and substitution rates. *Am. J. Bot.* **100**, 556-573 (2013).
63 Tank, D. C. *et al.* Nested radiations and the pulse of angiosperm diversification: increased diversification rates often follow whole genome duplications. *New Phytol.* **207**, 454-467 (2015).
64 Lohne, C. *et al.* Biogeography of Nymphaeales: extant patterns and historical events. *Taxon* **57**, 1123-1146 (2008).
65 Massoni, J., Couvreur, T. L. P. & Sauquet, H. Five major shifts of diversification through the long evolutionary history of Magnoliidae (angiosperms). *Bmc Evol Biol* **15**, 49 (2015).
66 Yoo, M. J., Bell, C. D., Soltis, P. S. & Soltis, D. E. Divergence times and historical biogeography of Nymphaeales. *Syst. Bot.* **30**, 693-704 (2005).
67 Salomo, K. *et al.* The emergence of earliest angiosperms may be earlier than fossil evidence indicates. *Syst Bot* **42**, 607-619 (2017).
68 Friis, E. M., Pedersen, K. R., von Balthazar, M., Grimm, G. W. & Crane, P. R. *Monetianthus Mirus* Gen. Et Sp Nov., a nymphaealean flower from the Early Cretaceous of Portugal. *Int. J. Plant Sci.* **170**, 1086-1101 (2009).
69 Taylor, D. W., Brenner, G. J. & Basha, S. H. *Scutifolium jordanicum* gen. et sp nov (Cabombaceae), an aquatic fossil plant from the Lower Cretaceous of Jordan, and the relationships of related leaf fossils to living genera. *Am. J. Bot.* **95**, 340-352 (2008).

70 Becker, A., Winter, K. U., Meyer, B., Saedler, H. & Theissen, G. MADS-box gene diversity in seed plants 300 million years ago. *Mol. Biol. Evol.* **17**, 1425-1434 (2000).
71 Chen, F., Zhang, X. T., Liu, X. & Zhang, L. S. Evolutionary analysis of MIKC^{c}-Type MADS-box genes in Gymnosperms and Angiosperms. *Front. Plant Sci.* **8**, 895 (2017).
72 Potter, S. C. *et al.* HMMER web server: 2018 update. *Nucleic Acids Res.* **46**, W200-W204 (2018).
73 D., F. R. *et al.* The Pfam protein families database: towards a more sustainable future. *Nucleic Acids Res.* **44**, D279-D285 (2016).
74 Price, M. N., Dehal, P. S. & Arkin, A. P. FastTree 2-approximately maximum-likelihood trees for large alignments. *PLoS One* **5**, e9490 (2010).
75 Xi, Z. X. *et al.* Phylogenomics and a posteriori data partitioning resolve the Cretaceous angiosperm radiation Malpighiales. *Proc. Natl. Acad. Sci. U. S. A.* **109**, 17519-17524 (2012).
76 Masiero, S., Colombo, L., Grini, P. E., Schnittger, A. & Kater, M. M. The emerging importance of type I MADS box transcription factors for plant reproduction. *Plant Cell* **23**, 865-872 (2011).
77 Schilling, S., Pan, S., Kennedy, A. & Melzer, R. MADS-box genes and crop domestication: the jack of all traits. *J. Exp. Bot.* **69**, 1447-1469 (2018).
78 Chanderbali, A. S., Berger, B. A., Howarth, D. G., Soltis, P. S. & Soltis, D. E. Evolving ideas on the origin and evolution of flowers: new perspectives in the genomic era. *Genetics* **202**, 1255-1265 (2016).
79 Alvarez-Buylla, E. R. *et al.* Flower development. *Arabidopsis Book* **8**, e0127 (2010).
80 Putterill, J., Laurie, R. & Macknight, R. It's time to flower: the genetic control of flowering time. *Bioessays* **26**, 363-373 (2004).
81 Wickland, D. P. & Hanzawa, Y. The *FLOWERING LOCUS T/TERMINAL FLOWER 1* gene family: functional evolution and molecular mechanisms. *Mol. Plant* **8**, 983-997 (2015).
82 Ruelens, P. *et al.* FLOWERING LOCUS C in monocots and the tandem origin of angiosperm-specific MADS-box genes. *Nat Commun* **4**, 2280 (2013).
83 Spartz, A. K. *et al.* SAUR inhibition of PP2C-D phosphatases activates plasma membrane H^{+}-ATPases to promote cell expansion in *Arabidopsis*. *Plant Cell* **26**, 2129-2142 (2014).
84 Atamian, H. S. *et al.* Circadian regulation of sunflower heliotropism, floral orientation, and pollinator visits. *Science* **353**, 587-590 (2016).
85 Chen, F., Tholl, D., Bohlmann, J. & Pichersky, E. The family of terpene synthases in plants: a mid-size family of genes for specialized metabolism that is highly diversified throughout the kingdom. *Plant J.* **66**, 212-229 (2011).
86 Kautsar, S. A., Duran, H. G. S., Blin, K., Osbourn, A. & Medema, M. H. plantiSMASH: automated identification, annotation and expression analysis of plant biosynthetic gene clusters. *Nucleic Acids Res.* **45**, W55-W63 (2017).

87 Knudsen, J. T., Tollsten, L. & Bergstrom, L. G. Floral scents-a checklist of volatile compounds isolated by head-space techniques. *Phytochem.* **33**, 253-280 (1993).
88 Zhao, N. *et al.* Structural, biochemical, and phylogenetic analyses suggest that indole-3-acetic acid methyltransferase is an evolutionarily ancient member of the SABATH family. *Plant Physiol.* **146**, 455-467 (2008).
89 Luo, H. L. *et al.* Candidate reference genes for gene expression studies in water lily. *Ana. Biochem.* **404**, 100-102 (2010).
90 Lehti-Shiu, M. D. & Shiu, S. H. Diversity, classification and function of the plant protein kinase superfamily. *Philos. T. R. Soc. B* **367**, 2619-2639 (2012).
91 Zulawski, M., Schulze, G., Braginets, R., Hartmann, S. & Schulze, W. X. The *Arabidopsis* kinome: phylogeny and evolutionary insights into functional diversification. *BMC Genomics* **15** (2014).
92 Liu, J. Y. *et al.* Soybean kinome: functional classification and gene expression patterns. *J. Exp. Bot.* **66**, 1919-1934 (2015).
93 Shao, Z. *et al.* Large-scale analyses of angiosperm nucleotide binding site-leucine-rich repeat genes. *Plant Physiol.* **170**, 2095-2109 (2016).
94 Zhang, L. *et al.* Identification of an apoplastic protein involved in the initial phase of salt stress response in rice root by two-dimensional electrophoresis. *Plant Physiol.* **149**, 916-928 (2009).
95 Chen, F. *et al.* The WRKY Transcription Factor Family in Model Plants and Crops. *Crit. Rev. Plant Sci.* **36**, 311-335 (2017).

Supplementary Table information

Supplementary Table 1 | The Nymphaeales samples used in this study.
Supplementary Table 2 | Genome size estimation based on *k*-mer number and coverage.
Supplementary Table 3 | Statistics of contig-level assembly of *N. colorata*.
Supplementary Table 4 | Genome completion evaluation using Benchmarking Universal Single-Copy Orthologs (BUSCO) analysis and Illumina read mapping rates, as well as mapping coverage of the leaf transcriptome.
Supplementary Table 5 | Statistics of the assembled 14 chromosomes of *N. colorata*.
Supplementary Table 6 | Genome assembly comparison among the *N. colorata* genomes, *Amborella* genome, and other genomes.
Supplementary Table 7 | Comparison of repetitive elements among representative flowering plants.
Supplementary Table 8 | Telomere and sub-telomere repeat locations and organization in the *N. colorata* genome.
Supplementary Table 9 | Centromere locations and organizations in the *N. colorata* genome.
Supplementary Table 10 | miRNA locations and organization in the contig of *N. colorata* genome.
Supplementary Table 11 | Comparison of gene length across representative land plants.
Supplementary Table 12 | The comparison of orthogroups among 19 representative land plants.
Supplementary Table 13 | Incomplete sampling causes phylogenetic discordance.

Supplementary Table 14 | Fossil calibrations used in this study.
Supplementary Table 15 | Mean age estimates (and 95% confidence intervals) of nodes of Interest across 100 bootstrap replicates.
Supplementary Table 16 | The gene duplicates produced by the whole genome duplication specific to the water lily.
Supplementary Table 17 | The numbers of gene families with anchor pairs from *N. colorata* that support the WGD before the divergence between *N. colorata* and *C. caroliniana* in different species.
Supplementary Table 19 | Gene expansions in the flower development network.
Supplementary Table 20 | The list of manually curated terpene synthase genes.
Supplementary Table 21 | Identification of terpene synthase genes in water lilies other than *N. colorata*.
