# Supplementary material for: The water lily genome and the early evolution of flowering plants
Source: Nature. 2019 Dec 18;577(7788):79–84. doi: 10.1038/s41586-019-1852-5 (PMC7015852; doi:10.1038/s41586-019-1852-5)
Supplement: Supplementary file 2 — Reporting Summary [file 41586_2019_1852_MOESM2_ESM.pdf]

nature research

Corresponding author(s): Liangsheng Zhang

Last updated by author(s): Sep 23, 2019

Reporting Summary

Nature Research wishes to improve the reproducibility of the work that we publish. This form provides structure for consistency and transparency in reporting. For further information on Nature Research policies, see Authors & Referees and the Editorial Policy Checklist.

Statistics

For all statistical analyses, confirm that the following items are present in the figure legend, table legend, main text, or Methods section.

n/a	Confirmed	
☒	☐	The exact sample size (*n*) for each experimental group/condition, given as a discrete number and unit of measurement
☒	☐	A statement on whether measurements were taken from distinct samples or whether the same sample was measured repeatedly
☒	☐	The statistical test(s) used AND whether they are one- or two-sided<br>*Only common tests should be described solely by name; describe more complex techniques in the Methods section.*
☒	☐	A description of all covariates tested
☐	☒	A description of any assumptions or corrections, such as tests of normality and adjustment for multiple comparisons
☒	☐	A full description of the statistical parameters including central tendency (e.g. means) or other basic estimates (e.g. regression coefficient) AND variation (e.g. standard deviation) or associated estimates of uncertainty (e.g. confidence intervals)
☒	☐	For null hypothesis testing, the test statistic (e.g. *F*, *t*, *r*) with confidence intervals, effect sizes, degrees of freedom and *P* value noted<br>*Give P values as exact values whenever suitable.*
☐	☒	For Bayesian analysis, information on the choice of priors and Markov chain Monte Carlo settings
☒	☐	For hierarchical and complex designs, identification of the appropriate level for tests and full reporting of outcomes
☒	☐	Estimates of effect sizes (e.g. Cohen's *d*, Pearson's *r*), indicating how they were calculated

Our web collection on statistics for biologists contains articles on many of the points above.

Software and code

Policy information about availability of computer code

Data collection	No software had been used for data collection
Data analysis	CANU (v1.3), Quiver, Arrow, Pilon (v1.20), bowtie2, Lachesis, HiC-Pro, Genscan, Augustus, Tandem Repeats Finder (v4.09), LTR_FINDER (v1.0.6), RepeatModeler (v1.0.5), RepeatMasker (v4.0.6), MAKER (v2.31.8), BUSCO software (v3.0.1), Tophat (v2.1.1), TRIMMOMATIC, Cufflink, TransDecoder, CD-HIT, Orthofinder (v1.1.8), Pfam (V31.0),MUSCLE(v3.8.31), TreePL, r8s, MCMCtree, ModelFinder, PAL2NAL (v14), trimAl (v1.4), RaxML (v8.0.19), PAML package (v4.4c), PhyML, ASTRAL(v5.5.12), PAUP(v4.0), InParanoid, LAST, BLAST, QUOTA-ALIGN, BEAST(v1.7), OrthoMCL (v2.0.9), Geneious (V.10.0.2), R, Python. Perl. Specific parameters used during run-time are provided in the methods. All softwares or scripts are available from official websites or GitHub as indicated in the methods.

For manuscripts utilizing custom algorithms or software that are central to the research but not yet described in published literature, software must be made available to editors/reviewers. We strongly encourage code deposition in a community repository (e.g. GitHub). See the Nature Research guidelines for submitting code & software for further information.

Data

Policy information about availability of data

All manuscripts must include a data availability statement. This statement should provide the following information, where applicable:

- Accession codes, unique identifiers, or web links for publicly available datasets
- A list of figures that have associated raw data
- A description of any restrictions on data availability

PacBio whole-genome sequencing data and Illumina data were deposited to the SRA at the NCBI under the BioProject ID PRJNA565347.
PacBio whole-genome sequencing data and Illumina data also were deposited in the BIG Data Center (http://bigd.big.ac.cn) under project number PRJCA001283.
The genome assembly sequences and gene annotations have been deposited in the Genome Warehouse in BIG Data Center under accession number GWHAAYW00000000 and in ENA BioProject (PRJEB34452). The genome assembly sequences and gene annotations have been also deposited in the Waterlily Pond (http://waterlily.eplant.org). All these data are freely available to the public.

Field-specific reporting

Please select the one below that is the best fit for your research. If you are not sure, read the appropriate sections before making your selection.

☒ Life sciences ☐ Behavioural & social sciences ☐ Ecological, evolutionary & environmental sciences

For a reference copy of the document with all sections, see nature.com/documents/nr-reporting-summary-flat.pdf

Life sciences study design

All studies must disclose on these points even when the disclosure is negative.

Sample size	No statistical methods were used to predetermine sample size. Our samples were all from wild type and did not use processed samples and groups.
Data exclusions	No data were excluded.
Replication	The genome sequence was taken and sequenced with more than 120 fold coverage. No replication is needed our genome reports.
Randomization	No random sampling is required for genome sequencing, because the genome differences are very small within the wild population, thus any wild plant is allowed for genome sequencing.
Blinding	Blinding is not applicable in our study because it does not involve subjects which receive different treatments.

Reporting for specific materials, systems and methods

We require information from authors about some types of materials, experimental systems and methods used in many studies. Here, indicate whether each material, system or method listed is relevant to your study. If you are not sure if a list item applies to your research, read the appropriate section before selecting a response.

Materials & experimental systems

n/a	Involved in the study	
☒	☐	Antibodies
☒	☐	Eukaryotic cell lines
☒	☐	Palaeontology
☒	☐	Animals and other organisms
☒	☐	Human research participants
☒	☐	Clinical data

Methods

n/a	Involved in the study	
☒	☐	ChIP-seq
☐	☒	Flow cytometry
☒	☐	MRI-based neuroimaging

Flow Cytometry

Plots

Confirm that:

☒ The axis labels state the marker and fluorochrome used (e.g. CD4-FITC).

☒ The axis scales are clearly visible. Include numbers along axes only for bottom left plot of group (a 'group' is an analysis of identical markers).

☒ All plots are contour plots with outliers or pseudocolor plots.

☒ A numerical value for number of cells or percentage (with statistics) is provided.

Methodology

Sample preparation	Nuclei were isolated from young leaves in spring ,using PI staining for 15 minutes.
Instrument	Beckman Coulter COULTER EPICS XL™
Software	FACS data analyses were performed using CXP v2.2 Software
Cell population abundance	abundance >8000 cells were collected for each sample. Total nuclei populations were gated using relative fluorescence intensity: the<br>proportions of nuclei with different ploidy levels were determined based on their relative fluorescence intensity: Pear is a diploid (2N) as a reference, according to the peak position (Supplementary Figure 5).

Gating strategy	Total nuclei populations were gated using PI intensity. In PI+ singles cells, the proportions of nuclei with different ploidy levels were determined based on their PI intensity (Supplementary Figure 5).

☒ Tick this box to confirm that a figure exemplifying the gating strategy is provided in the Supplementary Information.
